# Manual toothbrushing techniques for plaque removal and the prevention of gingivitis—A systematic review with network meta-analysis

**DOI:** 10.1371/journal.pone.0306302

**Published:** 2024-07-05

**Authors:** Renate Deinzer, Ulrike Weik, Zdenka Eidenhardt, Daniel Leufkens, Sonja Sälzer

**Affiliations:** 1 Department of Medicine, Institute of Medical Psychology, Justus Liebig University of Giessen, Giessen, Germany; 2 Department of Medicine, Institute of Medical Informatics, Justus Liebig University of Giessen, Giessen, Germany; 3 Clinic for Conservative Dentistry and Periodontology, School for Dental Medicine, Christian-Albrechts-University of Kiel, Kiel, Germany; 4 Privat Practice in Hamburg, Hamburg, Germany; Federal University of Minas Gerais: Universidade Federal de Minas Gerais, BRAZIL

## Abstract

**Background:**

The meaning of the toothbrushing technique for the effectivity of toothbrushing in terms of plaque removal and parameters of gingivitis is unknown. This systematic review and network meta-analysis (NMA) aimed to synthesize evidence from randomized controlled trials (RCTs).

**Methods:**

We searched MEDLINE (PubMed), the Cochrane Central Register of Controlled Trials, and the Web of Science for RCTs that compared any self-applied manual toothbrushing technique to any other technique or control and assessed plaque after toothbrushing and gingivitis. Where intervention effects were recorded repeatedly, the last post-intervention assessment was treated as the primary outcome date (POD), and the assessment closest to the intervention as the secondary outcome date (SOD). Age restrictions were not imposed. Participants with fixed orthodontic appliances were excluded. The evidence was evaluated using the Confidence in Network Meta-Analyses (CINeMA) approach, which is based on the Grading of Recommendations Assessment, Development and Evaluation (GRADE) approach.

**Results:**

Thirteen publications, including 15 studies, were identified. Ten studies assessing the Fones, Bass, and Scrub techniques provided data eligible for the NMA. The confidence rating of the evidence varied from very low to high in the case of plaque, and from very low to low in the case of gingivitis. Regarding PODs, Fones probably reduces plaque slightly compared with no training; the evidence is very uncertain that Fones may have little to no effect on gingivitis. Bass may result in little to no difference in plaque; the evidence that Bass may result in a slight increase in gingivitis is very uncertain. The evidence is very uncertain that Scrub may result in little to no difference in plaque at the SOD (no POD-data available) and that it may result in a slight increase in gingivitis.

**Conclusion:**

There is limited evidence regarding the effects of toothbrushing techniques on plaque after brushing or gingivitis.

## Introduction

Although most of the global population uses manual toothbrushes, there is still uncertainty regarding the effectiveness of different toothbrushing techniques. From a clinical perspective, evidence from good-quality randomized controlled trials (RCTs) is the best way to compare manual toothbrushing techniques for plaque removal and the prevention of gingivitis.

### Description of the condition

Dental plaque, a microbial biofilm covering the tooth surface, is a major etiological factor of periodontal diseases such as gingivitis and periodontitis. A few days of plaque accumulation at the gingival margin lead to inflammation of the adjacent gingiva. With further maturation of the microbial biofilm, the degree of inflammation increases; yet, removing the biofilm leads to the full restitution of gingival health within a few days [1, 2]. However, if the biofilm persists, the gingivitis becomes chronic. Chronic gingivitis may progress to the entire periodontium and thus affect additional periodontal structures such as the alveolar bone and desmodontium. At this stage, *restitutio ad integrum* is hardly possible to achieve. Insufficient plaque control and chronic gingivitis are therefore major risk factors for periodontitis [3, 4]. Hence, mechanical plaque control is one of the most important factors for its prevention.

### Description of the intervention and how it might work

Mechanical plaque control usually involves toothbrushing using a manual or powered toothbrush and the applying additional devices, such as tooth floss or interdental brushes, to remove plaque on the interdental surfaces [5]. The bristles of the toothbrush are thought to disturb the dental biofilm mechanically, thereby allowing its removal. Different brushing techniques may differ in their effectiveness in disturbing and removing biofilms.

Several manual toothbrushing techniques have been previously described. These techniques mainly differ in the movements of the brush (horizontal, vertical, circular) and the alignment of the bristles with respect to the gingiva and surface of the tooth. When cited in interventional studies, the intervention description must be carefully compared with the original description of the technique, as they may not match. Table 1 quotes the description of the most commonly recommended brushing techniques verbatim from the original publication on which it is based.

**Table 1:** Description of commonly recommended brushing techniques in the original publications.

Technique	movement	alignment/position
**Bass-Technique** [6]^1^	“Short back and forth strokes… At the same time teeth are cleaned above the gum in the sulci and between the teeth, as far as the bristles may go.” (p. 104)	“Bristles should be forced directly into the gingival crevices and into the sulci between the teeth, at about a 45-degree angle to the long way of the teeth” (p. 103–104)
**Modified Bass-Technique** [7]	“For this combined method one should have the patient perform several strokes using the Bass technic, and then sweep the gums and teeth using the roll method before moving to the next area.” (p. 131)	See Bass technique
**Fones-Technique** [8]	Buccal and labial surfaces: “…teeth nearly closed … with a fast circular motion the brush is swept backward and downward, reaching as far down on the lower gums as the brush can travel in its position, the forward and upward as high on the gums of the upper teeth as possible… the brush should travel in a perfect circle …very little pressure.” (p. 280)–reversing the stroke is allowed Lingual surfaces: “ends of the bristles should be placed against the gums of the right molar teeth, and the brush drawn straight forward until the heel wipes the lingual surfaces of the right incisors and cuspids and protrudes from the mouth for a short distance.” (p. 283)	Buccal and labial surfaces: “… ends of the bristles are lightly in contact with the gums of the upper molars.” (p. 280) Alignment not described
**Stillmann Technique** [9]	“Sufficient pressure is applied by bending the bristles slightly… The brush is lifted and attention is directed to the rapid inrush of blood… This act is repeated several times and the handle is given a slight rotary motion, but not enough to cause the bristle ends to move from the positions in which they were first placed.” (p. 319)	“… bristle ends resting partly on the gingivae and partly on the cervical portion of the teeth… The bristles are placed obliquely to the long axis of the tooth, or at an angle to the plane of the gingival surface and directed apically.” (p.319)
**Charters Technique** [10]	“With the bristles between the teeth, exert as much pressure as possible, giving the brush several slight rotary or vibratory movements, causing the sides of the bristles to come in contract with the gum margin and producing an ideal massage. Be careful not to make this movement sufficient to remove the bristles from between the teeth. After making three or for small circles, remove and replace in the same area. Make three or four applications in the same place.” (p. 90)	“Place the brush at right angles to the long axis of the teeth, the points of the bristles in contact with the surfaces; then gently force the bristles between the teeth, being careful not to pierce the gums, that is, do not to allow the points of the bristles to rest on the gums.” (p. 90)

^**1**^Bass published a first recommendation in 1948 [11], although without naming details such as the alignment. His later publication [6] is sometimes referred to as “modified Bass technique”. However, what is referred to commonly as the “modified Bass technique” also includes a sweeping movement from the gingiva to the coronal aspects that is not described by Bass, 1954 [6]. However Katz et al. [7] recommended to combine the Bass-Technique with such a movement and many authors refer to them when they describe the modified Bass technique.

### Why it is important to do this review

Severe periodontitis is the 6th most prevalent chronic disease worldwide, with a prevalence of 11.2%. [12]. It has a negative impact on oral health quality of life, speech, nutrition, confidence, and overall well-being and is independently associated with several systemic chronic inflammatory diseases [3]. Oral hygiene advice and training (OHA) is a major aspect of the preventing and treating periodontitis. However, few studies have assessed how to make OHAs most effective [13, 14]. The proposed cleaning procedures vary among dental societies, individual dental professionals, and manufacturers of oral hygiene products [15]. Furthermore, the capacity of dental laypersons to remove plaque appears to be low [16, 17], even when they brush to the best of their ability without a time limit [18–20]. Under the same conditions, dental professionals can access nearly full oral cleanliness but often cannot name the specific technique they apply [21]. Studies assessing children’s capacity to remove plaque indicate that the problems observed in adults originate during childhood [19, 22–24].

Powered toothbrushing has been observed to be advantageous compared to manual toothbrushing [5, 25] and could thus be considered an alternative, rendering this review superfluous. However, the differences are small and are mostly seen in RCTs comparing instructed powered toothbrushing to either uninstructed manual brushing or brushing using a specific technique (usually the modified Bass technique). A fair comparison between powered and manual toothbrushing should compare powered toothbrushing to the manual toothbrushing technique that was found to be the most effective. This review aims to identify this technique. Furthermore, when comparing the habitual application of manual and powered toothbrushes the clinical advantages of powered toothbrushing appear to vanish [18, 26]. Additionally, manual toothbrushing incurs lower costs than powered toothbrushing, both in terms of money and environmental resources, and is readily available to nearly everyone.

The current review aimed to examine RCTs assessing the effects of different self-applied manual toothbrushing techniques on plaque and gingivitis in children and adults. In doing so, it might inform which OHA regarding the brushing technique might lead to the best results and unveil further research needs in this respect. To the best of our knowledge, no other comprehensive review on the effectiveness of self-applied manual toothbrushing in children and adults is available.

A network meta-analysis (NMA), also known as mixed-treatment comparison meta-analysis, was conducted to compare the effects of different self-applied manual toothbrushing techniques on plaque and gingivitis. The NMA allows interventions to be compared even if they have not been directly compared in a trial. This can be done if these interventions were part of other trials where they had the same comparator (e.g. A vs. C is estimated from trials comparing A vs. B and B vs. C). As a result of this, both direct and indirect evidence can be calculated as an overall or network effect. This is a common approach to estimating differences between treatments, even if they cannot be observed directly [27–29].

### Objectives

#### Major objective

(1) To compare the effects of different self-applied manual toothbrushing techniques on plaque (primary outcome parameter) and gingivitis (secondary outcome parameter) in children and adults.

#### Secondary objectives

(2a) To assess whether the effects of self-applied manual toothbrushing techniques on plaque and gingivitis differ among children, adolescents, and adults.

(2b) To examine whether the effects on plaque differed according to the type of plaque index (focus on gingival margin vs. whole crown) assessed.

### Focused PICO question

In this systematic review a literature search and evaluation of randomized controlled studies of the following PICO question was performed: “What is the effect of any specific compared to any other or no specific self-applied manual toothbrushing technique on the parameters of plaque and gingivitis in humans of any age?”

## Materials and methods

The study protocol was registered in PROSPERO (https://www.crd.york.ac.uk/prospero/; Registration number CRD42022307534 [30]. This systematic review confirms to the PRISMA (Principals of Reporting of Systematic Reviews and Meta-analyses) extension statement for reporting systematic reviews incorporating network meta-analyses of healthcare interventions [31].

### Eligibility criteria and data items

#### Population

Humans of any age who can self-apply manual toothbrushing techniques.

Exclusion criteria: fixed orthodontic appliances.

#### Intervention

Any specific self-applied manual toothbrushing technique (SSAMTT)

Exclusion criteria: the application of the toothbrushing technique by a third party and powered toothbrushing.

#### Comparison

Any other specific SSAMTT (active control) or no specific technique (NST; inactive control).

Exclusion criteria: the application of the toothbrushing technique by a third party and powered toothbrushing.

#### Outcome

*Primary outcome parameter*. Dental plaque score after toothbrushing: If there is more than one plaque index, the one that focuses on plaque at the gingival margin is preferred.

*Secondary outcome parameter*. Gingivitis score: If there is more than one gingivitis index, the one focusing not only on the interdental sites is preferred.

If these outcomes were recorded on more than one date, the last date after the intervention was considered the *primary outcome date* and the first date after the intervention was considered the *secondary outcome date*.

Exclusion criteria: No information is available on any of these outcomes.

#### Study types

Randomized controlled studies.

### Information sources and search strategy

Three Internet sources were searched for appropriate randomized clinical trials related to research question: MEDLINE (via PubMed), the Cochrane Central Register of Controlled Trials (CENTRAL; CENTRAL identifies RCTs listed in MEDLINE, Embase, Cumulative Index to Nursing and Allied Health Literature (CINAHL), ClinicalTrials.gov, and WHO International Clinical Trials Registry Platform (ICTRP)), and Web of Science Core Collection. Where trial registers indicated unpublished trials, attempts were made to contact the respective principal investigators to obtain the trial results. All databases were searched for studies conducted before or during January 2022. Supplementary searches were conducted continuously from then on until November 2023. Table 2 shows the details of the search terms used.

**Table 2:** Search term in PubMed-Medline.

(((((((((((((((((((("bass"[MeSH Terms] OR "bass"[All Fields]) OR "stillman"[All Fields]) OR (((("charter"[All Fields] OR "charter s"[All Fields]) OR "chartered"[All Fields]) OR "chartering"[All Fields]) OR "charters"[All Fields])) OR "fones"[All Fields]) OR ((("scrub"[All Fields] OR "scrubbed"[All Fields]) OR "scrubbing"[All Fields]) OR "scrubs"[All Fields])) OR "roll"[All Fields]) OR ((("vertical"[All Fields] OR "verticality"[All Fields]) OR "vertically"[All Fields]) OR "verticals"[All Fields])) OR (("horizontal"[All Fields] OR "horizontally"[All Fields]) OR "horizontals"[All Fields])) OR "audio-tactile"[All Fields]) OR (((((("circular"[All Fields] OR "circularization"[All Fields]) OR "circularize"[All Fields]) OR "circularized"[All Fields]) OR "circularizes"[All Fields]) OR "circularizing"[All Fields]) OR "circulars"[All Fields])) OR (("circled"[All Fields] OR "circling"[All Fields]) OR "circlings"[All Fields])) OR ("circle"[All Fields] OR "circles"[All Fields])) OR ((((((((("rotate"[All Fields] OR "rotated"[All Fields]) OR "rotates"[All Fields]) OR "rotating"[All Fields]) OR "rotation"[MeSH Terms]) OR "rotation"[All Fields]) OR "rotations"[All Fields]) OR "rotational"[All Fields]) OR "rotator"[All Fields]) OR "rotators"[All Fields])) OR ((((((((("rotate"[All Fields] OR "rotated"[All Fields]) OR "rotates"[All Fields]) OR "rotating"[All Fields]) OR "rotation"[MeSH Terms]) OR "rotation"[All Fields]) OR "rotations"[All Fields]) OR "rotational"[All Fields]) OR "rotator"[All Fields]) OR "rotators"[All Fields])) OR ((((((("transversal"[All Fields] OR "transversally"[All Fields]) OR "transversals"[All Fields]) OR "transverse"[All Fields]) OR "transversed"[All Fields]) OR "transversely"[All Fields]) OR "transverses"[All Fields]) OR "transversing"[All Fields])) OR "wipe"[All Fields]) OR ((("wiped"[All Fields] OR "wipes"[All Fields]) OR "wiping"[All Fields]) OR "wipings"[All Fields])) OR "pick"[All Fields]) OR ("toothpick"[All Fields] OR "toothpicks"[All Fields])) OR (("angle"[All Fields] OR "angled"[All Fields]) OR "angles"[All Fields])) AND (((((("toothbrush"[All Fields] OR "toothbrushes"[All Fields]) OR "toothbrushing"[MeSH Terms]) OR "toothbrushing"[All Fields]) OR "toothbrushings"[All Fields]) OR (((("toothbrush"[All Fields] OR "toothbrushes"[All Fields]) OR "toothbrushing"[MeSH Terms]) OR "toothbrushing"[All Fields]) OR "toothbrushings"[All Fields])) OR "toothbrushing"[MeSH Terms])

The reference lists of the selected studies were screened for additional papers that met the eligibility criteria of this study.

### Selection and data collection process

The reviewers made at least two attempts to contact the corresponding author(s) to obtain missing information on the eligibility of the studies or other information required to describe and analyze the studies.

The titles and abstracts of the papers were independently screened by two reviewers (RD and SS). Only RCT trials that fulfilled the aforementioned PICO criteria were included and ineligible studies were excluded. Presumably eligible or unclear studies were reviewed in full text until it their eligibility could be assessed.

For those papers that the reviewers considered ineligible, they recorded at least one reason for exclusion. Disagreements were resolved through discussions. No third reviewer was needed to resolve disagreements as the two reviewers always reached a consensus. For studies that met all PICO criteria except the outcome, the authors were requested to indicate whether unpublished data fulfilling the criteria were available.

**Fig 1:**
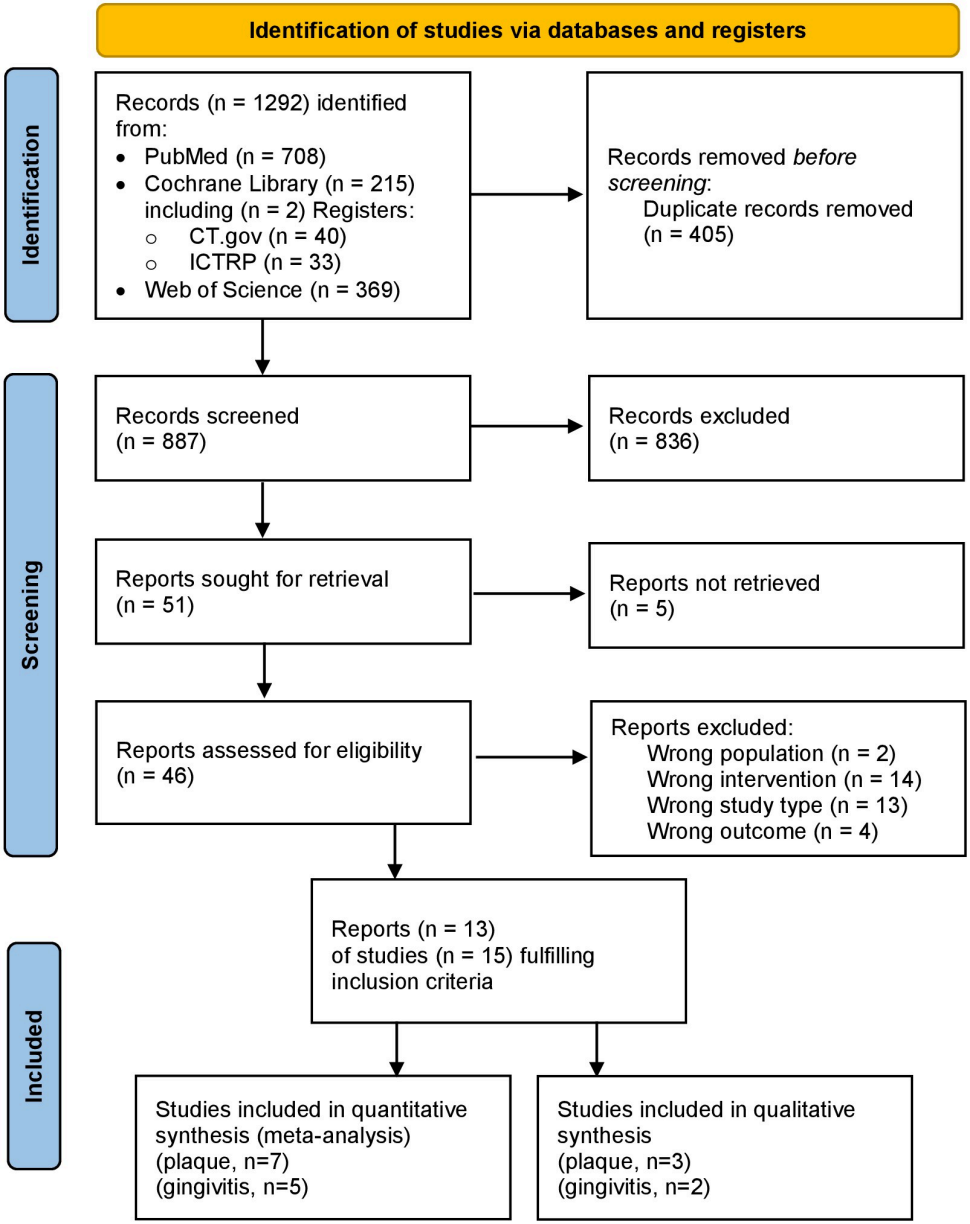
Flow chart of included studies according to the PRISMA 2020 statement [61].

The flow diagram (Fig 1) includes the number of titles and abstracts screened, number of full texts screened, number of full texts excluded, reasons for exclusion, number of full texts included, and number of studies included.

All relevant information from the selected studies was independently extracted by two other reviewers (UW & ZE). In cases of disagreement, the first two reviewers (RD and SS) were consulted. S1 Appendix shows the parameters and respective data of the selected studies.

### Risk of bias assessment

For each study two reviewers (UW & ZE) independently applied the revised version of the Cochrane risk of bias (RoB) tool [RoB 2, 32]. Any disagreements were resolved by discussion and, if necessary, by consulting two other reviewers (RD and SS). If insufficient information regarding judgment was available within the publication, authors were contacted for more information as described above.

### Assessment of indirectness on the study level

At the study level, the reviewers systematically evaluated two sources of indirectness: differences in interventions and outcome measures. According to the selection criteria, indirectness due to differences in the population could not occur.

#### Differences in interventions

Limitations in the validity of the intervention add to indirectness [33]. Two reviewers (UW and ZE) independently judged the following aspects that might affect the validity of the conditions. They contacted the authors when the published information was insufficient to make an assessment:

- Degree of standardization of the operationalization of the condition (study arms): The conditions were applied in a highly standardized (e.g., fully structured written/automatic), moderately standardized (e.g., semi-structured), or non-standardized manner (e.g., personal communication without further standardization).
- Comprehensibility of the operationalization of the condition: Is the operationalization described in sufficient detail to allow for exact replication (e.g., brushing technique named and exact procedure described), or is information missing (e.g., only naming one or all conditions without further description)?

The validity of the condition was judged as “no concerns” if there was a high degree of standardization and if the description was detailed enough to allow for replication.

The validity was judged “some concerns” if there was a moderate degree of standardization and if the description was detailed enough to allow for replication.

The validity was judged a “major concern” if either the degree of standardization or the comprehensibility was low.

#### Differences in outcome measures

In addition, the reviewers judged the validity of the outcome assessments and contacted authors if they required additional information to arrive at a decision. Limitations in the validity of outcome measures add to the indirectness [33]. This assessment considered the following information:

- Degree of standardization of the outcome assessment: Assessments were/were not made in a highly standardized manner (application of a specific index).
- Calibration of examiners: Examiners were calibrated, and information was provided regarding the calibration criterion they had to fulfil before assessments; it was described how examiners were calibrated, but the calibration criterion was unclear; examiners were not calibrated, or it was stated that examiners were calibrated, but no further information was provided regarding the calibration procedure.

The validity of the outcomes was judged as “no concerns” if the assessments were made in a highly standardized manner by calibrated examiners with information regarding the calibration criterion available and a fair calibration criterion (e.g., intraclass coefficient ICC>0.80; at least 80% agreement with another examiner).

The validity of outcomes was judged as “some concerns” if the assessments were made in a highly standardized manner and if a calibration procedure is described though the calibration criterion remains unclear.

The validity of the outcomes was judged a “major concern” if either the assessment was not highly standardized, examiners were not calibrated, or no further information regarding the calibration was available.

The overall rating of indirectness at the study level was set as high (major concerns) if the validity of intervention, the validity of the outcome assessment, or both were considered to raise major concerns. If both were considered to raise no concern, the overall rating was low concerns. In all the other cases, the overall rating of indirectness at the study level was set as some concerns [33]. The rating of indirectness at the study level informed the overall rating of a comparison, which was also based on the kind of comparisons (only indirect vs. mixed or only direct), which led to the NMA estimate of the comparison (for further details, see below).

### Statistical analysis and structured evaluation of the confidence into the evidence

For statistical analyses, it was first planned to rely on the RStudio Team software [34] with Netmeta [35, 36]. In addition, no further evaluation of the evidence was intended, besides the one delineated above. However, at the reviewers’ suggestion, a more comprehensive assessment of confidence in the results of the NMA has been amended, which was not pre-specified in the study protocol. To minimize the bias resulting from the post-hoc assessment and to make the analyses comprehensible, a semi-automatic, publicly available tool for the structured assessment of NMAs was used to support such analyses. This is an open-source software called Confidence in Network Meta-Analyses (CINeMA) that includes Netmeta [37, 38].

CINeMA considers six domains (RoB, Reporting bias, Indirectness, Imprecision, Heterogeneity, and Incoherence). It is based on the Grading of Recommendations Assessment, Development and Evaluation (GRADE) approach, which has recently been specified for NMA [39, 40]. CINeMA uses the outcome data of the studies, and manual evaluations of the authors regarding within-study bias and indirectness and allows for manual adjustments where necessary. The datasets provided for the CINeMA computations are published in the appendix of this study (S3 Appendix).

#### Statistical analysis via CINeMA

Statistical analyses within CINeMA were run with the following overall settings: inclusion of all interventions, random effects model, and standardized mean difference (SMD; [41]) as effect size. The random effects model was chosen because of the limited number of studies and their sample sizes [42, 43], as well as the goal of making the analyses as simple as possible. In addition to the overall heterogeneity (i.e., I2 and Tau2), subgroup analyses (i.e., z-values) also indicated significant heterogeneity within individual comparison groups (study-level covariates). However, comparison-adjusted funnel plots did not indicate small-study effects in our network (higher standard error, asymmetrically distributed around the zero line [43]). SMD, instead of mean difference (MD), was used as the effect size measure because very different indices can be used to assess primary and secondary outcomes. These indices exhibit different distribution characteristics. Accordingly, the resulting MDs are not directly comparable.

#### Structured evaluation of the confidence into the evidence via CINeMA

To evaluate the 6 domains, the following data were provided, and the following rules were applied:

- Within study bias: The results of the RoB 2 [32] judgement described in detail above were used to fill in the data for this domain. The option ‘average RoB’ was chosen for the overall estimation of the RoB for the respective comparison. Using this option, CINeMA multiplies the RoB scores of each study by its relative contribution to the network estimate and sums the results.
- Reporting bias: Within CINeMA reporting bias was initially set undetected for all comparisons where data of all registered studies identified were available. The setting was manually changed to suspected when evidence was available for trials that could not be included in the NMA because it was not possible to obtain sufficient statistical information [44].
- Indirectness: For each study indirectness was rated by the reviewers as described above. The option ‘average indirectness’ was chosen for the overall estimation of indirectness for the respective comparisons. CINeMA computes average indirectness by multiplying the indirectness scores of the studies by their contributions to the respective network estimates. Subsequently, the level of indirectness was manually set to high if indirect comparisons were the only source of the NMA estimate [33].
- Imprecision: The degree of imprecision was estimated by CINeMA based on confidence intervals and their relationship to the predefined minimal clinically important effect. In the absence of other indicators, an SMD of 0.2 was used as the minimum difference considered clinically important. This corresponds to the convention set up by Cohen [45] that SMDs of 0.2 reflect small effect sizes.
- Heterogeneity: The degree of heterogeneity was assessed using the built-in procedure of CINeMA with the setting as that for imprecision (SMD of 0.2, the minimum difference considered clinically important). CINeMA computes the prediction intervals and compares them with the confidence intervals. Concerns emerge if these intervals would lead to different clinical decisions [38]. The degree of heterogeneity is poorly estimated if there are very few trials [37].
- Incoherence: The degree of incoherence was assessed using the built-in procedure of CINeMA. CINeMA automatically judges the inconsistency based on two computations: the separating indirect from direct evidence method [38] for network estimates containing mixed evidence from direct and indirect comparisons and the design by treatment interaction test for estimates containing only one type of comparison.

To evaluate the overall confidence in the evidence for each comparison, the confidence in the evidence was initially set ‘high.’ It was downgraded to ‘moderate’, ‘low’, or ‘very low’ according to the following rules: Concerns regarding within-study bias always led to a downgrade of one step if there was some concern and two if there were major concerns. Due to the small number of trials, the authors decided not to conduct sensitivity analyses to see whether the estimates from trials with a high RoB differed from those with a low RoB [37]. Concerns about reporting bias of any degree led to a one-step downgrade. Major concerns regarding indirectness led to a downgrade in one step, whereas ‘some concerns’ were ignored for the final rating. Because imprecision, heterogeneity, and incoherence are interrelated [37] they were evaluated jointly. If either raised major concerns, the confidence rating was downgraded two steps; if imprecision or the two others raised some concerns, the confidence rating was downgraded one step. The reasons for the down-gradings are documented in the respective outputs. For the informative statements to communicate the findings, the suggestions from the GRADE working group were considered [46].

#### Handling of missing data

Missing data were handled according to the Cochrane Handbook [47]. The only publications that reported no standard deviations (SDs) were from the laboratory of one of the authors (RD), who could readily provide the SDs for the analyses by looking into the protocols of the original statistical analyses [48–50]. In cases where only absolute or relative declines in plaque from before to after brushing were reported, attempts were made to obtain access to the levels assessed after toothbrushing, either by contacting the study authors (1^st^ choice) or (when 1^st^ choice was not successful) by estimating the post-brushing levels from the data available in the publication. In cases where statistics were available at the tooth level but not at the participant level, the authors were contacted to obtain participant-level information.

#### Additional analyses

Additional Analyses were not performed due to the limited number of comparisons available for the NMA.

## Results

### Search and selection results

The search resulted in a total of 1,292 records, and after the removal of duplicates, 887 unique papers were screened by title and abstract (Fig 1). Of the remaining 51 reports, five could not be obtained in full. The titles of these records indicate that they did not fully meet the inclusion criteria as no comparison was mentioned. Thirty-three papers were excluded after reading the full text because they did not meet the PICO criteria (see S2 Appendix for details). Accordingly, the search yielded 13 full-text papers [48–60] (see Table 3 and S1 Appendix). They comprise 15 studies since the publication of Ceyhan et al. [52] described three independent studies with different participants.

**Table 3:** Overview over main study characteristics.

	Design		Out-come		Examiner blinding			Age of participants				Control condition			Brushing technique				
	parallel	cross over	plaque after brushing	gingivitis	yes	no information available	no	Young adults (18–35 years)	Adults and seniors (30–82 years)	Adults (> 18 years; no further info)	children (4–12 years)	general instructions without a specific technique	no instructions at all	only comparison of different techniques	Bass/modified Bass	Fones	Scrub	Roll	Vertical brushing
Ausenda et al.[51]	x			x	x					x		x			x				
Ceyhan et al.[52]	x		x		x						x			x		x	x		
Deinzer et al.[48]	x		x		x				x			x			x	x			
Dosumu et al.[53]	x			x	x			x				x			x	x			
Harnacke et al.[49]	X		x	x	x			x				x			x	x			
Harnacke et al.[50]	x		x	x	x			x				x			x	x			
Janakiram et al.[54]	x			x	x			x				x			x				
Kanchanakamol et al.[55]	x		x			x		x						x	x			x	
Schlueter et al.[57]	x		x				x	x					x		x				
Schmalz et al.[58]	x			x	x			x				x				x			
Smutkeeree et al.[59]	x			x		x					x			x	x		x		
Sarvia et al.[56]	x		x			x					x	x					x		
Zhang et al.[60]		x	x		x			x						x	x				x

For NMA, seven studies could be included for parameters of plaque (see Table 4) and five for parameters of gingivitis (see Table 6). Reasons for not being included in the NMA were missing patient-based data [51] and missing statistical information for group comparisons (see Table 8) [53, 55, 56, 60].

**Table 4:** Comparisons available for NMA regarding the primary outcome parameter: Plaque levels after toothbrushing.

Outcome date/Study	Index	Weeks after intervention	1st study arm compared				2nd study arm compared				standardized mean difference				
			Intervention	n*	mean	SD	Intervention	n*	Mean	SD	SMD	SE	CI (95%) lb	CI (95%) ub	p
**Primary outcome date (Last date of assessment)**															
Deinzer et al. 2016; natural teeth [48]	MPI	12	NST	30	70.04	16.00	Fones	32	71.89	15.29	-0.12	0.25	-0.62	0.38	0.646
			NST	30	70.04	16.00	Bass	30	67.18	18.21	0.16	0.26	-0.34	0.67	0.524
			Fones	32	71.89	15.29	Bass	30	67.18	18.21	0.28	0.26	-0.22	0.78	0.277
Deinzer et al. 2016; crowned teeth [48]	MPI	12	NST	30	34.34	20.13	Fones	32	31.90	20.35	0.12	0.25	-0.38	0.62	0.640
			NST	30	34.34	20.13	Bass	30	31.23	22.73	0.14	0.26	-0.36	0.65	0.580
			Fones	32	31.90	20.35	Bass	30	31.23	22.73	0.03	0.25	-0.47	0.53	0.904
Harnacke et al. 2012 [49]	MPI	28	NST	19	58.71	18.63	Fones	19	52.57	17.01	0.34	0.33	-0.30	0.98	0.302
			NST	19	58.71	18.63	Bass	18	63.59	17.03	-0.27	0.33	-0.91	0.38	0.419
			Fones	19	52.57	17.01	Bass	18	63.59	17.03	-0.63	0.34	-1.29	0.03	0.060
Harnacke et al. 2016 [50]	MPI	28	NST	22	78.89	10.66	Fones	23	72.87	15.69	0.44	0.30	-0.15	1.03	0.146
			NST	22	78.89	10.66	Bass	23	80.93	13.39	-0.17	0.30	-0.75	0.42	0.580
			Fones	23	72.87	15.69	Bass	23	80.93	13.39	-0.54	0.30	-1.13	0.05	0.070
Schlueter et al 2013 [57]	TQHI	4	NST	27	1.72	0.48	Bass^1^	24	1.52	0.58	0.37	0.28	-0.18	0.93	0.189
			NST	27	1.72	0.48	Bass^2^	26	1.50	0.69	0.37	0.28	-0.18	0.91	0.187
**Secondary outcome date (First date of assessment)**															
Ceyhan et al. 2018; 1 [52]^+^	PI	0	Scrub	17	0.30	0.04	Fones	21	0.25	0.04	1.22	0.36	0.53	1.92	0.001
Ceyhan et al. 2018; 2 [52]^+^	PI	0	Scrub	22	0.37	0.03	Fones	25	0.47	0.04	-2.76	0.41	-3.55	-1.96	0.000
Ceyhan et al. 2018; 3 [52]^+^	PI	0	Scrub	39	0.23	0.02	Fones	39	0.18	0.02	2.48	0.30	1.89	3.07	0.000
Deinzer et al. 2016; natural teeth [48]	MPI	6	NST	30	76.18	14.93	Fones	32	71.99	14.92	0.28	0.26	-0.22	0.78	0.278
			NST	30	76.18	14.93	Bass	30	70.56	16.90	0.35	0.26	-0.16	0.86	0.181
			Fones	32	71.99	14.92	Bass	30	70.56	16.90	0.09	0.25	-0.41	0.59	0.727
Deinzer et al. 2016; crowned teeth [48]	MPI	6	NST	30	42.18	25.56	Fones	32	36.78	23.99	0.22	0.25	-0.28	0.71	0.398
			NST	30	42.18	25.56	Bass	30	39.24	23.99	0.12	0.26	-0.39	0.62	0.651
			Fones	32	36.78	23.99	Bass	30	39.24	23.41	-0.10	0.25	-0.60	0.40	0.687
Harnacke et al. 2012 [49]	MPI	6	NST	19	62.51	20.05	Fones	18	50.19	15.33	0.67	0.34	0.01	1.34	0.047
			NST	19	62.51	20.05	Bass	18	64.72	19.31	-0.11	0.33	-0.75	0.54	0.739
			Fones	18	50.19	15.33	Bass	18	64.72	19.31	-0.81	0.35	-1.49	-0.13	0.019
Harnacke et al. 2016 [50]	MPI	6	NST	22	77.45	12.08	Fones	23	70.67	14.49	0.50	0.30	-0.10	1.09	0.100
			NST	22	77.45	12.08	Bass	23	76.05	17.26	0.09	0.30	-0.49	0.68	0.758
			Fones	23	70.67	14.49	Bass	23	76.05	17.26	-0.33	0.30	-0.91	0.25	0.264
Schlueter et al. 2013 [57]	TQHI	2	NST	27	1.80	0.47	Bass^1^	24	1.58	0.58	0.41	0.28	-0.14	0.97	0.145
			NST	27	1.80	0.47	Bass^2^	26	1.64	0.58	0.30	0.28	-0.24	0.84	0.279

*number of participants analyzed (for total n and drop outs see S1 Appendix); SD: Standard deviation; SMD: standardized mean difference [41]; CI (95%) lb: lower border of the 95% confidence interval of SMD [41]; CI (95%) ub: upper border of the 95% confidence interval of SMD [41]; p: probability value of corresponding SMD; NST: no specific technique; MPI: Marginal Plaque Index; TQHI: Turesky’s modification of the Quigley and Hein Index; PI: Plaque index;

^+^: # refers to the school where the study was conducted;. Means and SDs of Harnacke et al., 2012, 2016 and Deinzer et al. 2016 were obtained from the authors.

^1^instruction via leaflet;

^2^instruction via demonstration; Harnacke et al., 2012, 2016 and Deinzer et al. also assessed the TQHI that was not part of the NMA according to the study protocol specifications; these data are available in the S5 Appendix.

#### Supplementary searches during the data extraction and evaluation process

Supplementary searches from January 2022 to November 2023 revealed 120 titles independently screened by two reviewers (RD and ZE). They identified one potentially eligible study [62] and contacted the authors to prove that it met the criteria for measuring plaque after brushing. However, the authors did not respond. Therefore, this study was not considered for further analysis.

### Characteristics of the included studies

S1 Appendix provides a detailed overview of the characteristics of each study included. Table 3 summarizes the study design, outcomes, blinding of the examiners, age of the participants, control conditions, and brushing techniques. Most studies were parallel-arm, examiner-blinded, and involved young adults. The most often examined brushing technique was the Bass or the modified Bass technique (see Table 3).

When looking more closely at the description of the toothbrushing techniques investigated in the included studies, differences within the techniques became obvious. For example, four German studies referred to the Fones technique but instructed circular movements on the buccal/labial and palatinal/lingual surfaces even though the original description recommends scrubbing movements for the palatinal/lingual surfaces [48–50, 58]. One study referred to Bass [6] but described that the bristles should be “angled slightly towards” the gingival crevice (and not in a 45° angle as in the original): It also added further details that are not described in the original like “half of the bristles on the gingiva and half on the tooth”, and a “sweep” that follows an “intrasulcular circular stroke” [51 and personal communication, October 29th, 2023]. Another study [57] referred to the modified Bass technique [7] but instructed the participants to perform 3–5 jiggling and wiping cycles per area [57 and personal communication, December 13th, 2022] instead of remaining for 10–15 seconds in that area as described in the original. One study [55] cited another study as reference for the Roll technique. However, this study did not describe it [63] but referred to another reference [64] that described several techniques but did not name any of them as the Roll technique. Other studies neither referred to a publication regarding the applied technique nor described the investigated technique in detail [52, 53, 59], or referred to a reference, but did not explain it [55, 60]. Hence, in these studies it remained uncertain whether the technique was instructed according to its original description.

Blinding the participants to the brushing technique was impossible according to the type of intervention. Deinzer et al. and the two Harnacke et al. reports [48–50] state within the publication that participants were blinded to the research hypotheses.

Tables 4, 6, and 8 show the results of the included studies and further details regarding the time of the outcome assessment and the assessed indices.

Four of the nine studies assessed a plaque index that focused on the gingival margin, that is, the Plaque Index [PI; 65] and the Marginal Plaque Index [MPI; 66]. Three studies applied an index that assigns plaque distant from the gingival margin at least equal weight to that situated at the gingival margin: Turesky’s modification of the Quigley and Hein Index [TQHI; 67], the modified navy plaque index [MNPI; 68], and the Modified Benson Proximal Marginal Index [MBPMI; 69]. Sarvia et al. [56] did not describe the plaque index used. Two studies assessed both the MPI and TQHI [48, 49].

### Results of risk of bias analyses

**Fig 2:**
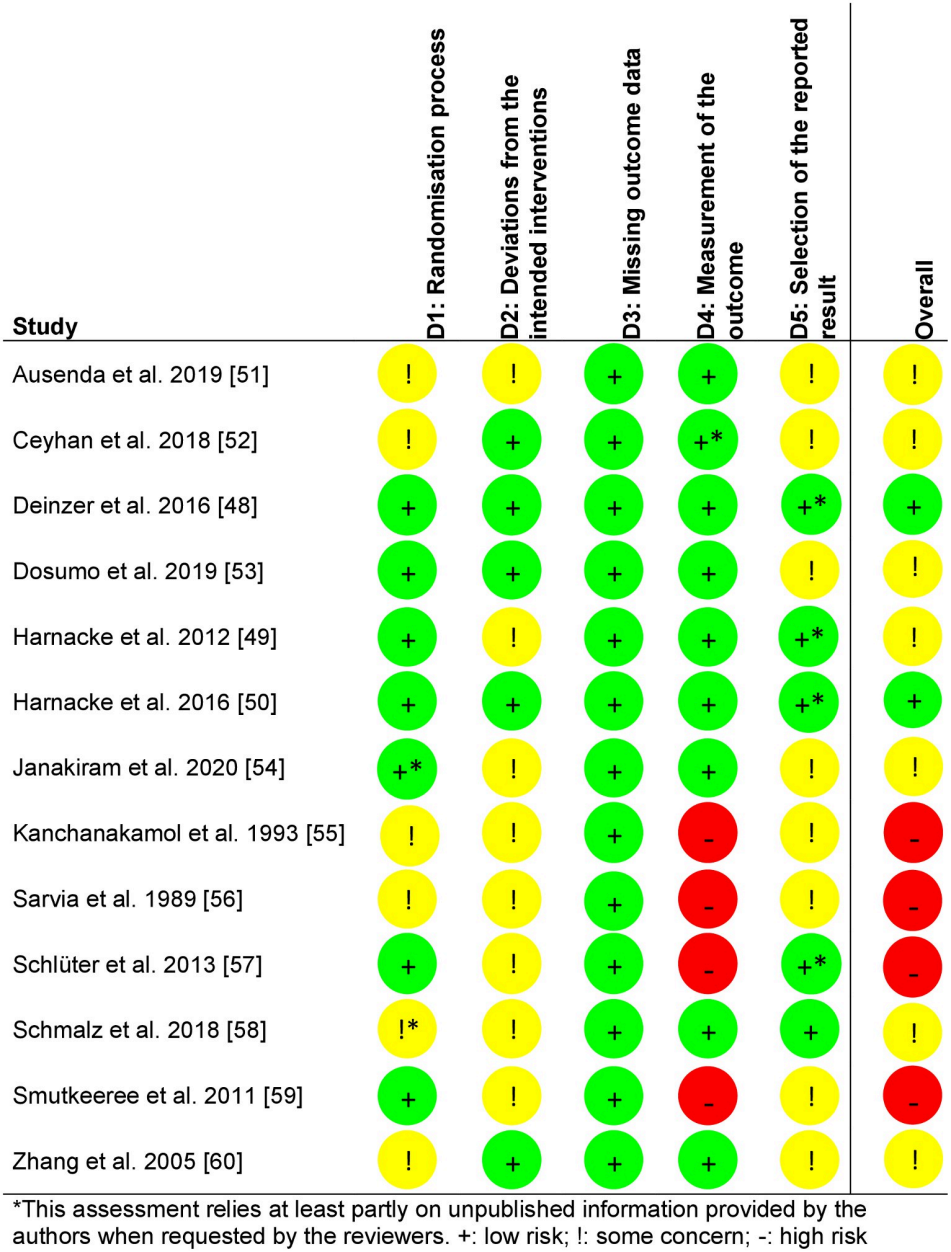
Results of the Risk of Bias (RoB) assessment with RoB2 [47].

Fig 2 shows the results of the RoB assessment. Most studies raised at least some concerns because relevant information was missing from the publication and attempts to obtain this information from the authors were unsuccessful. However, no study raised concerns regarding missing outcome data.

### Results of the assessment of indirectness on the study level

**Fig 3:**
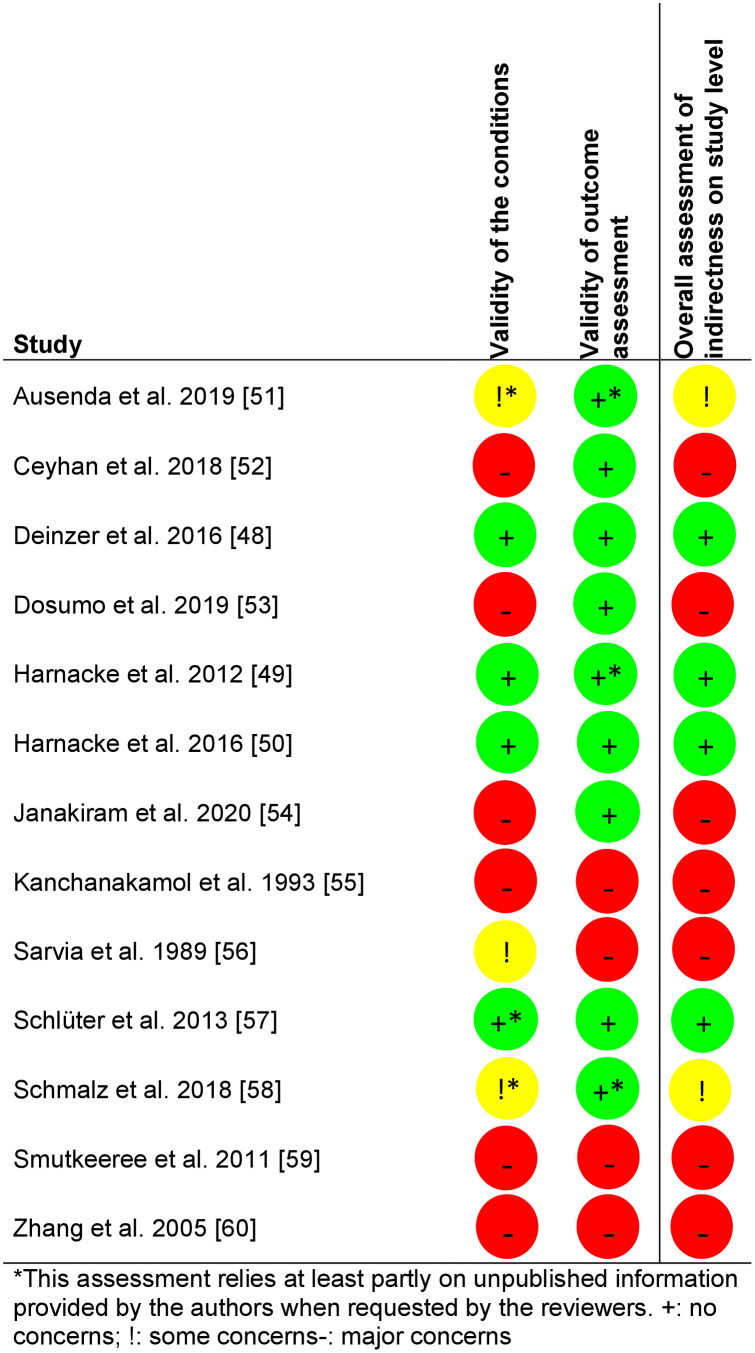
Results of the analyses regarding indirectness on the study level.

Fig 3 shows the results of the indirectness assessments at the study level [33]. According to the selection criteria, indirectness at the study level could not occur regarding the population but only regarding the validity of the intervention or outcomes. Most studies raised at least some concerns in this respect.

### Network meta-analysis

Tables 4, 6, and 8 contain all relevant comparisons and their statistics which are potentially eligible for NMA. S3 Appendix contains the data included into the NMAs. S4 Appendix contains all the outputs of the CINeMA tool [37, 38], including the network estimates of effect sizes, the ratings of concerns raised by the RoB, indirectness, imprecision, heterogeneity, and incoherence, and the resulting overall rating of confidence in the evidence.

Two studies included in the NMA assessed the respective outcomes only once, whereas all other assessed them more than once [52, 54]. To reduce heterogeneity in the study designs of the respective NMAs the outcomes of these studies were treated as secondary outcome dates because the time of their assessment was much closer to the first than to the last assessment date in other studies (see Tables 4 and 6).

#### Plaque after toothbrushing (primary outcome parameter)

Seven studies provided data for NMA of intervention effects on plaque [48–50, 57]. Four studies assessed plaque more than once after the intervention. According to the study protocol, the last assessment after the intervention was analyzed as primary outcome date and the assessment closest to the intervention as the secondary outcome date. A total of 293 (primary) and 370 (secondary) cases, with 14 (primary) and 17 (secondary) comparisons provided data for the NMA (see Table 4). Of these, three studies from one report that provided only eligible data for the secondary outcome [52] examined children and all others adults. Therefore, two NMAs were performed for the secondary outcomes. One NMA included all comparisons, and one included only comparisons from trials with adults. This was done to approximate the secondary objective of this review, which was to assess whether the effect of self-administered manual toothbrushing techniques on plaque and gingivitis differed between children, adolescents, and adults. Additional analyses that could have been used to address the other secondary aim of the study (differences in results according to the type of plaque index used) were not feasible because the type of plaque index was confounded with study group and participant age.

**Fig 4:**
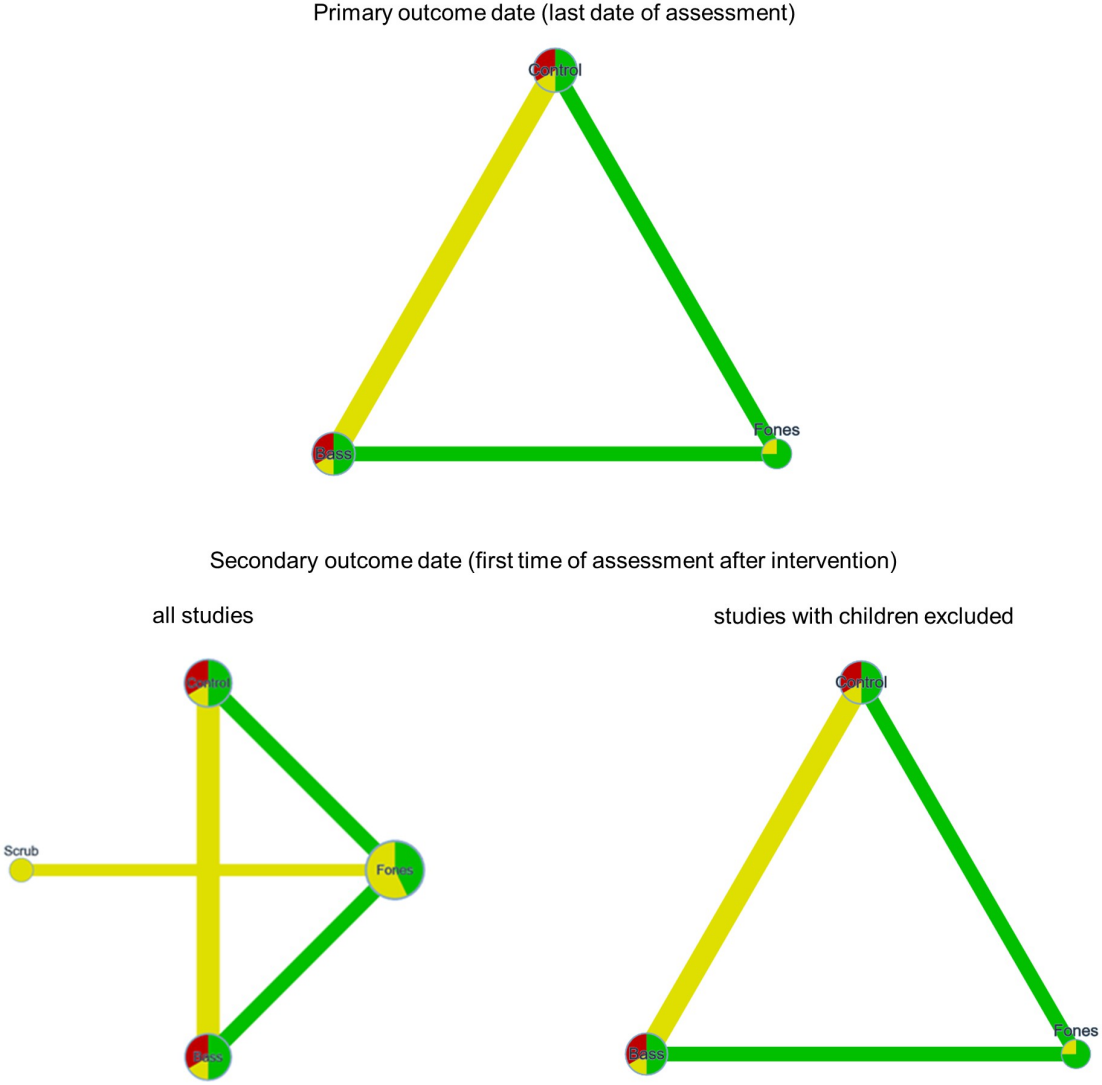
Network graphs of the comparisons of techniques for plaque assessed after brushing at the last date of assessment after the intervention (primary outcome date) and the first date of assessment after the intervention (secondary outcome date). The node size and the edge width represent the sample size, the node color and the edge color represent the RoB and the average RoB, respectively.

Fig 4 shows the network graphs of the available comparisons regarding plaque for the NMAs that referred to all studies and for the one that only referred to studies with adults.

**Fig 5:**
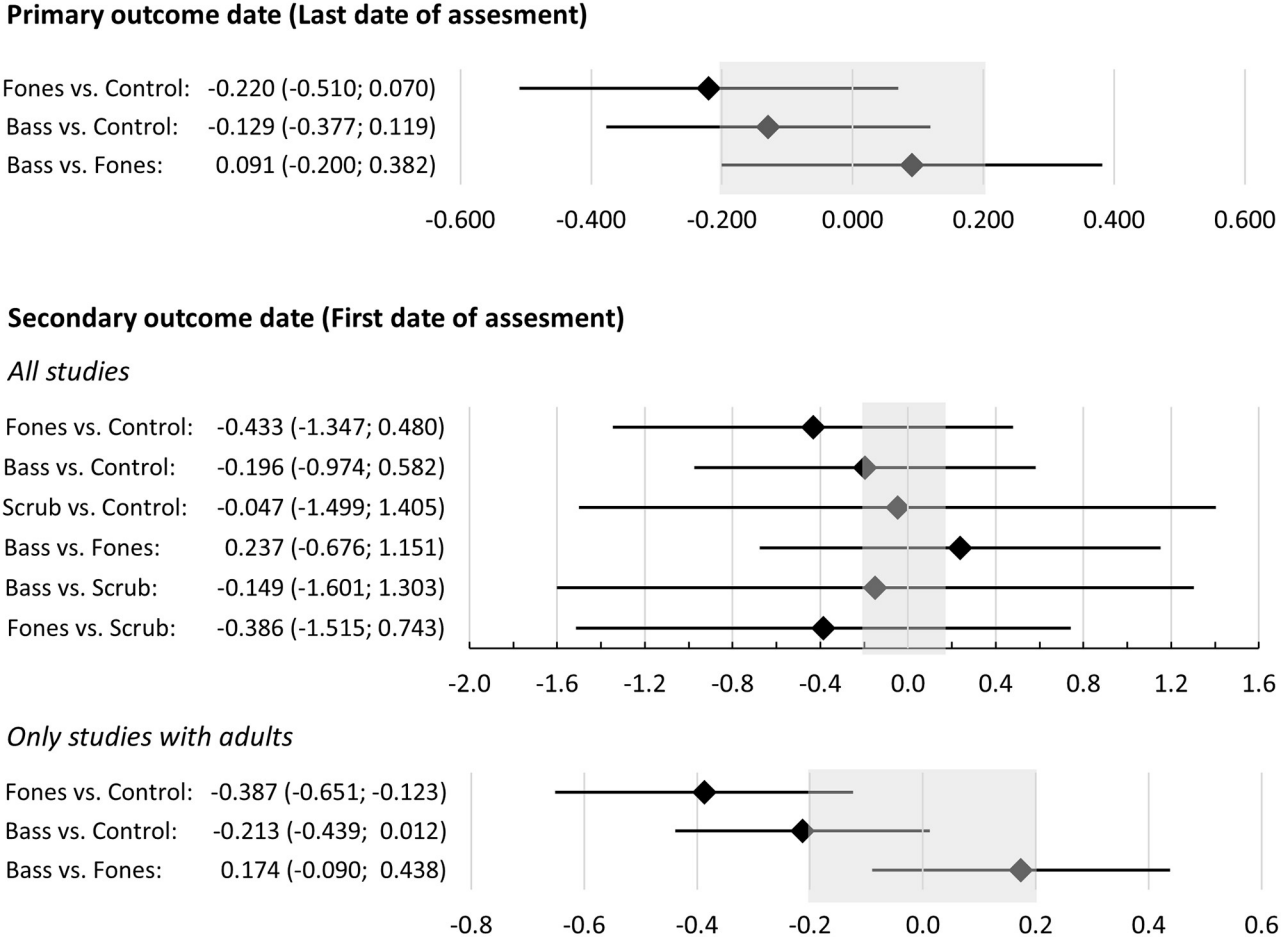
Network estimates of the standardized mean differences and the respective 95% confidence intervals of the comparisons of techniques for plaque assessed after brushing at the last date of assessment after the intervention (primary outcome date) and the first date of assessment after the intervention (secondary outcome date). Values outside the shaded area represent effects considered to be clinically important (SMD>0.2).

For the primary outcome date, the average RoB only raised concerns regarding the Bass vs. Control comparison. Indirectness raised no concerns. Fig 5 shows the network estimates for the respective comparisons. The imprecision of the estimates raised major concerns in the case of the Bass vs. Fones comparison, whereas the other two comparisons raised some concerns. There were some concerns regarding heterogeneity in the studies providing data for the Bass vs. Control and Fones vs. Control comparison, while there were no concerns in this respect regarding the Bass vs. Fones comparison. No comparison raised concerns regarding incoherence; the global test based on a random effects design-by treatment interaction model was not significant (p = 0.156; Chi^2^(1) = 2.010) as were the p-values of the inconsistency measures for the two estimates (Bass vs. Fones and Fones vs. Control) based on both direct and indirect comparisons (p = 0.174 and p = 0.212).

For the secondary outcome date, the average RoB raised no concerns for the Bass vs. Fones and Fones vs. Control conditions while all others raised some concerns. All comparisons that included Scrub raised major concerns in terms of indirectness whereas the remaining comparisons did not raise concerns in this regard. The results of the NMA varied considerably depending on whether the three studies with children were included (see Fig 5). When all studies were included, the imprecision raised major concerns for all comparisons, whereas no concerns emerged regarding heterogeneity (all p > 0.742). If only studies examining adults were included, the Fones vs. Control comparison raised no concerns regarding imprecision, whereas the remaining two comparisons raised some concerns. None of the three comparisons raised concerns regarding heterogeneity or incoherence (all p>0.380).

**Fig 6:**
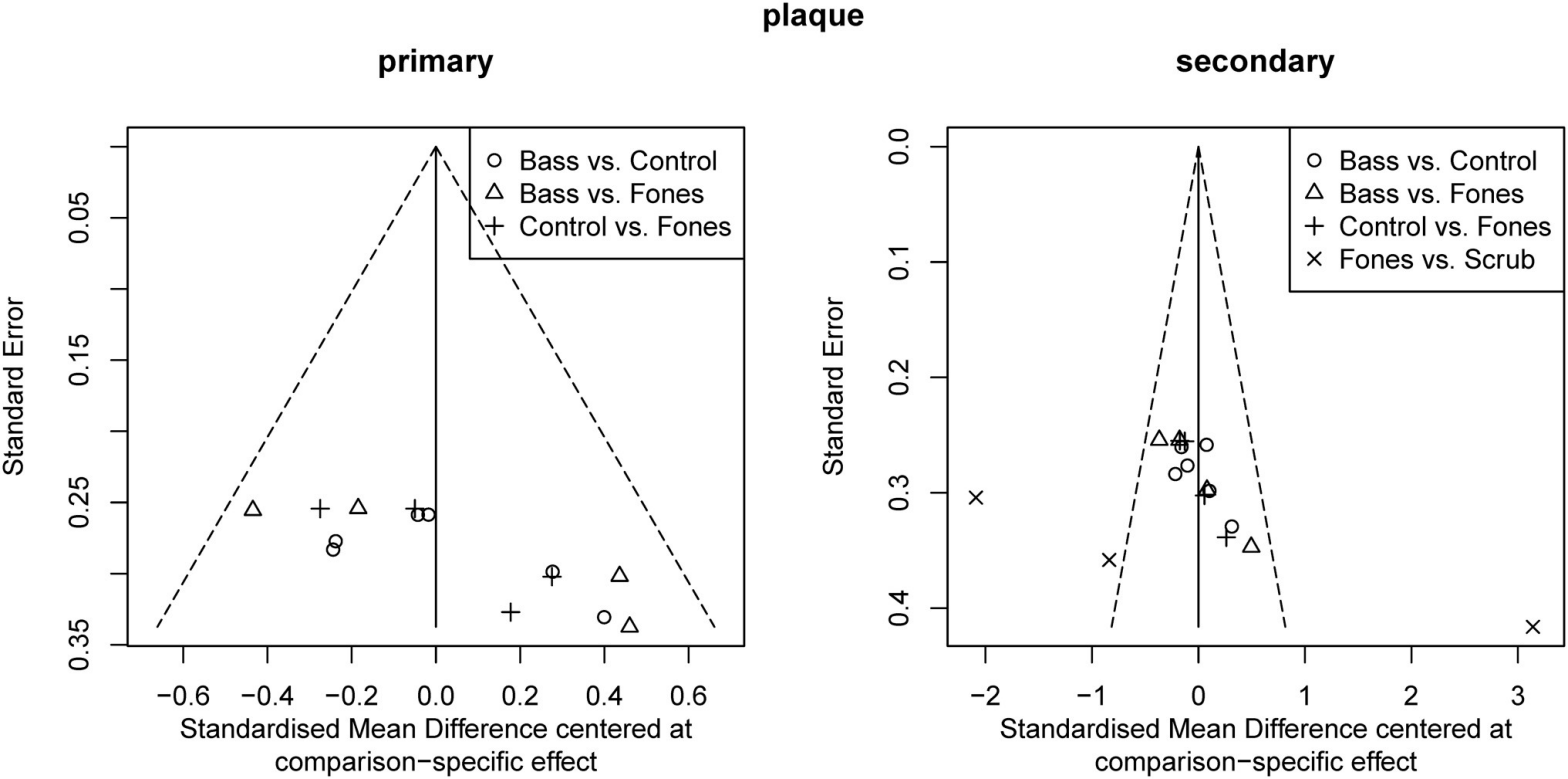
Funnel plots of the effect sizes of the comparisons regarding plaque after toothbrushing at the last assessment after intervention (primary outcome date) and the first one (secondary outcome date).

Fig 6 shows funnel plots of the data regarding plaque. The reported effect sizes showed a suspicious pattern only for the Fones vs. Scrub comparisons. However, all Fones vs. Scrub comparisons were based on a single publication. This publication reported conflicting results. This argues against a reporting bias due to the non-reporting of unexpected results. Nonetheless, the Scrub vs. Control comparison was considered high risk. This is because the literature search identified one study [56] that met the inclusion criteria for this systematic review, but the reviewers’ attempts to obtain the necessary data for inclusion in the NMA were unsuccessful.

Table 5 provides a comprehensive overview of the results of the NMA and the rating of confidence in the evidence, along with the reasons why the confidence level was downgraded. For the primary outcome date, the highest rating of confidence in the evidence was moderate (Fones vs. Control); for the secondary outcome date, high (Fones vs. Control) and moderate (Bass vs. Fones) confidence were achieved only when the analyses were restricted to adult-only studies.

**Table 5:** Overview of the results of the NMA for plaque after toothbrushing.

Comparison	network estimate	Confidence rating	Reasons for downgrading						informative statement to communicate the results [46]
			within study bias	reporting bias	indirectness	imprecision	heterogeneity	incoherence	
primary outcome date (last date of assessment after intervention; only studies with adults available)									
Fones vs. Control	-0.220	moderate				x	x		Fones probably results in a slight reduction
Bass vs. Control	-0.129	low	x			x	x		Bass may result in little to no difference
Bass vs. Fones	0.091	low				x			Bass may result in little to no difference
secondary outcome date (first date of assessment after intervention) —all studies included									
Fones vs. Control	-0.433	low				x			Fones may result in a slight reduction
Bass vs. Control	-0.196	very low	x			x			The evidence is very uncertain that Bass may result in little to no difference
Scrub vs. Control	-0.047	very low	x	x	x	x			The evidence is very uncertain that Scrub may result in little to no difference
Bass vs. Fones	0.237	low				x			Bass may result in a slight increase
Bass vs. Scrub	-0.149	very low	x		x	x			The evidence is very uncertain that Bass may result in little to no difference
Fones vs. Scrub	-0.386	very low	x		x	x			The evidence is very uncertain that Fones may result in a slight reduction
secondary outcome date (first date of assessment after intervention)—only studies with adults									
Fones vs. Control	-0.387	high							Fones results in a slight reduction
Bass vs. Control	-0.213	low	x			x			Bass may result in a slight reduction
Bass vs. Fones	0.174	moderate				x			Bass probably results in no difference

### Gingivitis (secondary outcome parameter)

Five of the included studies assessed gingivitis [49, 50, 54, 58, 59], of which one [54] assessed it only once and thus only provided data for the secondary outcome date. This resulted in 8 comparisons for the primary outcome date, with a total of 226 cases and 11 comparisons for the secondary outcome with a total of 346 cases (see Table 6).

**Table 6:** Comparisons available for NMA regarding the secondary outcome parameter: Gingivitis.

Outcome date/Study	index	weeks after intervention	1st study arm compared				2nd study arm compared				standardized mean difference				
			Intervention	n*	mean	SD	Intervention	n*	mean	SD	SMD	SE	CI (95%) lb	CI (95%) ub	p
**Primary outcome date (Last date of assessment)**															
Harnacke et al. 2012 [49]	PBI	28	NST	19	26.00	15.03	Fones	19	15.35	12.57	0.75	0.34	0.09	1.41	0.025
			NST	19	26.00	15.03	Bass	18	27.96	21.29	-0.10	0.33	-0.75	0.54	0.751
			Fones	18	15.35	12.57	Bass	18	27.96	21.29	-0.71	0.34	-1.38	-0.03	0.040
Harnacke et al. 2016 [50]	BOP	28	NST	22	7.36	4.70	Fones	23	10.50	7.39	-0.50	0.30	-1.09	0.10	0.102
			NST	22	7.36	4.70	Bass	24	10.84	7.01	-0.57	0.30	-1.16	0.02	0.059
			Fones	23	10.50	7.39	Bass	24	10.84	7.01	-0.05	0.29	-0.62	0.53	0.874
Schmalz et al. [58]	GI	12	NST	22	1.00	0.08	Fones	22	0.97	0.14	0.26	0.30	-0.34	0.85	0.393
Smutkeeree et al. 2011 [59]	GI	26	Scrub	29	2.42	0.28	Bass	28	2.43	0.34	-0.03	0.26	-0.55	0.49	0.905
**Secondary outcome date (First date of assessment)**															
Harnacke et al. 2012 [49]	PBI	6	NST	19	17.58	14.74	Fones	18	22.23	25.56	-0.22	0.33	-0.87	0.43	0.506
			NST	19	17.58	14.74	Bass	19	18.57	12.67	-0.07	0.32	-0.71	0.57	0.828
			Fones	18	22.23	25.56	Bass	19	18.57	12.67	0.18	0.33	-0.47	0.83	0.587
Harnacke et al. 2016 [41, 50]	BOP	6	NST	22	11.79	6.65	Fones	23	10.50	6.91	0.19	0.30	-0.40	0.77	0.532
			NST	22	11.79	6.65	Bass	24	11.15	8.40	0.08	0.30	-0.50	0.66	0.780
			Fones	23	10.50	6.91	Bass	24	11.15	8.40	-0.08	0.29	-0.66	0.49	0.776
Janakiram et al. [54]	GI	4	NST	40	0.90	0.61	Bass	40	0.90	0.40	0.00	0.22	-0.44	0.44	1.000
			NST	40	0.90	0.61	Fones	40	1.10	0.50	-0.36	0.23	-0.80	0.09	0.115
			Fones	40	1.10	0.50	Bass	40	0.90	0.40	0.44	0.23	-0.01	0.88	0.053
Schmalz et al. 2018 [58]	GI	4	NST	22	1.03	0.11	Fones	22	0.96	0.11	0.62	0.31	0.02	1.23	0.043
Smutkeeree et al. 2011 [59]	GI	4	Scrub	29	2.35	0.18	Bass	28	2.40	0.27	-0.22	0.27	-0.74	0.31	0.417

*number of participants analyzed (for total n and drop outs see S1 Appendix); SD: Standard deviation; SMD: standardized mean difference[41]; CI (95%) lb: lower border of the 95% confidence interval of SMD [41]; CI (95%) ub: upper border of the 95% confidence interval of SMD [41]; p: probability value of corresponding SMD; NST: no specific technique; PBI: Papillary Bleeding Index; BOP: Bleeding on Probing; GI: Gingival index. Means and SDs of Harnacke et al., 2012, 2016 were obtained from the authors. Schmalz et al. also assessed the PBI that was not part of the NMA according to the study protocol specifications; these data are available in the S5 Appendix.

**Fig 7:**
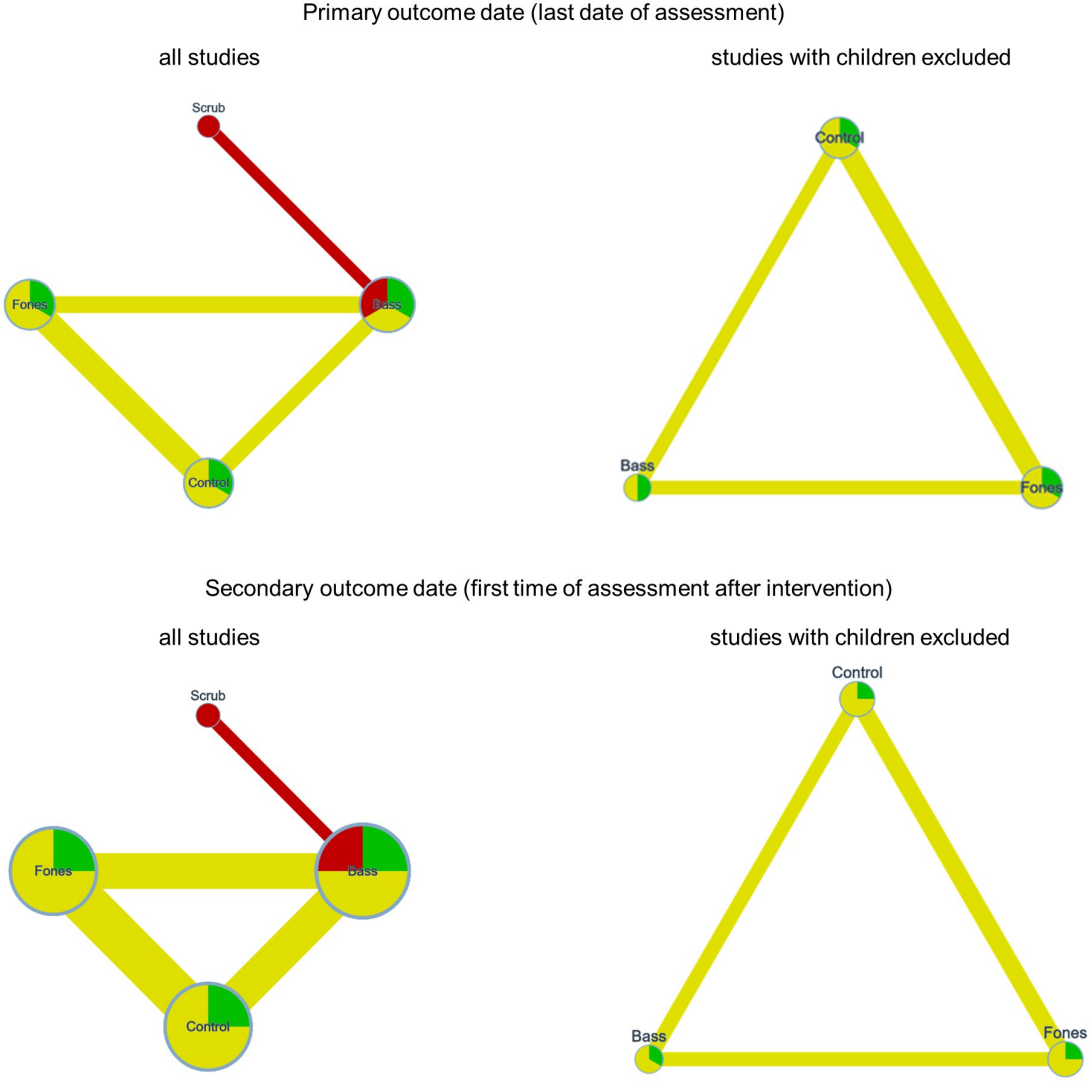
Network graphs of the comparisons of techniques for gingivitis at the last date of assessment after the intervention (primary outcome date) and the first date of assessment after the intervention (secondary outcome date). The node size and the edge width represent the sample size, the node color and the edge color represent the RoB and the average RoB, respectively.

One study [59] examined children, and all others studies examined adults. Therefore, two NMAs were performed for the primary outcome date and two for the secondary outcome date. One NMA included all comparisons, the second included only comparisons from trials with adults. Fig 7 shows the network graphs.

As can be seen from the network graphs, the only study with children [59] added three comparisons regarding scrub but did not affect the Bass vs. Control, Fones vs. Control, and Fones vs. Bass comparisons. Accordingly, the following presentation of the results refers to all studies; however, one should keep in mind that the only comparisons that contained data of children were those including the Scrub technique.

**Fig 8:**
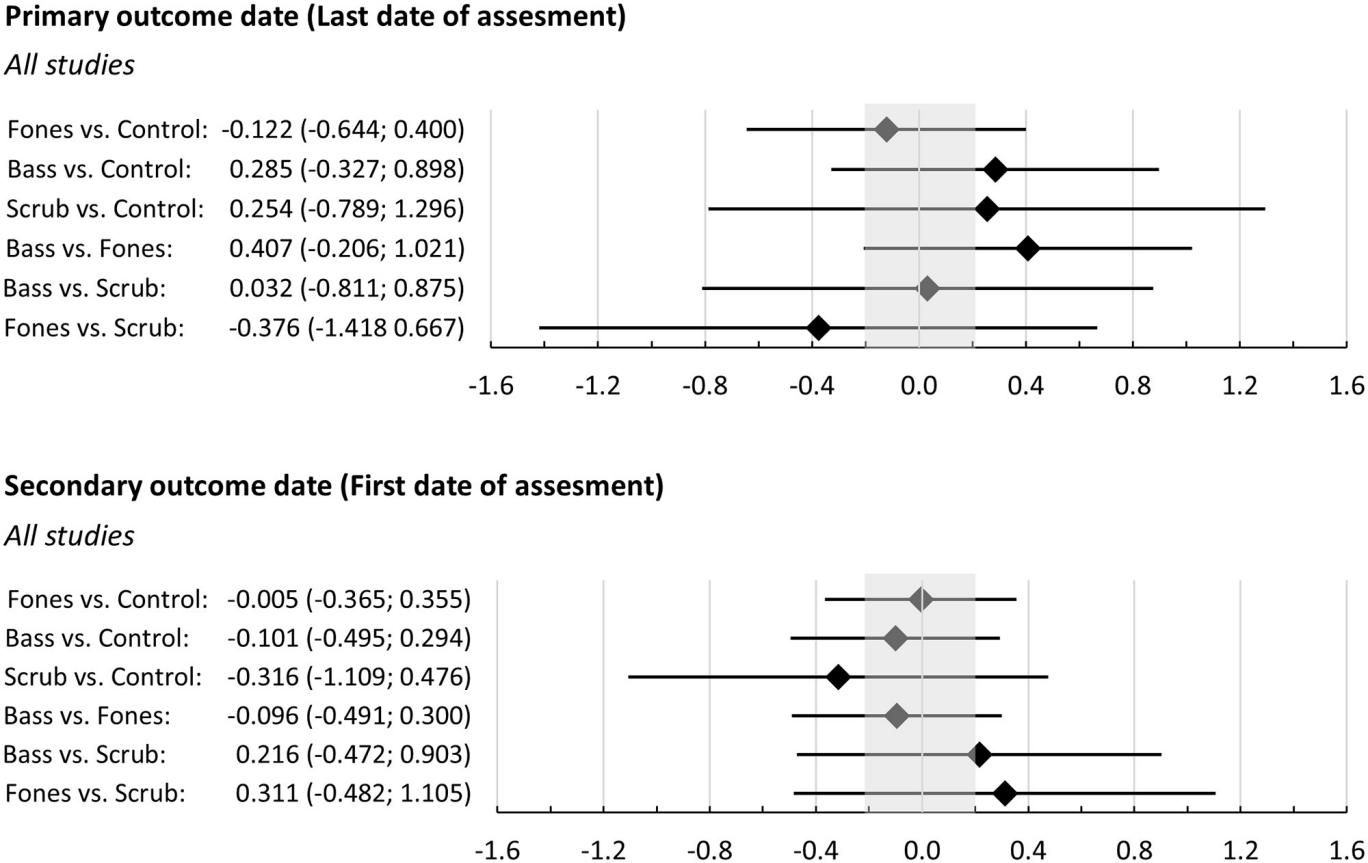
Network estimates of the standardized mean differences and the respective 95% confidence intervals of the comparisons of techniques for gingivitis assessed after brushing at the last date of assessment after the intervention (primary outcome date) and the first date of assessment after the intervention (secondary outcome date). Values outside the shaded area represent effects considered to be clinically important (SMD>0.2).

Regarding the primary outcome date the average RoB raised no concerns for the Bass vs. Fones comparison and major concerns for the Bass vs. Scrub comparison. All other comparisons raised some concerns. The average indirectness raised major concerns for all comparisons regarding the Scrub technique and no concerns for the remaining comparisons. None of the comparisons raised concerns regarding heterogeneity or incoherence (all p>0.647), but all raised major concerns regarding imprecision (see Fig 8 with the respective network estimates).

Regarding the secondary outcome date, the average RoB raised major concerns regarding the Bass vs. Scrub comparison and some concerns regarding all other comparisons. The average indirectness raised major concerns for all comparisons including the Scrub technique and some concerns regarding the Fones vs. Control comparison. The heterogeneity raised no concerns. The global test of incoherence based on a random-effects design-by-treatment interaction model revealed a significant result (Chi^2^(1) = 5.608; p = 0.018). This resulted in major concerns for all comparisons including the Scrub technique and for the Fones vs. Control comparison. The incoherence rating for the Bass vs. Control comparisons, which is based on mixed evidence raised some concerns (p = 0.073).

**Fig 9:**
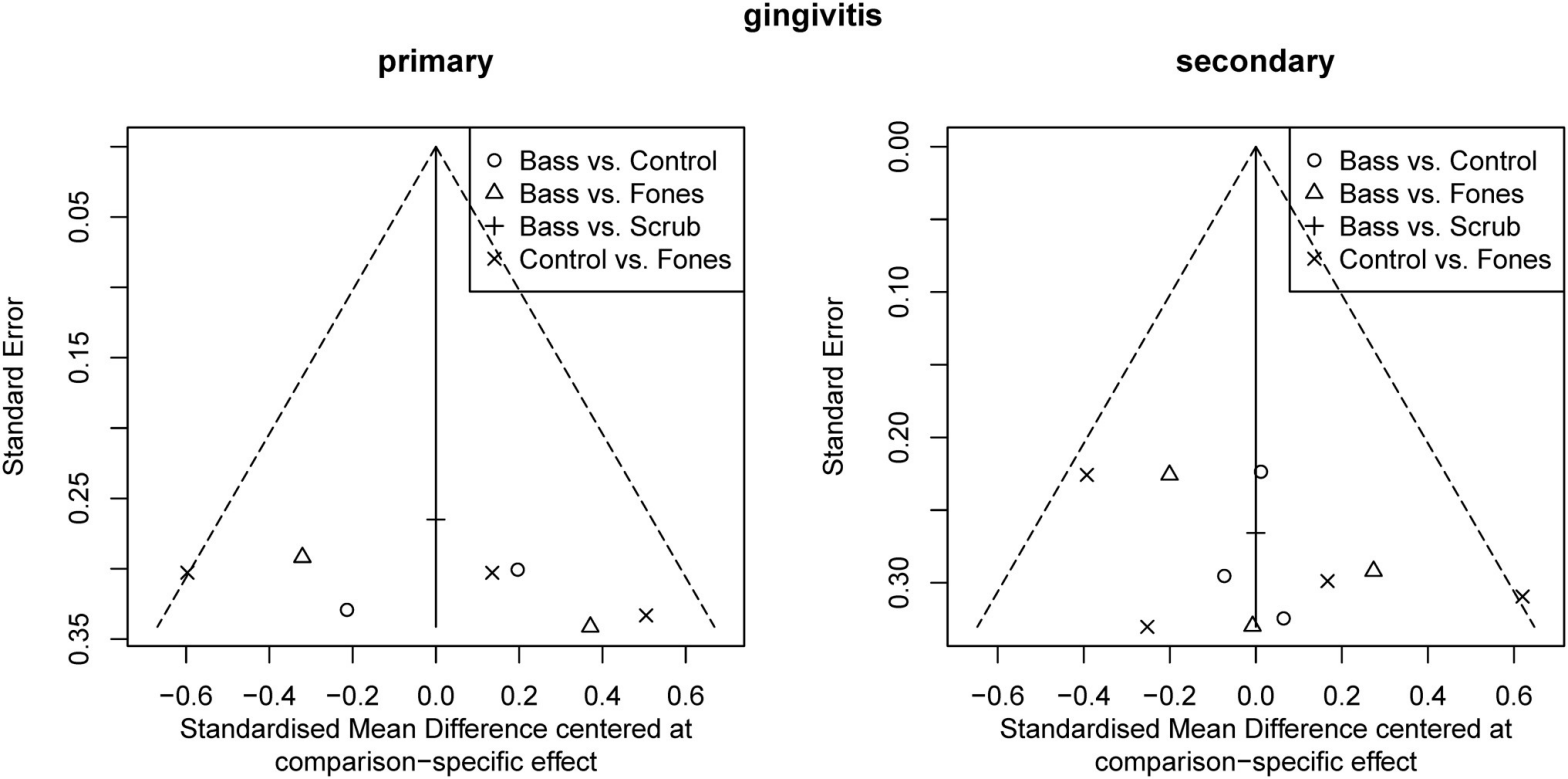
Funnel plots of the effect sizes of the comparisons regarding gingivitis at the last assessment after intervention (primary outcome date) and the first one (secondary outcome date).

Fig 9 shows the Funnel plots for the data regarding plaque. They raised no concerns. However, the Bass vs. control comparison was considered high risk because the literature search identified two studies [51, 53] that met the inclusion criteria for this systematic review, but the reviewers’ attempts to obtain the necessary data for inclusion in the NMA were unsuccessful.

Table 7 provides a comprehensive overview of the results of the NMA and the rating of confidence in the evidence, along with the reasons why the confidence level was downgraded. Confidence in the evidence was very low for all comparisons, except for the Bass vs. Fones comparison regarding the primate outcome date.

**Table 7:** Overview of the results of the NMA for gingivitis.

Comparison	network estimate	Confidence rating	Reasons for downgrading						informative statement to communicate the results [46]
			within study bias	reporting bias	indirectness	imprecision	heterogeneity	incoherence	
primary outcome date (first date of assessment after intervention)									
Fones vs. Control	-0.122	very low	x			x			The evidence is very uncertain that Fones may have little to no effect
Bass vs. Control	0.285	very low	x	x		x			The evidence is very uncertain that Bass may result in a slight increase
Scrub vs. Control	0.254	very low	x		x	x			The evidence is very uncertain that Scrub may result in a slight increase
Bass vs. Fones	0.407	low				x			Bass may result in a slight increase
Bass vs. Scrub	0.032	very low	x		x	x			The evidence is very uncertain that Bass may have little to no effect
Fones vs. Scrub	-0.376	very low	x		x	x			The evidence is very uncertain that Fones may result in a slight decrease
secondary outcome date (first date of assessment after intervention)									
Fones vs. Control	-0.005	very low	x		x	x		x	The evidence is very uncertain that Fones may have little to no effect
Bass vs. Control	-0.101	very low	x	x		x		x	The evidence is very uncertain that Bass may have little to no effect
Scrub vs. Control	-0.316	very low	x		x	x		x	The evidence is very uncertain that Scrub may result in a slight reduction
Bass vs. Fones	-0.096	very low	x			x			The evidence is very uncertain that Bass may have little to no effect
Bass vs. Scrub	0.216	very low	x		x	x		x	The evidence is very uncertain that Bass may result in a slight increase
Fones vs. Scrub	0.311	very low	x		x	x		x	The evidence is very uncertain that Fones may result in a slight increase

### Results of the studies not included in the network meta-analyses

Table 8 provides an overview over the results of the studies that were not included in the NMA. Five studies with six comparisons provided insufficient data to estimate the effect sizes of plaque after brushing or gingivitis. All studies raised at least some concerns regarding the RoB, and all but one [51] raised major concerns regarding indirectness (see Figs 2 and 3).

**Table 8:** Results of studies not available for NMA due to unavailable statistics or heterogeneity.

Study	index	weeks after intervention	1st study arm compared				2nd study arm compared				standardized mean difference				
			Intervention	n*	mean	SD	Intervention	n*	mean	SD	SMD	SE	CI (95%) lb	CI (95%) ub	p
Sarvia et al. with incentive [48]	n.i.	1	NST	14	0.54	n.i.	Scrub	18	0.50	n.i.	not calculable				
Sarvia et al. without incentive [48]	n.i.	1	NST	17	0.56	n.i.	Scrub	15	0.38	n.i.	not calculable				
			**Decline (n.i. on absolute values)**				**Decline (n.i. on absolute values)**								
Zhang et al. [52]	MBPMI	3	Vertical	20	68	32	Bass	20	84	27	not calculable				
Kanchanakamol et al. [47]	MNPI	2	Roll	46	1.09	n.i.	Bass	46	1.4	n.i.	not calculable				
Dosumu et al. [45]	BOP	4	NST	25	6.14	n.i.	Bass	25	5.71	n.i.	not calculable				
		1	NST	25	4.93	n.i.	Bass	26	4.03	n.i.	not calculable				
			**number of sites (n.i. on individuals):**				**number of sites (n.i. on individuals):**								
				**total**	**affected**			**total**	**affected**						
Ausenda et al. [43]	GI	12	NST	1320	578		Bass	1140	132		not calculable				
		4	NST	1320	415		Bass	1196	148		not calculable				

*number of participants analyzed (for total n and drop outs see S1 Appendix); SD: Standard deviation; SMD: standardized mean difference[41]; CI (95%) lb: lower border of the 95% confidence interval of SMD [41]; CI (95%)ub: upper border of the 95% confidence interval of SMD [41]; p: probability value of corresponding SMD; NST: no specific technique; n.i.: no information available on request; MBPMI: Modified Benson Proximal Marginal Index; MNPI: Modified Navy Plaque Index; BOP: Bleeding on Probing; GI: Gingival Index

### Sensitivity analyses

Due to the small number of studies and study groups, meaningful sensitivity analyses were not possible.

## Discussion

Manual toothbrushes are the most commonly used oral hygiene device worldwide; however, there has yet not been a consensus on the effectiveness of different brushing techniques. Hence, this systematic review was conducted to synthesize evidence from RCTs regarding the effect of any specific compared to any other specific or no specific self-applied manual toothbrushing technique on plaque and gingivitis in humans of any age. The primary outcome parameter was plaque after toothbrushing. Gingivitis was the secondary outcome parameter. A total 13 papers comprising 15 studies were eligible, ten of which provided information on plaque after brushing and seven on gingivitis. Owing to missing data, only seven studies on plaque and five on gingivitis could be included in NMAs.

### Summary of main results

Due to the limited number of study groups and studies, only the main objective of this review could be addressed. This was to compare the effects of different self-applied manual toothbrushing techniques on plaque (primary outcome parameter) and gingivitis (secondary outcome parameter) in children and adults. The two secondary aims (difference between different age groups and differences between different plaque indices) could not be addressed, even though it was possible to restrict data analyses to studies with adults and describe whether this would lead to different results than analyzing all studies. The main results of these analyses are summarized as follows.

#### Plaque after toothbrushing (primary outcome parameter)

The NMA revealed evidence of moderate to high certainty (depending on the time of assessment) that adults trained the Fones technique will show slightly reduced plaque levels after brushing compared to controls. There was evidence of low certainty that adults taught the Bass technique would show a reduction in plaque after brushing in the short term and would result in little to no difference in the long term. Accordingly, the NMA also revealed evidence of low to moderate certainty that the two techniques might be equivalent in adults with regard to plaque after brushing. Regarding children, only one report of three studies that compared Fones vs. Scrub provided data for NMA. Adding this report to the NMA reduced the certainty of the evidence of comparisons observed in studies confined to adults, although it did not change the direction of results.

Studies that could not be included in the meta-analysis raised some concerns or even had a high RoB; all of them raised major concerns regarding indirectness (see Figs 2 and 3). Thus, they provided additional evidence of limited certainty. One analyzed young children and described an advantage of the Scrub technique vs. no specific technique [56]. Two other studies analyzed adults and reported an advantage of the Bass technique over vertical brushing [60] or the Roll technique [55].

If no other reasons argue for the techniques, the evidence summarized in this systematic review suggests that one may recommend training of the Fones technique to reduce plaque after toothbrushing; training of the Bass technique may also be recommended, although the certainty of the evidence is lower. These two techniques should be preferred to recommending the Scrub technique.

#### Gingivitis (secondary outcome parameter)

The NMA revealed evidence of low certainty that, in the long term, a training of the Bass technique may slightly increase gingivitis compared to a training of the Fones technique. Additionally, evidence of very low certainty pointed in the direction that it may also slightly increase gingivitis in the long term when compared to controls, and may be equivalent to a training of the Scrub technique. Regarding short-term effects, there was evidence of very low certainty that a training of the Bass technique may have little or no effect as compared to Fones or a control condition but may slightly increase gingivitis when compared to a training of the Scrub technique.

Regarding long term effects of training of the Fones technique, there was evidence of low certainty that it may reduce gingivitis when compared to Bass (see above), and of very low certainty that it may slightly reduce gingivitis when compared to Scrub. Evidence of very low certainty suggested that it may have little or no effect when compared to a control condition. Regarding short-term effects there was evidence of very low quality that a training of the Fones technique may be equivalent to a training of the Bass technique or a control condition while it may slightly increase gingivitis when compared to a training of the Scrub technique.

Accordingly, the evidence was very uncertain, that a training of the scrub technique may slightly increase gingivitis as compared to a control condition or Fones in the long term and slightly decrease it in the short term.

The two studies that could not be included in the NMA [51, 53] raised at least some concerns regarding RoB and indirectness and thus provided additional evidence of limited certainty. This evidence pointed into the direction of an advantage of the Bass technique over a control condition in both conditions.

Therefore, in the absence of other reasons to use either technique, the available evidence summarized in this systematic review suggests that training in either the bass or scrub technique should **not** be recommended for the long-term reduction of gingivitis.

### Heterogeneity of the studies included

The few studies that met the inclusion criteria for this review varied widely in several aspects. However, the limited number of studies eligible for NMA did not allow for sensitivity analyses. Still, these sources of heterogeneity should be considered when interpreting the results of this systematic review and NMA. Studies differed with regard to the study population they included, that is, children, visually impaired students, dental students, and adults with an age of up to 82 years. Most authors did not describe their instruction procedure. Where these data were available, detailed analyses often revealed that the instructions deviated from the original and were not standardized. This complicates the data analysis and interpretation.

The mode of instruction varied widely among the studies, ranging from oral instruction to providing computer presentations and hands-on training. Some studies varied the modes of instruction [52, 56, 57]. Although the most efficient mode of instruction is still unknown, it appears to be important and should be considered when interpreting results [14, 70].

Furthermore, the studies varied with regard to the indices used, which may have contributed to the heterogeneity of the results. Considering that marginal plaque leads to gingivitis [71], plaque removal next to the gingival margin is important for preventing gingivitis. Within a direct comparison, it also appeared that a marginal plaque index might respond more sensitively to changes in oral hygiene than one considering more coronal plaque [66]. Most studies applied plaque indices that focus on the gingival margin, while three used indices that gave plaque distant from the gingival margin at least an equal weight [55, 57, 60]. Similarly, studies varied with regard to the Gingivitis indices applied. Bleeding of probing, which was assessed in two studies [50, 53], is considered prone to false-positive results. This index is considered less sensitive to true changes in gingivitis than the indices applied in other studies that evaluated marginal probing [72]. An additional important source of heterogeneity in the results might be the overall duration of the study, which ranged from less than one to 28 weeks.

### Recommendations for future research

The available evidence included in this review was limited to only 13 studies providing data at all, and only 7 and 5 studies providing data for NMAs on effects of toothbrushing techniques on plaque and gingivitis, respectively. Therefore, whether the training of any brushing technique is beneficial requires further investigation.

This research should avoid flaws often observed when evaluating the RoB. In the present analysis, most studies raised at least some concerns. In most cases this was due to missing information in the manuscripts and no or insufficient responses of the authors when asked for this information. The only two studies that were considered a low RoB had some advantage in this regard because they were related to one of the authors of this review and information missing in the manuscript was easy to obtain [48, 50]. Thus, a more thorough reporting of the details of a study prior to its onset is urgently recommended. Furthermore, more than half of the studies did not apply the intervention in a standardized manner or provided insufficient information on that. Because studies that provided such information often differed in their intervention from the source they cited, it seems to be essential that the intervention be described in detail in every case. Additionally, most studies reported some calibration of the examiners, but only a few delineated details of the calibration procedure and criteria. However, this is also an importing piece of information on the validity of the outcome assessments.

A secondary objective of this review was to determine whether the effects of different self-applied manual toothbrushing techniques differ between children, adolescents, and adults. However, the small number of studies did not allow for such an analysis. The recommendation provided by professionals vary between children and adults [15]. For example, the Fones technique is often recommended for children, and the (modified) Bass technique is often recommended for adults. However, according to the evidence provided here, there is no firm evidence justifying such a differentiation. Furthermore, adults might carry forward the brushing behavior they learned as a child rather than implementing a new technique [73]. Video observation studies support this hypothesis [19, 23]. They showed that even when an oscillating-rotating powered toothbrush takes over the brushing motion, most people cannot resist adding their brushing motion [74, 75]. Such studies also demonstrate that, at least in the German population, most children and adults apply aspects of the Fones and Scrub technique and vertical brushing. However, few aspects are reminiscent of Bass or modified Bass technique [19, 20, 23, 24, 75, 76].

This brings about another important point that should be considered in future research: the participants might have varying degrees of familiarity with the various techniques taught in these studies. They may have already been practicing the specific techniques that the study was meant to teach them. Therefore, without information on the brushing techniques people used when entering the study, it is difficult to interpret the effect of teaching an additional technique. If the technique requires a change in the previous behavior, it is also important to choose a study duration that allows participants to train the new behavior. Furthermore, reinstruction might be necessary to avoid relapses of the old brushing pattern. Only two of the studies included in this review assessed brushing behavior before to the intervention video analysis (Schlueter et al.) [57] or a questionnaire (Deinzer et al.) [48] and only three instructed the technique repeatedly (Ausenda et al., Janakiram et al., Schlueter et al.) [51, 54, 57].

### Limitations of this review

The strengths of the current systematic review are that it analyzed data from multiple databases, that it included people of all ages, and that it made several attempts to obtain information that was not readily available in original publications. By synthesizing all eligible results within an NMA, it also allowed for a summary quantification of the effects observed in the available studies. In addition to these strengths, this systematic review and NMA has some limitations. The literature search may have missed publications that were not registered in the databases, even though it covered the databases that Cochrane considers as the most important sources [77]. This review was based on RCTs and ignored results from non-RCTs. This was done because Non-RCTs have a much higher risk of bias than RCTs. Some studies that assessed relevant data could not be included into NMAs because of unsuccessful attempts to obtain relevant statistical information from the authors. Additionally, only studies providing data on plaque after toothbrushing were considered eligible for analyses of the primary outcome parameter. Because toothbrushing aims to remove plaque completely, the effectiveness of a brushing technique can best be measured by analyzing the oral cleanliness achieved immediately after its application. Plaque levels measured without reference to toothbrushing are subject to several influences other than toothbrushing, such as time since last oral hygiene and food and drink consumption. Similarly, plaque removal (difference in values before and after brushing) is difficult to interpret, because the degree of removal depends on the baseline value. Gingivitis was assessed as the secondary outcome parameter. Gingiva responds sensitively to long-term changes in oral hygiene. Therefore, gingivitis can be used as an indicator of alterations in daily oral hygiene after instruction in a brushing technique. The review did not include data on patients with fixed orthodontic appliances because such appliances hamper oral hygiene and require specific measures. The literature search has focused on self-administered manual toothbrushing and ignored studies in which a third party administered a toothbrushing technique or studies that only analyzed toothbrushing with a powered toothbrush. This was done to provide meaningful data on the effectiveness of toothbrushing techniques applied at home.

### Comparison to previously published work

The current review extends the results of a recent review on the effectiveness of manual toothbrushing techniques [78] by performing a network meta-analysis and including studies on children, not only adults. In contrast to the previous review, it excludes studied in which the brushing technique was applied by persons other than the participants and non-randomized trials. It also included five studies that were published after the authors of the previous review completed their literature search in 2018 and two studies that they did not find with their search algorithm. Nonetheless, the essence of the results remains the same. The limited number of studies prevents more conclusive results. Likewise, deviations from the originally described techniques, different instruction modes, lack of standardization of the intervention, and pre-experiences of the participants might have flawed the conclusions regarding the effectiveness of a specific technique. Future studies should consider these factors.

### Conclusion

According to this analysis, the Fones or Bass techniques may be recommended to reduce plaque after toothbrushing in the long term. The Bass and the Scrub techniques should not be recommended to reduce gingivitis in the long term. However, the amount and confidence in the certainty of the available evidence supporting these recommendations is limited and further research is required. The current data-base does not allow analyses of whether the effect of self-applied manual toothbrushing techniques on plaque and gingivitis differs between children, adolescents and adults. The existing data-base also does not allow to determine whether the effects on plaque differ depending to the type of plaque index assessed.

## Supporting information

S1 Appendix Details of reports included in this review. Contains the details of each study included in the systematic review.
(PDF)

S2 Appendix List of reports excluded after full-text reading. Lists the reports excluded after full text reading and provides the reasons for exclusion. Also lists the reports that were not accessible.
(PDF)

S3 Appendix Data available for NMA. Contains the full data-set ready analysing using meta-analytic procedures.
(XLSX)

S4 Appendix CINeMA outputs. Contains all the outputs of CINeMA and the final evaluation of confidence in the evidence.
(PDF)

S5 Appendix Alternative plaque and gingivitis parameters. When a study assessed more than one plaque or gingivitis index, only one was included in the NMA and depicted in Tables 4 and 6. This appendix shows the effect sizes of the treatment comparisons regarding the alternative plaque or gingivitis index not shown in Tables 4 and 6.
(PDF)

S1 Checklist PRISMA NMA checklist of items to include when reporting a systematic review involving a network meta-analysis. (DOCX)

## Abbreviations

BOP: Bleeding on probing
CINAHL: Cumulative Index to Nursing and Allied Health Literature
CINeMA: Confidence in Network Meta-Analyses
GI: Gingival Index
GRADE: Grading of Recommendations Assessment, Development and Evaluation
ICC: intra class coefficient
ICTRP: WHO International Clinical Trials Registry Platform
MPI: Marginal Plaque Index
MPMPI: modified proximal marginal plaque index
NMA: Network meta-analysis
NST: No specific technique
OHA: Oral Hygiene Advice and Training
PI: Plaque Index
PICO: Population, Intervention, Control, Outcome
POD: Primary outcome date
RCT: Randomized controlled trial
RD: Renate Deinzer (author)
RoB: Risk of bias
SD: Standard deviation
SMD: Standardized Mean difference
SOD: Secondary outcome date
SS: Sonja Sälzer (author)
SSAMTT: Specific self-applied manual toothbrushing technique
TQHI: Turesky’s modification of the Quigley and Hein Index
UW: Ulrike Weik (author)
WHO: World health organization
ZE: Zdenka Eidenhardt (author)
