# Supplementary material for: Manual toothbrushing techniques for plaque removal and the prevention of gingivitis—A systematic review with network meta-analysis
Source: PLoS One. 2024 Jul 5;19(7):e0306302. doi: 10.1371/journal.pone.0306302 (PMC11226064; doi:10.1371/journal.pone.0306302)
Supplement: S1 Appendix — Contains the details of each study included in the systematic review. (PDF) [file pone.0306302.s001.pdf]

S1: Characteristics of the publications fulfilling the inclusion criteria and analysed in Network meta-analysis (NMA) and/or descriptively

Legend:

*: information obtained from the authors of the publication on request;
not available: parameter not assessed / no further dates of outcome assessment;
n.i.: no information available on request
n.r.: no restriction reported

**Ausenda et al., 2019**	Not included in NMA
Study design	**Arms**: 2 parallel<br>**Blinding**: Examiners blinded<br>**Standardized instruction of the technique**: n.i.<br>**Calibration of examiners**: yes*
Participants	**Sample size**: N=48<br>**Gender**: equally distributed (p=.25)<br>**Age**: ≥ 18 years<br>**Educational level**: n.r.<br>**Dental education**: n.r.<br>**General health status**: n.r.<br>**Smoking status**: non-smokers<br>**Dental status and/or periodontal status**: ≥ 20 teeth; cervical bleeding ≥ 20%; healthy cervical periodontal condition (PPD ≤ 3mm)
Interventions<br>(n included/completed)	**Control:** no specific technique (24/24)<br>**Intervention:** Bass technique (24/21)<br>**Professional tooth cleaning:** no<br>**Referral of the instruction to original publication of the technique**: refers to Bass (1954)<br>**Instruction describes the technique applied like the original**: no*
Provided devices	Toothbrush (Oral B Indiator Soft); toothpaste (Crest Pro Health)*
Provided instructions	<u>**All groups:**</u><br>Brushing twice/day 2 minutes<br><u>**Intervention group:**</u><br>Additional instruction with respect to brushing technique<br>**Mode**: written and oral<br>**Frequency**: 3x at baseline (T1); 4 weeks (T2) and 12 weeks (T3)<br>**Provider/qualification**: n.i.<br>**Instruction for application at home**: no
Outcome parameters<br><br><br>Outcome dates	**Primary (plaque after toothbrushing):** not available*<br>**Secondary (Gingivitis):** (modified) GI*<br><br>**Primary (last date of outcome assessment):** 12 weeks after intervention (T3)<br>**Secondary (first date of outcome assessment):** not applicable (instruction at each time point)<br>Other:4 weeks after intervention (T2)

**Ceyhan et al., 2018**	Not included in NMA
Study design	**Arms**: 2 parallel<br>**Blinding**: examiners blinded*<br>**Standardized instruction of the technique**: n.i.<br>**Calibration of examiners**: n.i.
Participants	**Sample size**: N=163<br>**Gender**: 78 female/ 85 male<br>**Age**: 5-6 years

	**Educational level**: preschool children<br>**Dental education**: no<br>**General health status**: no systemic disease; no mental or physical disabilities<br>**Smoking status**: not applicable<br>**Dental status and/or periodontal status**: no oral soft tissue pathology
Interventions<br>(n included/completed)	**1st preschool**<br>**Intervention 1**: horizontal scrubbing (17/17)<br>**Intervention 2**: Fones (21/21)<br>**2st preschool**<br>**Intervention 1**: horizontal scrubbing (22/22)<br>**Intervention 2**: Fones (25/25)<br>**3st preschool**<br>**Intervention 1**: horizontal scrubbing (39/39)<br>**Intervention 2**: Fones (39/39)<br>**Professional tooth cleaning:** no<br>**Referral of the instruction to original publication of the technique**: no reference for either technique<br>**Instruction describes the technique applied like the original**: n.i.
Provided devices	Toothbrush and toothpaste appropriate for this age group
Provided instructions	**All groups:**<br>Brushing twice/day<br>**Intervention groups:**<br>Additional instruction with respect to respective brushing technique<br>**Mode**: via hands-on; interactive oral and dental health education session on models accompanied by powerpoint-presentation<br>1st preschool: Educational toothbrushing card for the children<br>2st preschool: oral and dental health education lecture for parents<br>3st preschool: awarding card at subsequent follow up<br>**Frequency**: 1x at baseline (T1)<br>**Provider/qualification**: n.i.<br>**Instruction for application at home**: no
Outcome parameters<br><br>Outcome dates:	**Primary (plaque after toothbrushing):** PI<br>**Secondary (Gingivitis):** not available<br><br>**Primary (last date of outcome assessment):** not available (n.i. whether plaque was assessed after toothbrushing at T2-T5)<br>**Secondary (first date of outcome assessment):** T1
**Deinzer et al. (2016)**	Included in NMA
Study design	**Arms**: 3 parallel<br>**Blinding**: examiners blinded, participants blinded to hypotheses<br>**Standardized instruction of the technique**: yes<br>**Calibration of examiners**: yes
Participants	**Sample size**: N=93 (n=92 received allocated intervention)<br>**Gender**: 58 female/ 34 male<br>**Age**: 30-82 years, mean 59.3<br>**Educational level**: good language knowledge<br>**Dental education**: no<br>**General health status**: no physical, mental disabilities that influence oral hygiene<br>**Smoking status**: n.r.<br>**Dental status and/or periodontal status**: no dental or periodontal treatment needed; ≥ 4 sufficient crowns; ≥ 4 natural teeth
Interventions<br>(n included/completed)	**Control:** no specific technique (30/25)<br>**Intervention 1**: Fones (32/27)<br>**Intervention 2**: modified Bass (31/27)

	**Professional tooth cleaning:** yes – prior to intervention<br>**Referral of the instruction to original publication of the technique**: Bass: refers to Bass (1954) but is described as in Katz (1972); Fones: refers to Fones (1921)<br>**Instruction describes the technique applied like the original**: Bass: in analogy to Katz (1972); Fones: in analogy to Fones (1921; description differs with respect to inner surfaces)
Provided devices	toothbrush (elmex® interX); toothpaste; floss (elmex®), superfloss (meridol) interdental brushes (elmex®)
Provided instructions	**<u>All groups:</u>**<br>computer-based training in basics of toothbrushing<br>**<u>Intervention groups:</u>**<br>additional slides with instruction with respect to the respective brushing technique<br>**Mode**: computer-based training (powerpoint slides); patients worked through the presentations alone in their own pace<br>**Frequency**: 1x between baseline (T1) and six weeks (T2)<br>**Provider/qualification**: brushing was assessed by a dentist immediately after instruction; reinstructions were given when necessary<br>**Instruction for application at home**: leaflet with the basics of tooth brushing <u>for all groups</u> and respective additional information for intervention groups
Outcome parameters<br><br>Outcome dates:	**Primary (plaque after toothbrushing):** MPI (included in NMA); TQHI<br>**Secondary (Gingivitis):** not available<br><br>**Primary (last date of outcome assessment):** 12 weeks after intervention (T3)<br>**Secondary (first date of outcome assessment):** 6 weeks after intervention (T2)<br>Other: not available
**Dosumu et al., 2019**	Not included in NMA
Study design	**Arms**: 2 parallel<br>**Blinding**: examiners blinded<br>**Standardized instruction of the technique**: n.i.<br>**Calibration of examiners**: yes
Participants	**Sample size**: N=50<br>**Gender:** male:female ratio 2:3<br>**Age**: mean 23.1<br>**Educational level**: students (university admission)<br>**Dental education**: (preclinical) dental students<br>**General health status:** n.r.<br>**Smoking status**: n.r.<br>**Dental status and/or periodontal status**: ≥ 6 natural teeth/quadrant, no dentures or other dental prostheses; no teeth crowding teeth in good alignment; no periodontal disease
Interventions<br>(n included/completed)	**Control:** no specific technique (25/25)<br>**Intervention**: modified Bass technique (25/25)<br>**Professional tooth cleaning:** yes – prior intervention<br>**Referral of the instruction to original publication of the technique**: no<br>**Instruction describes the technique applied like the original**: n.i.
Provided devices	toothbrush (medium); fluoridated toothpaste
Provided instructions	**<u>All groups:</u>**<br>Brushing twice/day (before breakfast; after dinner) 5 minutes<br>**<u>Intervention group:</u>**<br>additional instruction with respect to brushing technique<br>**Mode:** video and dental model; hands on<br>**Frequency**: 1x at baseline (T1)<br>**Provider/qualification**: n.i.

	**Instruction for application at home**: no
Outcome parameters	**Primary (plaque after toothbrushing):** n.i. whether plaque was assessed after toothbrushing<br>**Secondary (Gingivitis):** BOP
Outcome dates	**Primary (last date of outcome assessment):** 28 days (T3)<br>**Secondary (first date of outcome assessment):** 7 days (T2)<br>Other: not available
**Harnacke et al. (2012)**	Included in NMA
Study design	**Arms**: 3 parallel<br>**Blinding**: examiners blinded, participants blinded to hypotheses<br>**Standardized instruction of the technique**: yes<br>**Calibration of examiners**: yes
Participants	**Sample size**: N=67 (n=64 received allocated intervention)<br>**Gender**: 44 female/ 12 male<br>**Age**: mean 23.2<br>**Educational level**: students (university admission)<br>**Dental education**: no<br>**General health status**: no physical, mental disabilities that influence oral hygiene; no serious disease*<br>**Smoking status**: non-smokers<br>**Dental status and/or periodontal status**: ≥ 20 natural teeth; ≥ 10 teeth showing plaque or bleeding
Interventions<br>(n included/completed)	**Control:** no specific technique (23/19)<br>**Intervention 1**: Fones (22/19)<br>**Intervention 2**: modified Bass (22/18)<br>**Professional tooth cleaning:** yes – prior to intervention<br>**Referral of the instruction to original publication of the technique**: Bass: refers to Hellwege (2003) and APA; Fones: refers to APA<br>**Instruction describes the technique applied like the original**: Bass: in analogy to Katz (1972); Fones: in analogy to Fones (1921; description differs with respect to inner surfaces)
Provided devices	toothbrush (elmex® interX); toothpaste (elmex®); floss (elmex® waxed and unwaxed)
Provided instructions	**All groups:**<br>computer-based training in basics of toothbrushing; video instruction “how to floss”<br>**Intervention groups:**<br>additional slides with instruction with respect to the respective brushing technique<br>**Mode**: computer-based training (powerpoint slides and video instruction); patients worked through the presentations alone in their own pace<br>**Frequency**: 1x between baseline (T1) and six weeks (T2)<br>**Provider/qualification**: brushing was assessed by a dentist immediately after instruction; reinstructions were given when necessary<br>**Instruction for application at home**: leaflet with the basics of tooth brushing for all groups and respective additional information for intervention groups
Outcome parameters	Primary (Plaque): MPI (included in NMA); TQHI<br>**Secondary (Gingivitis):** PBI
Outcome dates	**Primary (last date of outcome assessment):** 28 weeks after intervention (T4)<br>**Secondary (first date of outcome assessment):** 6 weeks after intervention (T2)<br>Other: 12 weeks after intervention (T3)
**Harnacke et al. (2016)**	Included in NMA
Study design	**Arms**: 3 parallel<br>**Blinding**: examiners blinded, participants blinded to hypotheses<br>**Standardized instruction of the technique**: yes

	**Calibration of examiners**: yes
Participants	**Sample size**: N=70 (n=69 received allocated intervention)<br>**Gender**: 45 female/ 25 male<br>**Age**: 18-19 years old<br>**Educational level**: n.r.<br>**Dental education**: no<br>**General health status**: no physical, mental disabilities that influence oral hygiene; no serious disease*<br>**Smoking status**: n.r.<br>**Dental status and/or periodontal status**: n.r.
Interventions<br>(n included/completed)	**Control:** no specific technique (23/23)<br>**Intervention 1**: Fones (23/23)<br>**Intervention 2**: modified Bass (24/23)<br>**Professional tooth cleaning:** yes – prior to intervention<br>**Referral of the instruction to original publication of the technique**: no<br>**Instruction describes the technique applied like the original**: no description but refers to Harnacke et al. (2012)
Provided devices	toothbrush (elmex® interX); toothpaste (elmex®); floss (elmex® waxed and unwaxed)
Provided instructions	**<u>All groups:</u>**<br>computer-based training in basics of toothbrushing<br>**<u>Intervention groups:</u>**<br>additional slides with instruction with respect to the respective brushing technique<br>**Mode**: computer-based training (powerpoint slides); patients worked through the presentations alone in their own pace<br>**Frequency**: 1x between baseline (T1) and six weeks (T2)<br>**Provider/qualification**: brushing was assessed by a dentist immediately after instruction; reinstructions were given when necessary<br>**Instruction for application at home**: leaflet with the basics of tooth brushing <u>for all groups</u> and respective additional information for intervention groups
Outcome parameters<br><br><br>Outcome dates	Primary (Plaque): MPI<br>**Secondary (Gingivitis):** BOP<br><br>**Primary (last date of outcome assessment):** 28 weeks after intervention (T4)<br>**Secondary (first date of outcome assessment):** 6 weeks after intervention (T2)<br>Other: 12 weeks after intervention (T3)
**Janakiram et al., 2020**	Not included in NMA
Study design	**Arms**: 2 parallel<br>**Blinding**: examiners blinded<br>**Standardized instruction of the technique**: n.i.<br>**Calibration of examiners**: yes
Participants	**Sample size**: N=120<br>**Gender**: 66.6% female<br>**Age**: 18-30 years; mean 22.0<br>**Educational level**: students (university admission)<br>**Dental education**: dental students<br>**General health status**: n:r.<br>**Smoking status**: non-smokers<br>**Dental status and/or periodontal status**: ≥ 20 teeth; mild to moderate gingivitis; no history of periodontal therapy or disease
Interventions<br>(n included/completed)	**Control:** no specific technique (40/40)<br>**Intervention 1**: Fones (40/40)<br>**Intervention 2**: modified Bass (40/38)<br>**Professional tooth cleaning:** no

	**Referral of the instruction to original publication of the technique**: no<br>**Instruction describes the technique applied like the original**: modified Bass: in analogy to Katz (1972); Fones: in analogy to Fones (1921)<br>*Note: instruction not described in the paper but in the clinical trials registry form*
Provided devices	toothbrush (standard, soft bristled); toothpaste (Colgate Total® Advanced Health)
Provided instructions	**<u>All groups:</u>**<br>Instruction: brushing twice a day using toothbrush and toothpaste<br><u>Intervention groups</u><br>**Mode:** computer-based training (powerpoint slides and videos); demonstration on study models;<br>**Frequency**: 4x at baseline (T1), 24h (T2), one week (T3) and four weeks (T4)<br>**Provider/qualification**: brushing technique was performed in the presence of the trial coordinator during each visit<br>**Instruction for application at home**: leaflet describing respective brushing technique
Outcome parameters<br><br>Outcome dates	**Primary (plaque after toothbrushing):** n.i. whether plaque was assessed after toothbrushing<br>**Secondary (Gingivitis):** GI<br><br>**Primary (last date of outcome assessment):** not applicable (instruction at each time point)<br>**Secondary (first date of outcome assessment):** 4 weeks after intervention (T4) (additional instructions and assessments 24h and 1 week after intervention)
**Kanchanakamol et al., 1993**	Not included in NMA
Study design	**Arms**: 4 parallel<br>**Blinding**: n.i.<br>**Standardized instruction of the technique**: n.i.<br>**Calibration of examiners**: yes
Participants	**Sample size**: N=100 (n=92 received allocated intervention)<br>**Gender:** n.i.<br>**Age**: 21-22 years<br>**Educational level**: soldiers of Air Base<br>**Dental education**: no<br>**General health status:** n.r.<br>**Smoking status**: n.r.<br>**Dental status and/or periodontal status**: no dental or mucosal disease; teeth in good alignment
Interventions<br>(n included/completed)	**Intervention 1**: Roll technique (standard toothbrush) (25/23)<br>**Intervention 2**: Bass technique (standard toothbrush) (25/23)<br>**Intervention 3**: Roll technique (Concept 45° toothbrush) (25/23)<br>**Intervention 4:** Bass technique (Concept 45° toothbrush) (25/23)<br>**Professional tooth cleaning:** dental scaling (by dental students) prior intervention<br>**Referral of the instruction to original publication of the technique**: Roll technique: refers to Hansen & Giermo (1971; the cited paper does not describe a roll technique); Bass: refers to Bass (1954)<br>**Instruction describes the technique applied like the original**: Roll: see above; Bass: in analogy to Bass (1954)
Provided devices	Standard toothbrush; Concept 45° toothbrush
Provided instructions	**<u>All groups:</u>**<br>Brushing twice/day 2 minutes<br>**<u>Intervention groups:</u>**<br>Oral hygiene instruction with respect to the respective brushing technique<br>**Mode:** instruction in small groups for 2h<br>**Frequency**: 1x at baseline (T1)<br>**Provider/qualification**: n.i.<br>**Instruction for application at home**: no

Outcome parameters<br><br>Outcome dates:	**Primary (plaque after toothbrushing):** modified Navy Plaque Index<br>**Secondary (Gingivitis):** not available<br><br>**Primary (last date of outcome assessment):** 2 weeks after intervention (T2)<br>**Secondary (first date of outcome assessment):** not applicable (identical to T2)<br>Other: not available
**Sarvia et al., 1989**	Not included in NMA
Study design	**Arms**: 4 parallel<br>**Blinding**: n.i.<br>**Standardized instruction of the technique**: n.i.<br>**Calibration of examiners**: n.i.
Participants	**Sample size**: N=64<br>**Gender:** 36 female/ 28 male<br>**Age**: 4-12 years<br>**Educational level**: (pre)school children<br>**Dental education**: no<br>**General health status:** no mental retardation / no physical condition compromising perceptual or motor functioning<br>S**moking status**: n.r.<br>**Dental status and/or periodontal status**: no caries
Interventions<br>(n included/completed)	**Control 1:** no specific technique (17/17)<br>**Control 2:** no specific technique plus incentive (14/14)<br>**Intervention 1:** horizontal scrubbing (15/15)<br>**Intervention 2:** horizontal scrubbing plus incentive (18/18)<br>**Professional tooth cleaning:** no<br>**Referral of the instruction to original publication of the technique**: no<br>**Instruction describes the technique applied like the original**: not applicable
Provided devices	Toothbrush
Provided instructions	Control groups:<br>no instruction<br>**Intervention groups:**<br>instruction in horizontal scrubbing<br>Mode: Individual instruction<br>**Frequency**: 1x at baseline (T1)<br>**Provider/qualification**: n.i.<br>**Instruction for application at home**: no
Outcome parameters<br><br>Outcome dates	**Primary (plaque after toothbrushing):** Plaque (n.i. about the index)<br>**Secondary (Gingivitis):** not available<br><br>**Primary (last date of outcome assessment):** 1 week after intervention (T2)<br>**Secondary (first date of outcome assessment):** not applicable (identical to T2)<br>Other: not available
**Schlueter et al., 2013**	Included in NMA
Study design	**Arms**: 3 parallel<br>**Blinding**: examiner 1 (plaque assessment) not blinded; examiner 2 (video recording) blinded<br>**Standardized instruction of the technique**: yes<br>**Calibration of examiners**: yes
Participants	**Sample size**: N=98<br>**Gender**: not reported<br>**Age**: mean 26.6<br>**Educational level**: students (university admission)<br>**Dental education**: no

	**General health status**: no physical, mental disabilities that influence oral hygiene; no serious disease*<br>**Smoking status**: n.r.<br>**Dental status and/or periodontal status**: ≥ 24 teeth; no serious periodontitis no multiple recessions (1/3 of root lengths)
Interventions<br>(n included/completed)	**Control:** no specific technique (33/27)<br>**Intervention 1**: modified Bass (leaflet) (32/24)<br>**Intervention 2**: modified Bass (demonstration) (33/26)<br>**Professional tooth cleaning:** yes – prior intervention<br>**Referral of the instruction to original publication of the technique**: no<br>**Instruction describes the technique applied like the original**: modified Bass: in analogy to Katz (1972; the authors instructed 3-5 jiggling and wiping cycles per area*)
Provided devices	toothbrush (elmex® interX)
Provided instructions	**Control group:**<br>Instruction 4 weeks after last visit: verbal instruction<br>**Intervention groups:**<br>**Mode:**<br>Intervention 1: verbal instruction and a leaflet containing major steps the technique and brushing sequence<br>Intervention 2: verbal instruction supported by demonstration with a model<br>**Frequency**: 2x at baseline (T1) and 2 weeks (T2)<br>**Provider/qualification**: a dentist trained and reevaluated participants' technique during the study<br>**Instruction for application at home**: no
Outcome parameters<br><br><br>Outcome dates	Primary (Plaque): TQHI<br>**Secondary (Gingivitis):** not available<br><br>**Primary (last date of outcome assessment):** 4 weeks after intervention (T3)<br>**Secondary (first date of outcome assessment):** 2 weeks after intervention (T2)<br>Other: not available
**Schmalz et al., 2018**	Included in NMA
Study design	**Arms**: 6 parallel (2 arms relevant for review)<br>**Blinding**: examiner blinded<br>**Standardized instruction of the technique**: n.i.<br>**Calibration of examiners**: yes
Participants	**Sample size**: N=50 (n=48 received allocated intervention)<br>**Gender:** 39 female/ 9 male<br>**Age**: 18-30 years; mean 23.7<br>**Educational level**: students (university admission)<br>**Dental education**: no<br>**General health status:** no physical, mental disabilities that influence oral hygiene; no serious disease<br>**Smoking status**: n.r.<br>**Dental status and/or periodontal status**: ≥ 20 teeth; healthy oral condition (PSI ≤ 2)
Interventions<br>(n included/completed)	**Control:** no specific technique (24/22)<br>**Intervention**: Fones (24/22)<br>**Professional tooth cleaning:** yes – prior intervention<br>**Referral of the instruction to original publication of the technique**: no<br>**Instruction describes the technique applied like the original**: in analogy to Fones (1921; description differs with respect to inner surfaces)
Provided devices	toothbrush (elmex® interX); toothpaste (Sensodyne)
Provided instructions	**All groups:**

	Brushing twice/day 2-3 minutes<br>**Intervention group:**<br>additional instruction with respect to brushing technique<br>**Mode:** oral instruction and demonstration on model and hands on*<br>**Frequency**: 1x at baseline (T1)<br>**Provider/qualification**: dentist<br>**Instruction for application at home**: no
Outcome parameters<br><br><br>Outcome dates	**Primary (plaque after toothbrushing):** not available<br>**Secondary (Gingivitis):** GI (included in NMA); PBI<br><br>**Primary (last date of outcome assessment):** 12 weeks after intervention (T3)<br>**Secondary (first date of outcome assessment):** 2 weeks after intervention (T1)<br>Other: 4 weeks after intervention (T2)
**Smutkeeree et al., 2011**	Included in NMA
Study design	**Arms**: 2 parallel<br>**Blinding**: n.i.<br>**Standardized instruction of the technique**: n.i.<br>**Calibration of examiners**: n.i.
Participants	**Sample size**: N=60 (n=57 received allocated intervention)<br>**Gender**: 26 female/ 34 male<br>**Age**: 10 – 12 years; mean 11.0<br>**Educational level**: school children<br>**Dental education**: no<br>**General health status**: visually impaired otherwise healthy children<br>**Smoking status**: n.r.<br>**Dental status and/or periodontal status**: ≥ 4 teeth/quadrant; periodontal healthy condition
Interventions<br>(n included/completed)	**Intervention 1**: modified Bass (28/28)<br>**Intervention 2**: horizontal scrubbing (29/29)<br>**Professional tooth cleaning:** no<br>**Referral of the instruction to original publication of the technique**: no<br>**Instruction describes the technique applied like the original**: n.i.
Provided devices	Toothbrush (soft, round-end bristles, straight angled (Berman Plus Child))
Provided instructions	**All groups:**<br>Brushing twice/day (morning and before bedtime) 2 minutes<br>**Intervention groups:**<br>additional instruction with respect to the respective brushing technique<br>**Mode**: individually verbal and tactile<br>**Frequency**: 1x at baseline (T1)<br>**Provider/qualification**: n.i.<br>**Instruction for application at home**: no
Outcome parameters<br><br><br>Outcome dates	**Primary (plaque after toothbrushing):** n.i. whether plaque was assessed after toothbrushing<br>**Secondary (Gingivitis):** GI<br><br>**Primary (last date of outcome assessment):** 6 months after intervention (T3)<br>**Secondary (first date of outcome assessment):** 1 month after intervention (T2)<br>Other: not available
**Zhang et al., 2005**	Not included in NMA
Study design	**Arms**: 2 crossover (each technique in counterbalanced order)<br>**Blinding**: examiner blinded<br>**Standardized instruction of the technique**: n.i.<br>**Calibration of examiners**: n.i.

Participants	**Sample size**: N=40<br>**Gender:** 27 female/ 13 male<br>**Age**: 18-35 years<br>**Educational level**: students (university admission)<br>**Dental education**: no<br>**General health status:** no systemic diseases<br>**Smoking status**: n.r.<br>**Dental status and/or periodontal status**: ≥ 24 teeth in good alignment; no or only marginal gingivitis and no periodontitis, no denture or cervical fillings
Interventions<br>(n included/completed)	**Intervention 1:** modified Bass (40)<br>**Intervention 2**: vertical brushing (40)<br>**Professional tooth cleaning:** yes – before the instruction of the respective technique (day 0 and day 14)<br>**Referral of the instruction to original publication of the technique**: Bass: refers to Bass (1948); vertical brushing: no<br>**Instruction describes the technique applied like the original**: n.i.
Provided devices	Toothbrush (crest flat head soft bristle); toothpaste (fluoridated)
Provided instructions	<u>**Intervention groups:**</u><br>Instruction with respect to the respective brushing technique<br>**Mode:** individual instruction on the model and practice with further help<br>**Frequency**: days 1-4: 2x/day instruction and correction of brushing by assistant with 1st technique; days 15-19: 2x/day instruction and correction of brushing by assistant with 2nd technique<br>**Provider/qualification**: study assistant<br>**Instruction for application at home**: no
Outcome parameters<br><br>Outcome dates	**Primary (plaque after toothbrushing):** Modified Benson Proximal Marginal Index<br>**Secondary (Gingivitis):** not available<br><br>**Primary (last date of outcome assessment):** day 6 (T1) and day 20 (T2)<br>**Secondary (first date of outcome assessment):** not available<br>Other: not available
