# Supplementary material for: Manual toothbrushing techniques for plaque removal and the prevention of gingivitis—A systematic review with network meta-analysis
Source: PLoS One. 2024 Jul 5;19(7):e0306302. doi: 10.1371/journal.pone.0306302 (PMC11226064; doi:10.1371/journal.pone.0306302)
Supplement: S2 Appendix — Lists the reports excluded after full text reading and provides the reasons for exclusion. Also lists the reports that were not accessible. (PDF) [file pone.0306302.s002.pdf]

Appendix S2: Reports that could not be obtained and reports excluded after full-text reading

Study	Reason for Exlusion	Identifier (DOI, PMID)
Allen et al. (1992)	reports not accessible: only conference abstract available	https://www.cochranelibrary.com/central/doi/10.1002/central/CN-00670514/full
Allet et al. (1972)	intervention: no training of a technique	4502488
Arai & Kinoshita (1977)	study type: no randomization	10.11480/btmd.240205
Battaglia (2008)	intervention: no comparison of techniques	10.1111/j.1601-5037.2008.00302.x
Curto-Manrique et al. (2019)	intervention: not self brushed	10.4103/JISPPD.JISPPD_274_18
da Silva et al. (1978)	study type: no randomization	283460
Disyam (1987)	study type: no randomization	3483850
Frandsen et al. (1970)	intervention: no comparison of techniques	10.1111/j.1600-0722.1970.tb02098.x
Frandsen et al. (1972)	outcome: no information available whether plaque was assessed after toothbrushing	10.1111/j.1600-0722.1972.tb00290.x
Gibson & Wade (1977)	intervention: randomization confounded with brush type	10.1902/jop.1977.48.8.456
Gonçalves et al. (2007)	intervention: other research question; only occlusal surfaces of the eruption teeth brushed	17366759
Guedes-Pinto et al. (1978)	study type: no randomization	293810
Hapsari et al. (2020)	study type: no randomization	10.4103/jioh.jioh_175_19
Hotz et al. (1984)	population: laboratory model, no humans	6591418
Ju et al. (2019)	outcome: no information available on request to authors whether plaque was assessed after toothbrushing	10.1016/j.apnr.2018.12.008
Kardel & Bay (1973)	study type: no randomization	4536268
Kitahara et al. (1990)	study type: no randomization	10.2329/perio.32.299
Kremers et al. (1978)	study type: no randomization	271589
Lee et al. (2020)	intervention: no self-applied toothbrushing	10.5051/jpis.2020.50.2.83
Morita et al. (1998)	intervention: no self-applied toothbrushing; no randomization	10.1111/j.1600-051x.1998.tb02377.x
Mun et al. (2014)	intervention: no comparison of techniques; unclear whether self-applied toothbrushing	10.1111/idh.12053
Ott et al. (1991)	population: laboratory model, no humans	1818627
Poyato-Ferrera et al. (2003)	study type: no randomization	10.1034/j.1601-5037.2003.00018.x
Robinson (1976)	outcome: no information available whether plaque was assessed after toothbrushing	10.2105/ajph.66.11.1078
Sangnes (1974)	study type: no randomization (4 conditions were randomly allocated to 4 clusters, i.e. school classes, but not to participants)	4594788
Sangnes et al. (1972)	study type: no randomization	4552613
Schlueter et al. (2010)	outcome: neither plaque nor bleeding assessed	10.1007/s00784-009-0269-1
Schlueter et al. (2021)	study type: no randomization	10.1371/journal.pone.0261496
Setiyohadi (1997)	reports not accessible: only conference abstract available	https://www.cochranelibrary.com/central/doi/10.1002/central/CN-00432612/full
Shick et al. (1961)	study type: no randomization	10.1902/jop.1961.32.4.346
Shin (1987)	intervention: no comparison of techniques	3474326
Shoji et al. (1988)	intervention: no comparison of techniques	10.2329/perio.30.566
Sousa (1999)	reports not accessible: only conference abstract available	https://www.webofscience.com/wos/woscc/full-record/WOS:000080316600751
Suppipat (1973)	reports not accessible: not available	4527263
Ueda & Ono (1979)	reports not accessible: not available	292118
Ueda et al. (1981)	intervention: no comparison of techniques	10.2329/perio.23.367
Watanabe et al. (1987)	intervention: no comparison of techniques	10.2329/perio.29.610
Weng et al. (2023)<br>*result from supplemental search*	outcome: no information available on request to authors whether plaque was assessed after toothbrushing	10.1016/j.jdent.2023.104571
Yankell et al. (1993)	intervention: no comparison of techniques	8267875
