# Supplementary material for: Manual toothbrushing techniques for plaque removal and the prevention of gingivitis—A systematic review with network meta-analysis
Source: PLoS One. 2024 Jul 5;19(7):e0306302. doi: 10.1371/journal.pone.0306302 (PMC11226064; doi:10.1371/journal.pone.0306302)
Supplement: S4 Appendix — Contains all the outputs of CINeMA and the final evaluation of confidence in the evidence. (PDF) [file pone.0306302.s004.pdf]

Appendix S4. CINeMA outputs.

This supplementary information contains all outputs of the CINeMA tool. The CINeMA outputs show the results for the different outcomes (primary plaque, secondary plaque, primary gingivitis, secondary gingivitis) for all studies and for studies with adults only.

Table of contents

1. CINeMA-Output: **Primary Plaque (all studies)** 2
2. CINeMA-Output: **Secondary Plaque (all studies)** 4
3. CINeMA-Output: **Secondary Plaque (studies with adults only)** 8
4. CINeMA-Output: **Primary Gingivitis (all studies)** 10
5. CINeMA-Output: **Primary Gingivitis (studies with adults only)** 13
6. CINeMA-Output: **Secondary Gingivitis (all studies)** 16
7. CINeMA-Output: **Secondary Gingivitis (studies with adults only)** 19

1. CINeMA-Output: Primary Plaque (all studies)

	id	study	year	feature	outcome	index	t1	n1	y1	sd1	rob	indirectness	t2	n2	y2	sd2
1	2	Harnacke et al. (2012)	2012	plaque	primary	MPI	Control	19	58.71	18.63	2	1	Fones	19	52.57	17.01
2	2	Harnacke et al. (2012)	2012	plaque	primary	MPI	Control	19	58.71	18.63	2	1	Bass	18	63.59	17.03
3	2	Harnacke et al. (2012)	2012	plaque	primary	MPI	Fones	19	52.57	17.01	2	1	Bass	18	63.59	17.03
4	3	Schlueter et al. (2013.1)	2013	plaque	primary	TQHI	Control	27	1.72	0.48	3	1	Bass	24	1.52	0.58
5	4	Schlueter et al. (2013.2)	2013	plaque	primary	TQHI	Control	27	1.72	0.48	3	1	Bass	26	1.5	0.69
6	5	Deinzer et al. (2016.1)	2016	plaque	primary	MPI	Control	30	70.04	16	1	1	Fones	32	71.89	15.29
7	5	Deinzer et al. (2016.1)	2016	plaque	primary	MPI	Control	30	70.04	16	1	1	Bass	30	67.18	18.21
8	5	Deinzer et al. (2016.1)	2016	plaque	primary	MPI	Fones	32	71.89	15.29	1	1	Bass	30	67.18	18.21
9	6	Deinzer et al. (2016.2)	2016	plaque	primary	MPI	Control	30	34.34	20.13	1	1	Fones	32	31.9	20.35
10	6	Deinzer et al. (2016.2)	2016	plaque	primary	MPI	Control	30	34.34	20.13	1	1	Bass	30	31.23	22.73
11	6	Deinzer et al. (2016.2)	2016	plaque	primary	MPI	Fones	32	31.9	20.35	1	1	Bass	30	31.23	22.73
12	7	Harnacke et al. (2016)	2016	plaque	primary	MPI	Control	22	78.89	10.66	1	1	Fones	23	72.87	15.69
13	7	Harnacke et al. (2016)	2016	plaque	primary	MPI	Control	22	78.89	10.66	1	1	Bass	23	80.93	13.39
14	7	Harnacke et al. (2016)	2016	plaque	primary	MPI	Fones	23	72.87	15.69	1	1	Bass	23	80.93	13.39

Node size by: Sample size **Node color by:** Risk of Bias **Edge width by:** Sample Size **Edge color by:** Average RoB

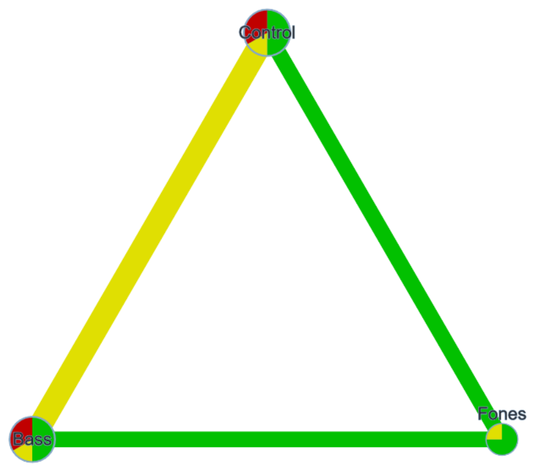

Risk of bias contributions: The bar chart shows the contributions of each piece of study to the network estimate

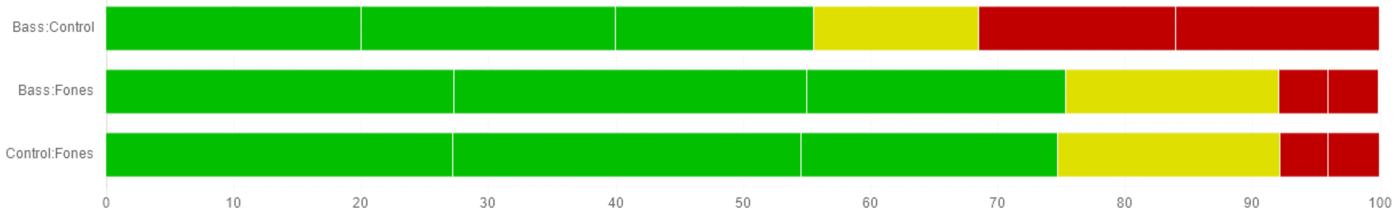

Selected Rule: *Average RoB*

Comparison	Bass:Control
Evidence: mixed	
Majority RoB:	No concerns
Average RoB:	Some concerns
Highest RoB:	Major concerns
NMA judgment	Some concerns

Comparison	Bass:Fones
Evidence: mixed	
Majority RoB:	No concerns
Average RoB:	No concerns
Highest RoB:	Major concerns
NMA judgment	No concerns

Comparison	Control:Fones
Evidence: mixed	
Majority RoB:	No concerns
Average RoB:	No concerns
Highest RoB:	Major concerns
NMA judgment	No concerns

Reporting Bias: Judgement „all low Risk“

Indirectness: Judgement “all no concerns”

Imprecision: Clinically important effect size set at 0.2

Comparison **Bass:Control**
Evidence: mixed
NMA estimate: **-0.129**
95% Confidence interval: **(-0.377,0.119)**
Confidence interval extends into clinically important effects
Imprecision judgment: Some concerns

Comparison **Bass:Fones**
Evidence: mixed
NMA estimate: **0.091**
95% Confidence interval: **(-0.200,0.382)**
*Confidence interval extends into clinically important effects in **both** directions*
Imprecision judgment: Major concerns

Comparison **Control:Fones**
Evidence: mixed
NMA estimate: **0.220**
95% Confidence interval: **(-0.070,0.510)**
Confidence interval extends into clinically important effects
Imprecision judgment: Some concerns

Heterogeneity

The estimated value of between-study variance for the network meta-analysis is **0.017**

Comparison **Bass:Control**
Evidence: mixed
NMA estimate: **-0.129**
95% intervals for NMA estimate
Confidence interval: **(-0.377,0.119)**
Prediction interval: **(-0.557,0.299)**
*Prediction interval **extends** into clinically important or unimportant effects*
Heterogeneity judgment: Some concerns

Comparison **Bass:Fones**
Evidence: mixed
NMA estimate: **0.091**
95% intervals for NMA estimate
Confidence interval: **(-0.200,0.382)**
Prediction interval: **(-0.375,0.557)**
*Confidence and prediction intervals **agree** in relation to clinically important effect*
Heterogeneity judgment: No concerns

Comparison **Control:Fones**
Evidence: mixed
NMA estimate: **0.220**
95% intervals for NMA estimate
Confidence interval: **(-0.070,0.510)**
Prediction interval: **(-0.245,0.685)**
*Prediction interval **extends** into clinically important or unimportant effects*
Heterogeneity judgment: Some concerns

Incoherence

Global test based on a random-effects design-by-treatment interaction model
χ^2 statistic: 2.010 (1 degrees of freedom), P value: 0.156

Comparison **Bass:Control**
Evidence: direct
Direct standardised mean difference: **-0.128(-0.376,0.120)**
Inconsistency measures: Not applicable
Incoherence judgment: No concerns

Comparison **Bass:Fones**
Evidence: mixed
NMA standardised mean difference: **0.091(-0.200,0.382)**
Direct standardised mean difference: **0.153(-0.151,0.458)**
Indirect standardised mean difference: **-0.563(-1.552,0.426)**
Inconsistency measures
Difference of standardised mean differences: **0.717(-0.318,1.751)**
P value: **0.175**
Incoherence judgment: No concerns

Comparison **Control:Fones**
Evidence: mixed
NMA standardised mean difference: **0.220(-0.070,0.510)**
Direct standardised mean difference: **0.164(-0.139,0.467)**
Indirect standardised mean difference: **0.831(-0.171,1.832)**
Inconsistency measures
Difference of standardised mean differences: **-0.666(-1.713,0.380)**
P value: **0.212**
Incoherence judgment: No concerns

Overall rating of the confidence into the evidence

<table>
<tr><th>Comparison</th><th>Number of Studies</th><th>Within-study bias</th><th>Reporting bias</th><th>Indirectness</th><th>Imprecision</th><th>Heterogeneity</th><th>Incoherence</th><th>Confidence rating</th><th>Reason(s) for downgrading</th></tr>
<tr><td colspan="10">Mixed evidence</td></tr>
<tr><td>Bass vs Control</td><td>6</td><td>Some concerns</td><td>Low risk</td><td>No concerns</td><td>Some concerns</td><td>Some concerns</td><td>No concerns</td><td>Low</td><td>| Within-study bias | Imprecision | Heterogeneity |</td></tr>
<tr><td>Bass vs Fones</td><td>4</td><td>No concerns</td><td>Low risk</td><td>No concerns</td><td>Major concerns</td><td>No concerns</td><td>No concerns</td><td>Low</td><td>| Imprecision |</td></tr>
<tr><td>Control vs Fones</td><td>4</td><td>No concerns</td><td>Low risk</td><td>No concerns</td><td>Some concerns</td><td>Some concerns</td><td>No concerns</td><td>Moderate</td><td>| Imprecision | Heterogeneity |</td></tr>
</table>

2. CINeMA-Output: Secondary Plaque (all studies)

	id	study	year	feature	outcome	index	t1	n1	y1	sd1	rob	indirectness	t2	n2	y2	sd2
1	2	Harnacke et al. (2012)	2012	plaque	secondary	MPI	Control	19	62.51	20.05	2	1	Fones	18	50.19	15.33
2	2	Harnacke et al. (2012)	2012	plaque	secondary	MPI	Control	19	62.51	20.05	2	1	Bass	18	64.72	19.31
3	2	Harnacke et al. (2012)	2012	plaque	secondary	MPI	Fones	18	50.19	15.33	2	1	Bass	18	64.72	19.31
4	3	Schlueter et al. (2013.1)	2013	plaque	secondary	TQHI	Control	27	1.8	0.47	3	1	Bass	24	1.58	0.58
5	4	Schlueter et al. (2013.2)	2013	plaque	secondary	TQHI	Control	27	1.8	0.47	3	1	Bass	26	1.64	0.58
6	5	Deinzer et al. (2016.1)	2016	plaque	secondary	MPI	Control	30	76.18	14.93	1	1	Fones	32	71.99	14.92
7	5	Deinzer et al. (2016.1)	2016	plaque	secondary	MPI	Control	30	76.18	14.93	1	1	Bass	30	70.56	16.9
8	5	Deinzer et al. (2016.1)	2016	plaque	secondary	MPI	Fones	32	71.99	14.92	1	1	Bass	30	70.56	16.9
9	6	Deinzer et al. (2016.2)	2016	plaque	secondary	MPI	Control	30	42.18	25.56	1	1	Fones	32	36.78	23.99
10	6	Deinzer et al. (2016.2)	2016	plaque	secondary	MPI	Control	30	42.18	25.56	1	1	Bass	30	39.24	23.41
11	6	Deinzer et al. (2016.2)	2016	plaque	secondary	MPI	Fones	32	36.78	23.99	1	1	Bass	30	39.24	23.41
12	7	Harnacke et al. (2016)	2016	plaque	secondary	MPI	Control	22	77.45	12.08	1	1	Fones	23	70.67	14.49
13	7	Harnacke et al. (2016)	2016	plaque	secondary	MPI	Control	22	77.45	12.08	1	1	Bass	23	76.05	17.26
14	7	Harnacke et al. (2016)	2016	plaque	secondary	MPI	Fones	23	70.67	14.49	1	1	Bass	23	76.05	17.26
15	8	Ceyhan et al. (2018.1)	2018	plaque	secondary	PI	Scrub	17	0.3	0.04	2	3	Fones	21	0.25	0.04
16	9	Ceyhan et al. (2018.2)	2018	plaque	secondary	PI	Scrub	22	0.37	0.03	2	3	Fones	25	0.47	0.04
17	10	Ceyhan et al. (2018.3)	2018	plaque	secondary	PI	Scrub	39	0.23	0.02	2	3	Fones	39	0.18	0.02

Node size by: Sample size **Node color by:** Risk of Bias **Edge width by:** Sample Size **Edge color by:** Average RoB

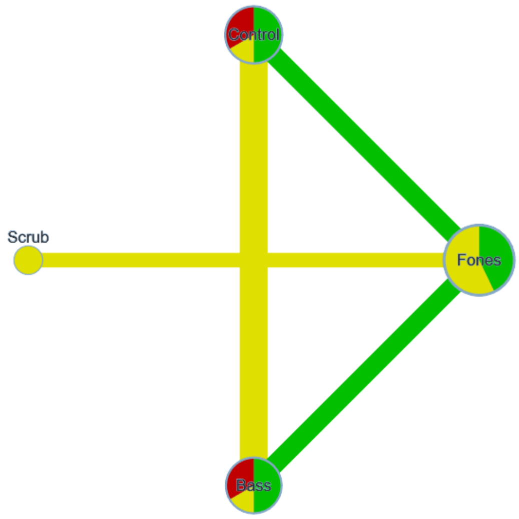

Risk of bias contributions: The bar chart shows the contributions of each piece of study to the network estimate

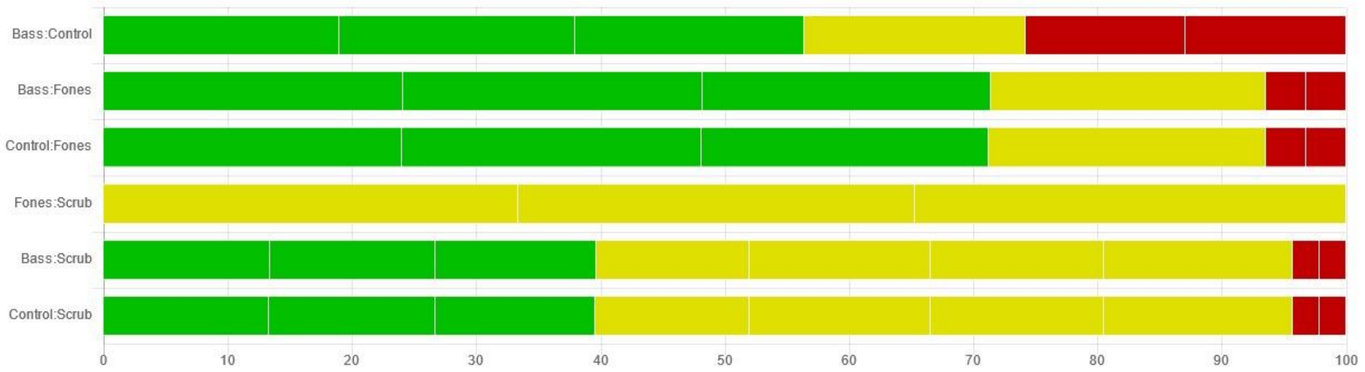

Selected Rule: *Average RoB*

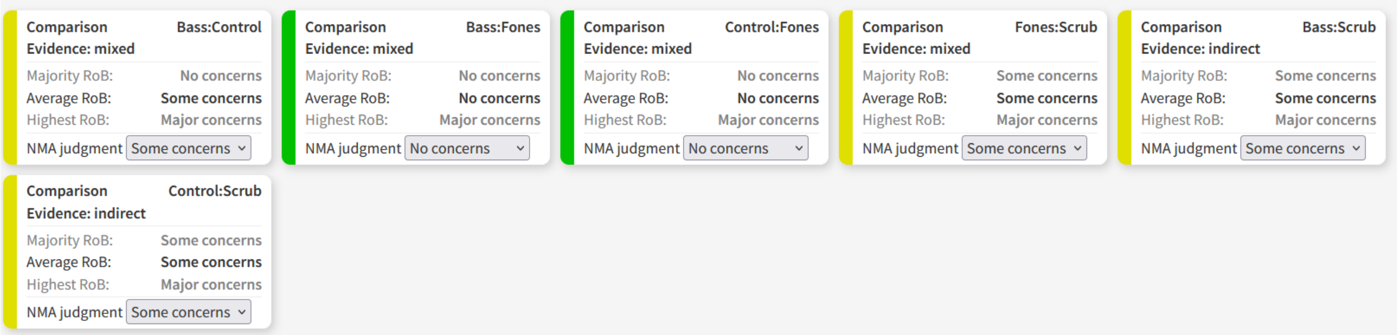

Reporting Bias

The Comparison Control:Scrub was manually set to high risk (rationale: statistics from Sarvia et al. 1989 was not available for NMA)

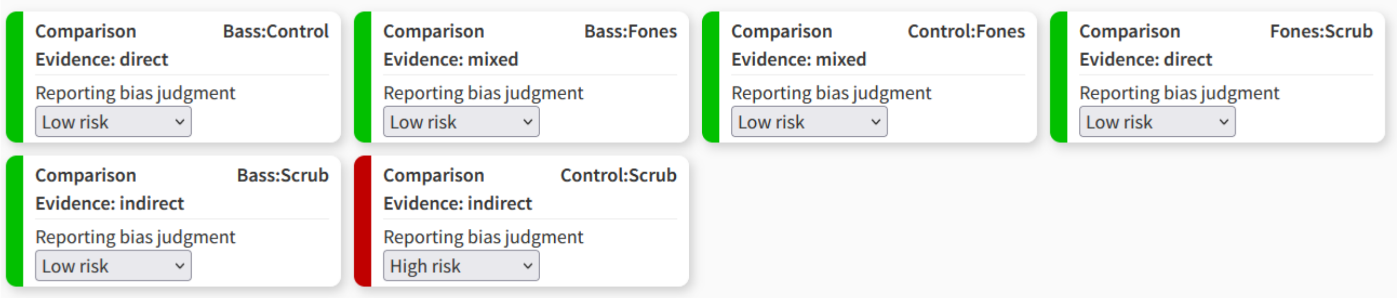

Indirectness contributions

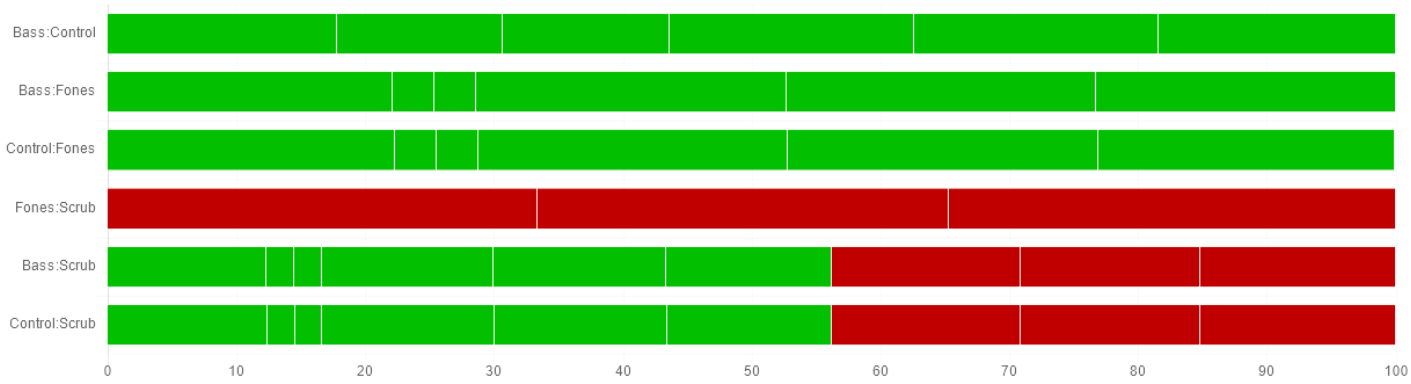

Selected rule: *Average*; indirect comparisons were manually set at high risk

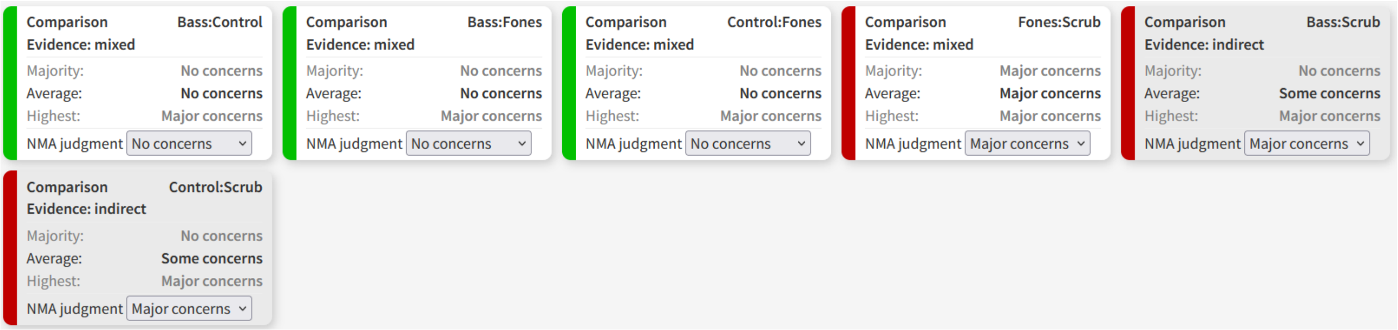

Imprecision: Clinically important effect size set at 0.2

Comparison **Bass:Control**
Evidence: mixed
NMA estimate: -0.196
95% Confidence interval:
Confidence interval **(-0.974,0.582)**
*extends into clinically important effects in **both** directions*
Imprecision judgment
Major concerns

Comparison **Bass:Fones**
Evidence: mixed
NMA estimate: 0.237
95% Confidence interval:
Confidence interval **(-0.676,1.151)**
*extends into clinically important effects in **both** directions*
Imprecision judgment
Major concerns

Comparison **Control:Fones**
Evidence: mixed
NMA estimate: 0.433
95% Confidence interval:
Confidence interval **(-0.480,1.347)**
*extends into clinically important effects in **both** directions*
Imprecision judgment
Major concerns

Comparison **Fones:Scrub**
Evidence: mixed
NMA estimate: -0.386
95% Confidence interval:
Confidence interval **(-1.515,0.743)**
*extends into clinically important effects in **both** directions*
Imprecision judgment
Major concerns

Comparison **Bass:Scrub**
Evidence: indirect
NMA estimate: -0.149
95% Confidence interval:
Confidence interval **(-1.601,1.303)**
*extends into clinically important effects in **both** directions*
Imprecision judgment
Major concerns

Comparison **Control:Scrub**
Evidence: indirect
NMA estimate: 0.047
95% Confidence interval:
Confidence interval **(-1.405,1.499)**
*extends into clinically important effects in **both** directions*
Imprecision judgment
Major concerns

Heterogeneity

The estimated value of between-study variance for the network meta-analysis is **0.865**

Comparison **Bass:Control**
Evidence: mixed
NMA estimate: -0.196
95% intervals for NMA estimate
Confidence interval: (-0.974,0.582)
Prediction interval: (-2.483,2.091)
*Confidence and prediction intervals **agree** in relation to clinically important effect*
Heterogeneity judgment
No concerns

Comparison **Bass:Fones**
Evidence: mixed
NMA estimate: 0.237
95% intervals for NMA estimate
Confidence interval: (-0.676,1.151)
Prediction interval: (-2.116,2.590)
*Confidence and prediction intervals **agree** in relation to clinically important effect*
Heterogeneity judgment
No concerns

Comparison **Control:Fones**
Evidence: mixed
NMA estimate: 0.433
95% intervals for NMA estimate
Confidence interval: (-0.480,1.347)
Prediction interval: (-1.920,2.786)
*Confidence and prediction intervals **agree** in relation to clinically important effect*
Heterogeneity judgment
No concerns

Comparison **Fones:Scrub**
Evidence: mixed
NMA estimate: -0.386
95% intervals for NMA estimate
Confidence interval: (-1.515,0.743)
Prediction interval: (-2.860,2.088)
*Confidence and prediction intervals **agree** in relation to clinically important effect*
Heterogeneity judgment
No concerns

Comparison **Bass:Scrub**
Evidence: indirect
NMA estimate: -0.149
95% intervals for NMA estimate
Confidence interval: (-1.601,1.303)
Prediction interval: (-2.838,2.541)
*Confidence and prediction intervals **agree** in relation to clinically important effect*
Heterogeneity judgment
No concerns

Comparison **Control:Scrub**
Evidence: indirect
NMA estimate: 0.047
95% intervals for NMA estimate
Confidence interval: (-1.405,1.499)
Prediction interval: (-2.643,2.736)
*Confidence and prediction intervals **agree** in relation to clinically important effect*
Heterogeneity judgment
No concerns

Incoherence

Global test based on a random-effects design-by-treatment interaction model

χ^2 statistic: 0.073 (1 degrees of freedom), P value: 0.787

Comparison **Bass:Control**
Evidence: direct
Direct standardised mean difference: **-0.196(-0.974,0.583)**
Inconsistency measures: Not applicable
Incoherence judgment
No concerns

Comparison **Bass:Fones**
Evidence: mixed
NMA standardised mean difference: **0.237(-0.676,1.151)**
Direct standardised mean difference: **0.277(-0.677,1.232)**
Indirect standardised mean difference: **-0.204(-3.365,2.958)**
Inconsistency measures
Difference of standardised mean differences: **0.481(-2.821,3.783)**
P value: **0.775**
Incoherence judgment
No concerns

Comparison **Control:Fones**
Evidence: mixed
NMA standardised mean difference: **0.433(-0.480,1.347)**
Direct standardised mean difference: **0.394(-0.560,1.348)**
Indirect standardised mean difference: **0.864(-2.299,4.026)**
Inconsistency measures
Difference of standardised mean differences: **-0.470(-3.773,2.833)**
P value: **0.780**
Incoherence judgment
No concerns

Comparison **Fones:Scrub**
Evidence: direct
Direct standardised mean difference: **-0.386(-1.515,0.743)**
Inconsistency measures: Not applicable
Incoherence judgment
No concerns

Comparison **Bass:Scrub**
Evidence: indirect
Indirect standardised mean difference: **-0.149(-1.601,1.303)**
Inconsistency measures: Not applicable
Incoherence judgment
No concerns

Comparison **Control:Scrub**
Evidence: indirect
Indirect standardised mean difference: **0.047(-1.405,1.499)**
Inconsistency measures: Not applicable
Incoherence judgment
No concerns

Overall rating of the confidence into the evidence

Comparison	Number of Studies	Within-study bias	Reporting bias	Indirectness	Imprecision	Heterogeneity	Incoherence	Confidence rating	Reason(s) for downgrading
				Mixed evidence					
**Bass vs Control**	6	Some concerns ☑	Low risk	No concerns	Major concerns ☑	No concerns	No concerns	Very low	| Within-study bias | Imprecision |
**Bass vs Fones**	4	No concerns	Low risk	No concerns	Major concerns ☑	No concerns	No concerns	Low	| Imprecision |
**Control vs Fones**	4	No concerns	Low risk	No concerns	Major concerns ☑	No concerns	No concerns	Low	| Imprecision |
**Fones vs Scrub**	3	Some concerns ☑	Low risk	Major concerns ☑	Major concerns ☑	No concerns	No concerns	Very low	| Within-study bias | Indirectness | Imprecision |
				Indirect evidence					
**Bass vs Scrub**	--	Some concerns ☑	Low risk	Major concerns ☑	Major concerns ☑	No concerns	No concerns	Very low	| Within-study bias | Indirectness | Imprecision |
**Control vs Scrub**	--	Some concerns ☑	High risk ☑	Major concerns ☑	Major concerns ☑	No concerns	No concerns	Very low	| Within-study bias | Reporting bias | Indirectness | Imprecision |

3. CINeMA-Output: Secondary Plaque (studies with adults only)

	id	study	year	feature	outcome	index	t1	n1	y1	sd1	rob	indirectness	t2	n2	y2	sd2
1	2	Harnacke et al. (2012)	2012	plaque	secondary	MPI	Control	19	62.51	20.05	2	1	Fones	18	50.19	15.33
2	2	Harnacke et al. (2012)	2012	plaque	secondary	MPI	Control	19	62.51	20.05	2	1	Bass	18	64.72	19.31
3	2	Harnacke et al. (2012)	2012	plaque	secondary	MPI	Fones	18	50.19	15.33	2	1	Bass	18	64.72	19.31
4	3	Schlueter et al. (2013.1)	2013	plaque	secondary	TQHI	Control	27	1.8	0.47	3	1	Bass	24	1.58	0.58
5	4	Schlueter et al. (2013.2)	2013	plaque	secondary	TQHI	Control	27	1.8	0.47	3	1	Bass	26	1.64	0.58
6	5	Deinzer et al. (2016.1)	2016	plaque	secondary	MPI	Control	30	76.18	14.93	1	1	Fones	32	71.99	14.92
7	5	Deinzer et al. (2016.1)	2016	plaque	secondary	MPI	Control	30	76.18	14.93	1	1	Bass	30	70.56	16.9
8	5	Deinzer et al. (2016.1)	2016	plaque	secondary	MPI	Fones	32	71.99	14.92	1	1	Bass	30	70.56	16.9
9	6	Deinzer et al. (2016.2)	2016	plaque	secondary	MPI	Control	30	42.18	25.56	1	1	Fones	32	36.78	23.99
10	6	Deinzer et al. (2016.2)	2016	plaque	secondary	MPI	Control	30	42.18	25.56	1	1	Bass	30	39.24	23.41
11	6	Deinzer et al. (2016.2)	2016	plaque	secondary	MPI	Fones	32	36.78	23.99	1	1	Bass	30	39.24	23.41
12	7	Harnacke et al. (2016)	2016	plaque	secondary	MPI	Control	22	77.45	12.08	1	1	Fones	23	70.67	14.49
13	7	Harnacke et al. (2016)	2016	plaque	secondary	MPI	Control	22	77.45	12.08	1	1	Bass	23	76.05	17.26
14	7	Harnacke et al. (2016)	2016	plaque	secondary	MPI	Fones	23	70.67	14.49	1	1	Bass	23	76.05	17.26

Node size by: Sample size **Node color by:** Risk of Bias **Edge width by:** Sample Size **Edge color by:** Average RoB

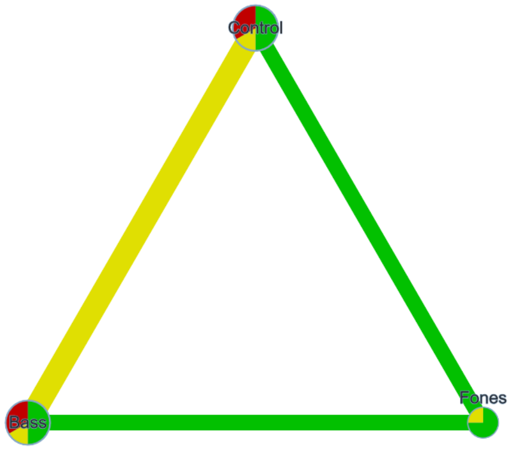

Risk of bias contributions: The bar chart shows the contributions of each piece of study to the network estimate

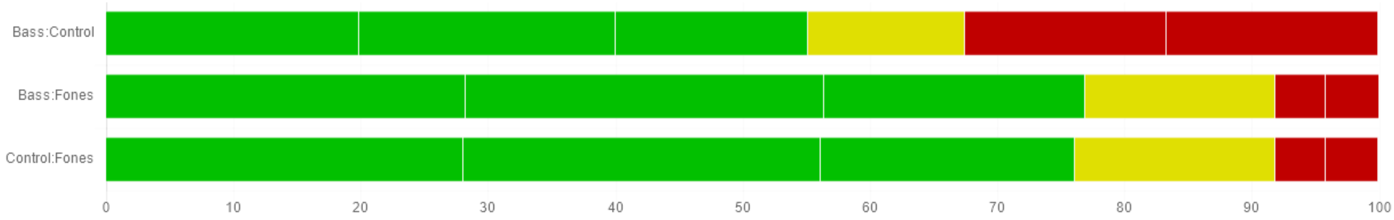

Selected Rule: *Average RoB*

Comparison	Bass:Control
Evidence: mixed	
Majority RoB:	No concerns
Average RoB:	Some concerns
Highest RoB:	Major concerns
NMA judgment	Some concerns

Comparison	Bass:Fones
Evidence: mixed	
Majority RoB:	No concerns
Average RoB:	No concerns
Highest RoB:	Major concerns
NMA judgment	No concerns

Comparison	Control:Fones
Evidence: mixed	
Majority RoB:	No concerns
Average RoB:	No concerns
Highest RoB:	Major concerns
NMA judgment	No concerns

Reporting Bias: Judgement „all low Risk“

Indirectness: Judgement “all no concerns”

Imprecision: Clinically important effect size set at 0.2

Comparison	Bass:Control	Comparison	Bass:Fones	Comparison	Control:Fones
**Evidence: mixed**		**Evidence: mixed**		**Evidence: mixed**	
NMA estimate:	**-0.213**	NMA estimate:	**0.174**	NMA estimate:	**0.387**
95% Confidence interval:		95% Confidence interval:		95% Confidence interval:	
Confidence interval	**(-0.439,0.012)**	*Confidence interval*	**(-0.090,0.438)**	*Confidence interval*	**(0.123,0.651)**
extends into clinically important effects		*extends into clinically important effects*		*does not cross clinically important effect*	
Imprecision judgment	Some concerns	Imprecision judgment	Some concerns	Imprecision judgment	No concerns

Heterogeneity

The estimated value of between-study variance for the network meta-analysis is **0.000**

Comparison	Bass:Control	Comparison	Bass:Fones	Comparison	Control:Fones
**Evidence: mixed**		**Evidence: mixed**		**Evidence: mixed**	
NMA estimate:	**-0.213**	NMA estimate:	**0.174**	NMA estimate:	**0.387**
**95% intervals for NMA estimate**		**95% intervals for NMA estimate**		**95% intervals for NMA estimate**	
Confidence interval:	**(-0.439,0.012)**	Confidence interval:	**(-0.090,0.438)**	Confidence interval:	**(0.123,0.651)**
Prediction interval:	**(-0.485,0.058)**	Prediction interval:	**(-0.144,0.492)**	Prediction interval:	**(0.069,0.706)**
*Confidence and prediction intervals **agree** in relation to clinically important effect*		*Confidence and prediction intervals **agree** in relation to clinically important effect*		*Confidence and prediction intervals **agree** in relation to clinically important effect*	
Heterogeneity judgment	No concerns	Heterogeneity judgment	No concerns	Heterogeneity judgment	No concerns

Incoherence

Global test based on a random-effects design-by-treatment interaction model

χ^2 statistic: 0.767 (1 degrees of freedom), P value: 0.381

Comparison	Bass:Control
**Evidence: direct**	
Direct standardised mean difference:	**-0.212(-0.437,0.013)**
**Inconsistency measures:** Not applicable	
Incoherence judgment	No concerns

Comparison	Bass:Fones	Comparison	Control:Fones
**Evidence: mixed**		**Evidence: mixed**	
NMA standardised mean difference:	**0.174(-0.090,0.438)**	NMA standardised mean difference:	**0.387(0.123,0.651)**
Direct standardised mean difference:	**0.210(-0.066,0.485)**	Direct standardised mean difference:	**0.359(0.083,0.635)**
Indirect standardised mean difference:	**-0.213(-1.118,0.693)**	Indirect standardised mean difference:	**0.690(-0.211,1.590)**
**Inconsistency measures**		**Inconsistency measures**	
Difference of standardised mean differences:	**0.423(-0.524,1.369)**	Difference of standardised mean differences:	**-0.331(-1.272,0.611)**
P value:	**0.382**	P value:	**0.491**
Incoherence judgment	No concerns	Incoherence judgment	No concerns

Overall rating of the confidence into the evidence

Comparison	Number of Studies	Within-study bias	Reporting bias	Indirectness	Imprecision	Heterogeneity	Incoherence	Confidence rating	Reason(s) for downgrading
Mixed evidence									
Bass vs Control	6	Some concerns ☑	Low risk	No concerns	Some concerns ☑	No concerns	No concerns	Low	| Within-study bias | Imprecision |
Bass vs Fones	4	No concerns	Low risk	No concerns	Some concerns ☑	No concerns	No concerns	Moderate	| Imprecision |
Control vs Fones	4	No concerns	Low risk	No concerns	No concerns	No concerns	No concerns	High	|

4. CINeMA-Output: Primary Gingivitis (all studies)

	study	year	feature	outcome	index	t1	n1	y1	sd1	rob	indirectness	t2	n2	y2	sd2
1	Smutkeeree et al. (2011)	2011	gingivitis	primary	GI	Scrub	29	2.42	0.28	3	3	Bass	28	2.43	0.34
2	Harnacke et al. (2012)	2012	gingivitis	primary	PBI	Control	19	26	15.03	2	1	Fones	19	15.35	12.57
3	Harnacke et al. (2012)	2012	gingivitis	primary	PBI	Control	19	26	15.03	2	1	Bass	18	27.96	21.29
4	Harnacke et al. (2012)	2012	gingivitis	primary	PBI	Fones	19	15.35	12.57	2	1	Bass	18	27.96	21.29
5	Harnacke et al. (2016)	2016	gingivitis	primary	BOP	Control	22	7.36	4.7	1	1	Fones	23	10.5	7.39
6	Harnacke et al. (2016)	2016	gingivitis	primary	BOP	Control	22	7.36	4.7	1	1	Bass	24	10.84	7.01
7	Harnacke et al. (2016)	2016	gingivitis	primary	BOP	Fones	23	10.5	7.39	1	1	Bass	24	10.84	7.01
8	Schmalz et al. (2018)	2018	gingivitis	primary	GI	Control	22	1	0.08	2	2	Fones	22	0.97	0.14

Node size by: Sample size **Node color by:** Risk of Bias **Edge width by:** Sample Size **Edge color by:** Average RoB

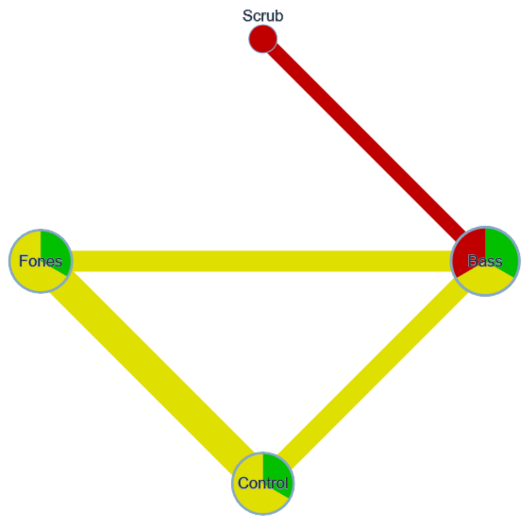

Risk of bias contributions: The bar chart shows the contributions of each piece of study to the network estimate

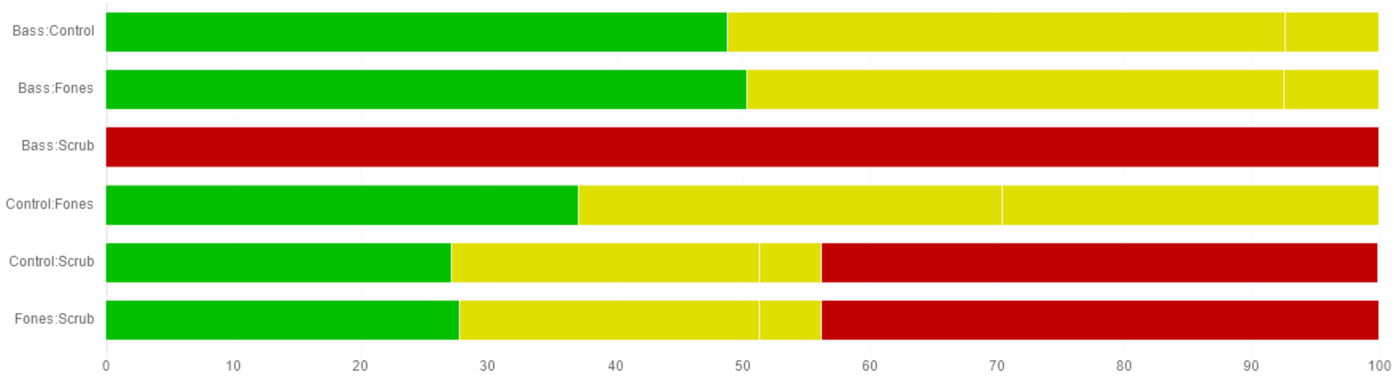

Selected Rule: *Average RoB*

Comparison	Bass:Control
Evidence: mixed	
Majority RoB:	Some concerns
Average RoB:	Some concerns
Highest RoB:	Major concerns
NMA judgment	Some concerns

Comparison	Bass:Fones
Evidence: mixed	
Majority RoB:	No concerns
Average RoB:	No concerns
Highest RoB:	Major concerns
NMA judgment	No concerns

Comparison	Bass:Scrub
Evidence: mixed	
Majority RoB:	Major concerns
Average RoB:	Major concerns
Highest RoB:	Major concerns
NMA judgment	Major concerns

Comparison	Control:Fones
Evidence: mixed	
Majority RoB:	Some concerns
Average RoB:	Some concerns
Highest RoB:	Major concerns
NMA judgment	Some concerns

Comparison	Control:Scrub
Evidence: indirect	
Majority RoB:	Major concerns
Average RoB:	Some concerns
Highest RoB:	Major concerns
NMA judgment	Some concerns

Comparison	Fones:Scrub
Evidence: indirect	
Majority RoB:	Major concerns
Average RoB:	Some concerns
Highest RoB:	Major concerns
NMA judgment	Some concerns

Reporting Bias: The Comparison Bass:Control was manually set to high risk (rationale: statistics from Dosumo et al. 2019 and Ausenda et al. 2019 were not available for NMA)

Comparison	Evidence	Reporting bias judgment
Bass:Control	mixed	High risk
Bass:Fones	mixed	Low risk
Bass:Scrub	direct	Low risk
Control:Fones	direct	Low risk
Control:Scrub	indirect	Low risk
Fones:Scrub	indirect	Low risk

Indirectness contributions

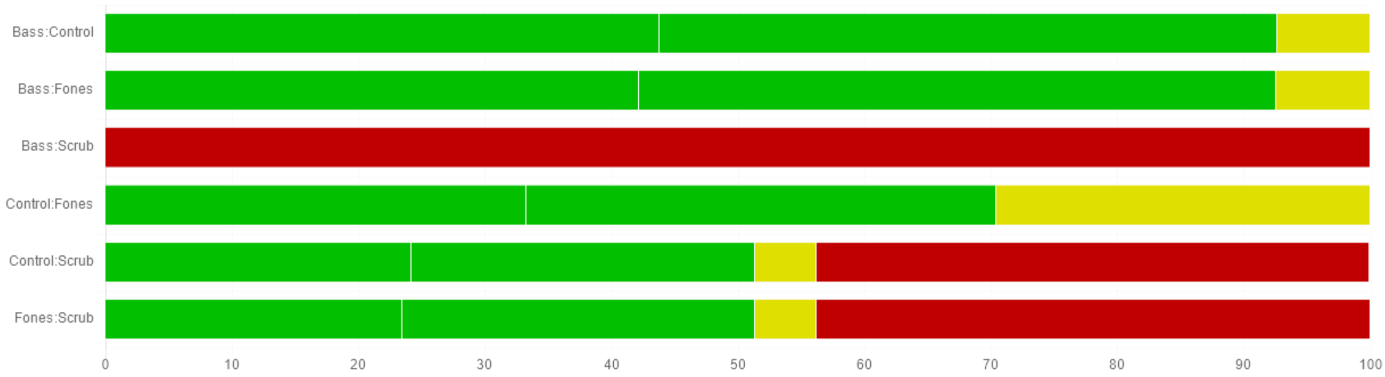

Selected rule: *Average*; indirect comparisons were manually set at high risk

Comparison	Evidence	Majority	Average	Highest	NMA judgment
Bass:Control	mixed	No concerns	No concerns	Major concerns	No concerns
Bass:Fones	mixed	No concerns	No concerns	Major concerns	No concerns
Bass:Scrub	mixed	Major concerns	Major concerns	Major concerns	Major concerns
Control:Fones	mixed	No concerns	No concerns	Major concerns	No concerns
Control:Scrub	indirect	No concerns	Some concerns	Major concerns	Major concerns
Fones:Scrub	indirect	No concerns	Some concerns	Major concerns	Major concerns

Imprecision: Clinically important effect size set at 0.2

Comparison	Evidence	NMA estimate	95% Confidence interval		Imprecision judgment
Bass:Control	mixed	0.285	(-0.327,0.898)	*Confidence interval extends into clinically important effects in **both** directions*	Major concerns
Bass:Fones	mixed	0.407	(-0.206,1.021)	*Confidence interval extends into clinically important effects in **both** directions*	Major concerns
Bass:Scrub	mixed	0.032	(-0.811,0.875)	*Confidence interval extends into clinically important effects in **both** directions*	Major concerns
Control:Fones	mixed	0.122	(-0.400,0.644)	*Confidence interval extends into clinically important effects in **both** directions*	Major concerns
Control:Scrub	indirect	-0.254	(-1.296,0.789)	*Confidence interval extends into clinically important effects in **both** directions*	Major concerns
Fones:Scrub	indirect	-0.376	(-1.418,0.667)	*Confidence interval extends into clinically important effects in **both** directions*	Major concerns

Heterogeneity

The estimated value of between-study variance for the network meta-analysis is **0.115**

Comparison **Bass:Control**
Evidence: mixed
NMA estimate: **0.285**
95% intervals for NMA estimate
Confidence interval: **(-0.327,0.898)**
Prediction interval: **(-1.698,2.269)**
Confidence and prediction intervals ***agree*** *in relation to clinically important effect*
Heterogeneity judgment: No concerns

Comparison **Bass:Fones**
Evidence: mixed
NMA estimate: **0.407**
95% intervals for NMA estimate
Confidence interval: **(-0.206,1.021)**
Prediction interval: **(-1.578,2.392)**
Confidence and prediction intervals ***agree*** *in relation to clinically important effect*
Heterogeneity judgment: No concerns

Comparison **Bass:Scrub**
Evidence: mixed
NMA estimate: **0.032**
95% intervals for NMA estimate
Confidence interval: **(-0.811,0.875)**
Prediction interval: **(-2.324,2.388)**
Confidence and prediction intervals ***agree*** *in relation to clinically important effect*
Heterogeneity judgment: No concerns

Comparison **Control:Fones**
Evidence: mixed
NMA estimate: **0.122**
95% intervals for NMA estimate
Confidence interval: **(-0.400,0.644)**
Prediction interval: **(-1.732,1.976)**
Confidence and prediction intervals ***agree*** *in relation to clinically important effect*
Heterogeneity judgment: No concerns

Comparison **Control:Scrub**
Evidence: indirect
NMA estimate: **-0.254**
95% intervals for NMA estimate
Confidence interval: **(-1.296,0.789)**
Prediction interval: **(-2.966,2.459)**
Confidence and prediction intervals ***agree*** *in relation to clinically important effect*
Heterogeneity judgment: No concerns

Comparison **Fones:Scrub**
Evidence: indirect
NMA estimate: **-0.376**
95% intervals for NMA estimate
Confidence interval: **(-1.418,0.667)**
Prediction interval: **(-3.089,2.338)**
Confidence and prediction intervals ***agree*** *in relation to clinically important effect*
Heterogeneity judgment: No concerns

Incoherence

Global test based on a random-effects design-by-treatment interaction model

χ^2 **statistic: 0.089 (1 degrees of freedom), P value: 0.766**

Comparison **Bass:Control**
Evidence: mixed
NMA standardised mean difference: **0.285(-0.327,0.898)**
Direct standardised mean difference: **0.329(-0.312,0.970)**
Indirect standardised mean difference: **-0.180(-2.268,1.907)**
Inconsistency measures
Difference of standardised mean differences: **0.509(-1.674,2.693)**
P value: **0.648**
Incoherence judgment: No concerns

Comparison **Bass:Fones**
Evidence: mixed
NMA standardised mean difference: **0.407(-0.206,1.021)**
Direct standardised mean difference: **0.372(-0.270,1.014)**
Indirect standardised mean difference: **0.777(-1.305,2.859)**
Inconsistency measures
Difference of standardised mean differences: **-0.405(-2.584,1.774)**
P value: **0.715**
Incoherence judgment: No concerns

Comparison **Bass:Scrub**
Evidence: direct
Direct standardised mean difference: **0.032(-0.811,0.875)**
Inconsistency measures: Not applicable
Incoherence judgment: No concerns

Comparison **Control:Fones**
Evidence: direct
Direct standardised mean difference: **0.123(-0.399,0.645)**
Inconsistency measures: Not applicable
Incoherence judgment: No concerns

Comparison **Control:Scrub**
Evidence: indirect
Indirect standardised mean difference: **-0.254(-1.296,0.789)**
Inconsistency measures: Not applicable
Incoherence judgment: No concerns

Comparison **Fones:Scrub**
Evidence: indirect
Indirect standardised mean difference: **-0.376(-1.418,0.667)**
Inconsistency measures: Not applicable
Incoherence judgment: No concerns

Overall rating of the confidence into the evidence

Comparison	Number of Studies	Within-study bias	Reporting bias	Indirectness	Imprecision	Heterogeneity	Incoherence	Confidence rating	Reason(s) for downgrading
				Mixed evidence					
Bass vs Control	2	Some concerns ☑	High risk ☑	No concerns	Major concerns ☑	No concerns	No concerns	Very low	| Within-study bias | Reporting bias | Imprecision |
Bass vs Fones	2	No concerns	Low risk	No concerns	Major concerns ☑	No concerns	No concerns	Low	| Imprecision |
Bass vs Scrub	1	Major concerns ☑	Low risk	Major concerns ☑	Major concerns ☑	No concerns	No concerns	Very low	| Within-study bias | Indirectness | Imprecision |
Control vs Fones	3	Some concerns ☑	Low risk	No concerns	Major concerns ☑	No concerns	No concerns	Very low	| Within-study bias | Imprecision |
				Indirect evidence					
Control vs Scrub	--	Some concerns ☑	Low risk	Major concerns ☑	Major concerns ☑	No concerns	No concerns	Very low	| Within-study bias | Indirectness | Imprecision |
Fones vs Scrub	--	Some concerns ☑	Low risk	Major concerns ☑	Major concerns ☑	No concerns	No concerns	Very low	| Within-study bias | Indirectness | Imprecision |

5. CINeMA-Output: Primary Gingivitis (studies with adults only)

	study	year	feature	outcome	index	t1	n1	y1	sd1	rob	indirectness	t2	n2	y2	sd2
1	Harnacke et al. (2012)	2012	gingivitis	primary	PBI	Control	19	26	15.03	2	1	Fones	19	15.35	12.57
2	Harnacke et al. (2012)	2012	gingivitis	primary	PBI	Control	19	26	15.03	2	1	Bass	18	27.96	21.29
3	Harnacke et al. (2012)	2012	gingivitis	primary	PBI	Fones	19	15.35	12.57	2	1	Bass	18	27.96	21.29
4	Harnacke et al. (2016)	2016	gingivitis	primary	BOP	Control	22	7.36	4.7	1	1	Fones	23	10.5	7.39
5	Harnacke et al. (2016)	2016	gingivitis	primary	BOP	Control	22	7.36	4.7	1	1	Bass	24	10.84	7.01
6	Harnacke et al. (2016)	2016	gingivitis	primary	BOP	Fones	23	10.5	7.39	1	1	Bass	24	10.84	7.01
7	Schmalz et al. (2018)	2018	gingivitis	primary	GI	Control	22	1	0.08	2	2	Fones	22	0.97	0.14

Node size by: Sample size **Node color by:** Risk of Bias **Edge width by:** Sample Size **Edge color by:** Average RoB

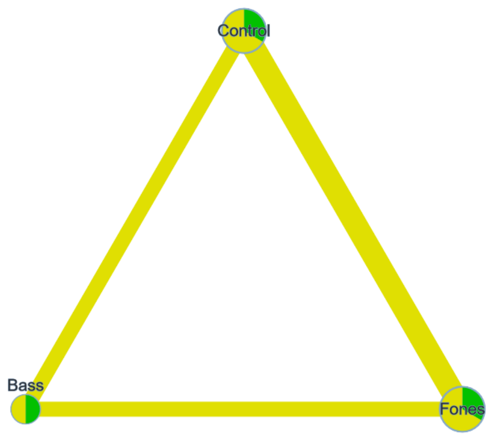

Risk of bias contributions: The bar chart shows the contributions of each piece of study to the network estimate

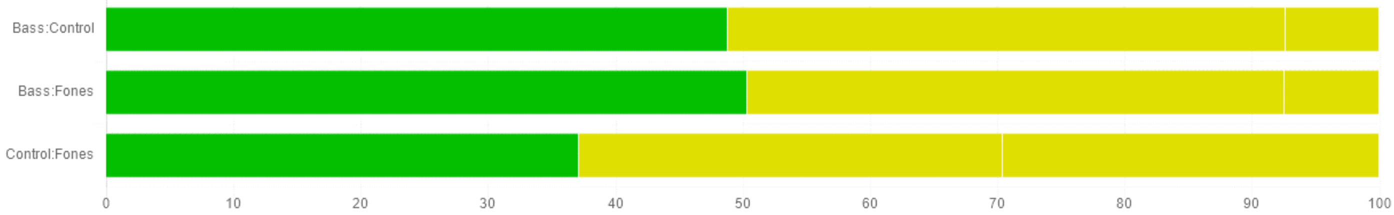

Selected Rule: *Average RoB*

Comparison **Bass:Control**
Evidence: mixed
Majority RoB: Some concerns
Average RoB: **Some concerns**
Highest RoB: Some concerns
NMA judgment Some concerns

Comparison **Bass:Fones**
Evidence: mixed
Majority RoB: No concerns
Average RoB: **No concerns**
Highest RoB: Some concerns
NMA judgment No concerns

Comparison **Control:Fones**
Evidence: mixed
Majority RoB: Some concerns
Average RoB: **Some concerns**
Highest RoB: Some concerns
NMA judgment Some concerns

Reporting Bias: The Comparison Bass:Control was manually set to high risk (rationale: statistics from Dosumo et al. 2019 and Ausenda et al. 2019 were not available for NMA)

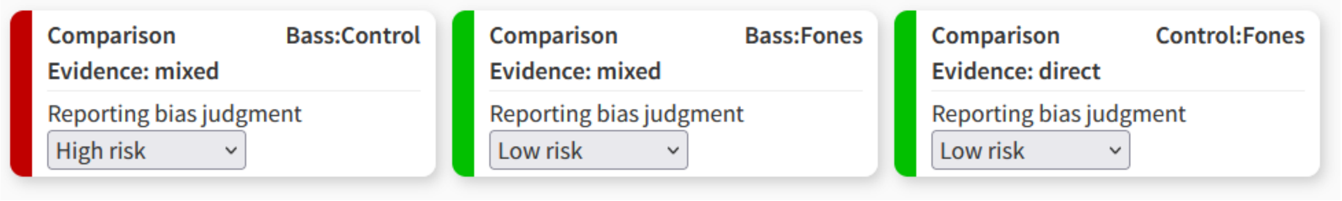

Indirectness contributions

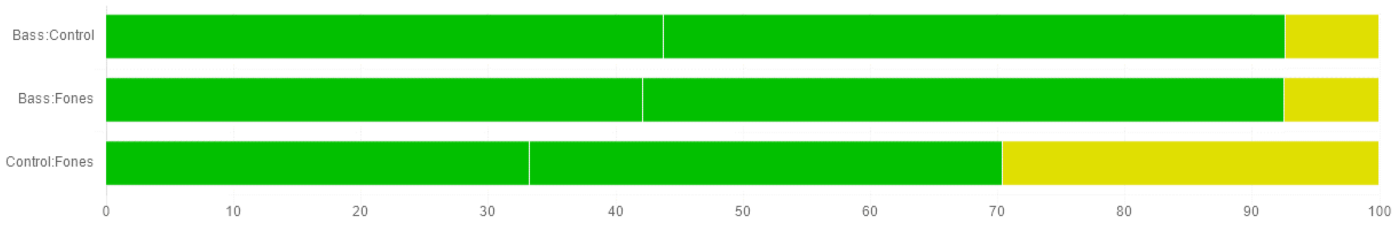

Selected rule: *Average*

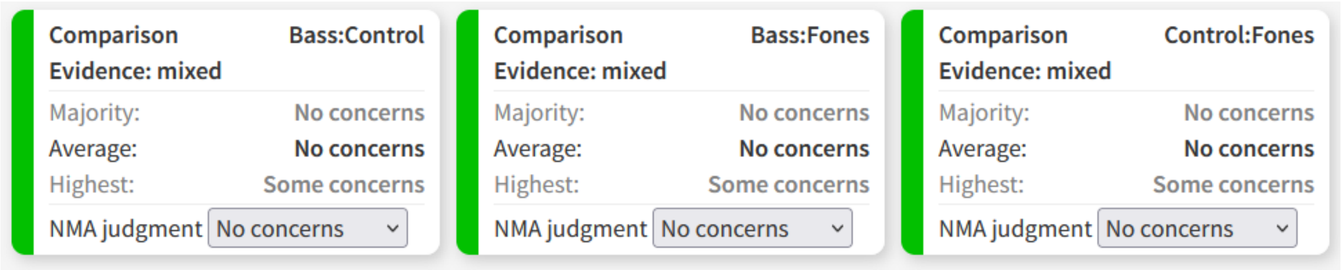

Imprecision: Clinically important effect size set at 0.2

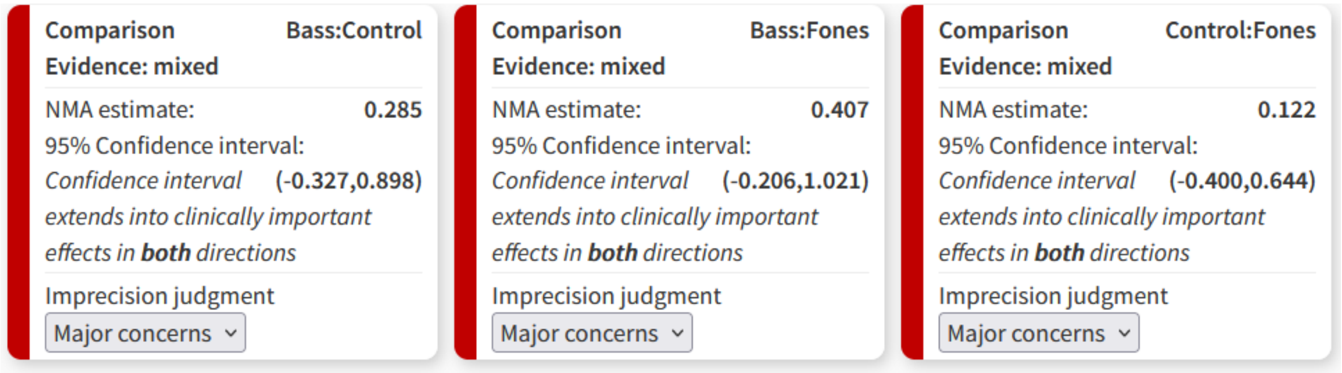

Heterogeneity

The estimated value of between-study variance for the network meta-analysis is **0.115**

Comparison **Bass:Control**
Evidence: mixed
NMA estimate: **0.285**
95% intervals for NMA estimate
Confidence interval: **(-0.327,0.898)**
Prediction interval: **(-1.698,2.269)**
*Confidence and prediction intervals **agree** in relation to clinically important effect*
Heterogeneity judgment
No concerns

Comparison **Bass:Fones**
Evidence: mixed
NMA estimate: **0.407**
95% intervals for NMA estimate
Confidence interval: **(-0.206,1.021)**
Prediction interval: **(-1.578,2.392)**
*Confidence and prediction intervals **agree** in relation to clinically important effect*
Heterogeneity judgment
No concerns

Comparison **Control:Fones**
Evidence: mixed
NMA estimate: **0.122**
95% intervals for NMA estimate
Confidence interval: **(-0.400,0.644)**
Prediction interval: **(-1.732,1.976)**
*Confidence and prediction intervals **agree** in relation to clinically important effect*
Heterogeneity judgment
No concerns

Incoherence

Global test based on a random-effects design-by-treatment interaction model

χ^2 statistic: 0.089 (1 degrees of freedom), P value: 0.766

Comparison **Bass:Control**
Evidence: mixed
NMA standardised mean difference: **0.285(-0.327,0.898)**
Direct standardised mean difference: **0.329(-0.312,0.970)**
Indirect standardised mean difference: **-0.180(-2.268,1.907)**
Inconsistency measures
Difference of standardised mean differences: **0.509(-1.674,2.693)**
P value: **0.648**
Incoherence judgment: No concerns

Comparison **Bass:Fones**
Evidence: mixed
NMA standardised mean difference: **0.407(-0.206,1.021)**
Direct standardised mean difference: **0.372(-0.270,1.014)**
Indirect standardised mean difference: **0.777(-1.305,2.859)**
Inconsistency measures
Difference of standardised mean differences: **-0.405(-2.584,1.774)**
P value: **0.715**
Incoherence judgment: No concerns

Comparison **Control:Fones**
Evidence: direct
Direct standardised mean difference: **0.123(-0.399,0.645)**
Inconsistency measures: Not applicable
Incoherence judgment: No concerns

Overall rating of the confidence into the evidence

<table>
<tr><th>Comparison</th><th>Number of Studies</th><th>Within-study bias</th><th>Reporting bias</th><th>Indirectness</th><th>Imprecision</th><th>Heterogeneity</th><th>Incoherence</th><th>Confidence rating</th><th>Reason(s) for downgrading</th></tr>
<tr><td colspan="10">Mixed evidence</td></tr>
<tr><td>Bass vs Control</td><td>2</td><td>Some concerns ☑</td><td>High risk ☑</td><td>No concerns</td><td>Major concerns ☑</td><td>No concerns</td><td>No concerns</td><td>Very low</td><td>| Within-study bias | Reporting bias | Imprecision |</td></tr>
<tr><td>Bass vs Fones</td><td>2</td><td>No concerns</td><td>Low risk</td><td>No concerns</td><td>Major concerns ☑</td><td>No concerns</td><td>No concerns</td><td>Low</td><td>| Imprecision |</td></tr>
<tr><td>Control vs Fones</td><td>3</td><td>Some concerns ☑</td><td>Low risk</td><td>No concerns</td><td>Major concerns ☑</td><td>No concerns</td><td>No concerns</td><td>Very low</td><td>| Within-study bias | Imprecision |</td></tr>
</table>

6. CINeMA-Output: Secondary Gingivitis (all studies)

	study	year	feature	outcome	index	t1	n1	y1	sd1	rob	indirectness	t2	n2	y2	sd2
1	Smutkeeree et al. (2011)	2011	gingivitis	secondary	GI	Scrub	29	2.35	0.18	3	3	Bass	28	2.4	0.27
2	Harnacke et al. (2012)	2012	gingivitis	secondary	PBI	Control	19	17.58	14.74	2	1	Fones	18	22.23	25.56
3	Harnacke et al. (2012)	2012	gingivitis	secondary	PBI	Control	19	17.58	14.74	2	1	Bass	19	18.57	12.67
4	Harnacke et al. (2012)	2012	gingivitis	secondary	PBI	Fones	18	22.23	25.56	2	1	Bass	19	18.57	12.67
5	Harnacke et al. (2016)	2016	gingivitis	secondary	BOP	Control	22	11.79	6.65	1	1	Fones	23	10.5	6.91
6	Harnacke et al. (2016)	2016	gingivitis	secondary	BOP	Control	22	11.79	6.65	1	1	Bass	24	11.15	8.4
7	Harnacke et al. (2016)	2016	gingivitis	secondary	BOP	Fones	23	10.5	6.91	1	1	Bass	24	11.15	8.4
8	Schmalz et al. (2018)	2018	gingivitis	secondary	GI	Control	22	1.03	0.11	2	2	Fones	22	0.96	0.11
9	Janakiram et al. (2020)	2020	gingivitis	secondary	GI	Control	40	0.9	0.61	2	2	Fones	40	1.1	0.5
10	Janakiram et al. (2020)	2020	gingivitis	secondary	GI	Control	40	0.9	0.61	2	2	Bass	40	0.9	0.4
11	Janakiram et al. (2020)	2020	gingivitis	secondary	GI	Fones	40	1.1	0.5	2	2	Bass	40	0.9	0.4

Node size by: Sample size **Node color by:** Risk of Bias **Edge width by:** Sample Size **Edge color by:** Average RoB

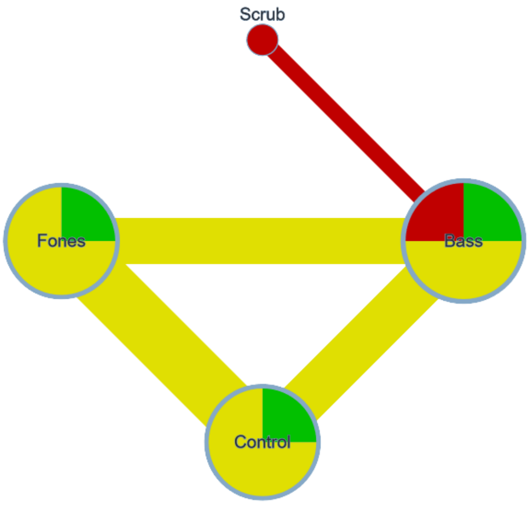

Risk of bias contributions: The bar chart shows the contributions of each piece of study to the network estimate

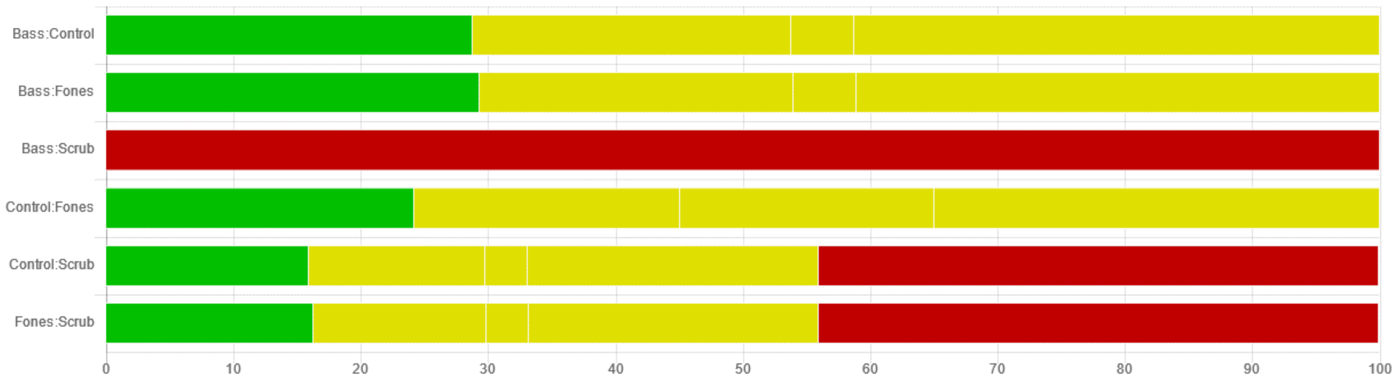

Selected Rule: *Average RoB*

Comparison	Bass:Control
Evidence: mixed	
Majority RoB:	Some concerns
Average RoB:	Some concerns
Highest RoB:	Major concerns
NMA judgment	Some concerns

Comparison	Bass:Fones
Evidence: mixed	
Majority RoB:	Some concerns
Average RoB:	Some concerns
Highest RoB:	Major concerns
NMA judgment	Some concerns

Comparison	Bass:Scrub
Evidence: mixed	
Majority RoB:	Major concerns
Average RoB:	Major concerns
Highest RoB:	Major concerns
NMA judgment	Major concerns

Comparison	Control:Fones
Evidence: mixed	
Majority RoB:	Some concerns
Average RoB:	Some concerns
Highest RoB:	Major concerns
NMA judgment	Some concerns

Comparison	Control:Scrub
Evidence: indirect	
Majority RoB:	Major concerns
Average RoB:	Some concerns
Highest RoB:	Major concerns
NMA judgment	Some concerns

Comparison	Fones:Scrub
Evidence: indirect	
Majority RoB:	Major concerns
Average RoB:	Some concerns
Highest RoB:	Major concerns
NMA judgment	Some concerns

Reporting Bias: The Comparison Bass:Control was manually set to high risk (rationale: statistics from Dosumo et al. 2019 and Ausenda et al. 2019 were not available for NMA)

Comparison	Evidence	Reporting bias judgment
Bass:Control	mixed	High risk
Bass:Fones	mixed	Low risk
Bass:Scrub	direct	Low risk
Control:Fones	direct	Low risk
Control:Scrub	indirect	Low risk
Fones:Scrub	indirect	Low risk

Indirectness contributions

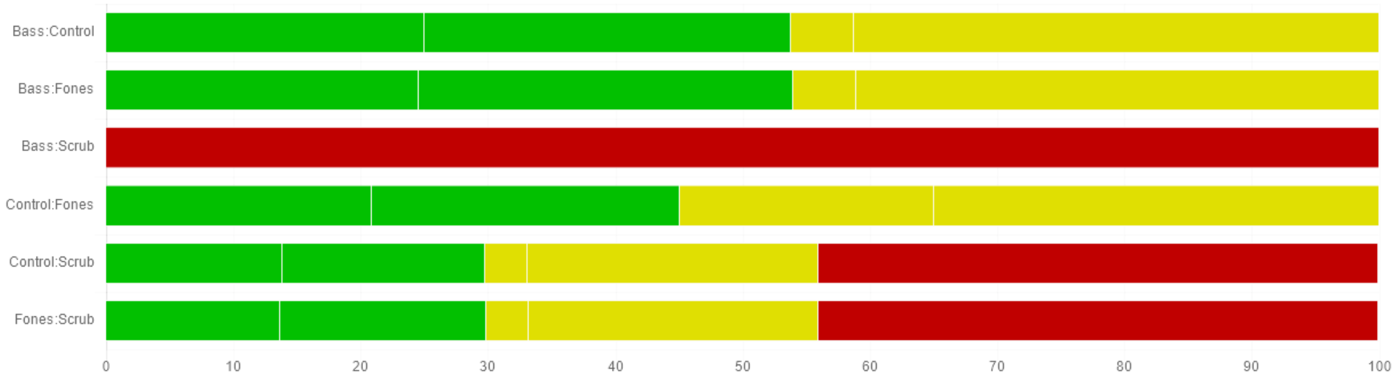

Selected rule: *Average*; indirect comparisons were manually set at high risk

Comparison	Evidence	Majority	Average	Highest	NMA judgment
Bass:Control	mixed	No concerns	No concerns	Major concerns	No concerns
Bass:Fones	mixed	No concerns	No concerns	Major concerns	No concerns
Bass:Scrub	mixed	Major concerns	Major concerns	Major concerns	Major concerns
Control:Fones	mixed	Some concerns	Some concerns	Major concerns	Some concerns
Control:Scrub	indirect	Major concerns	Some concerns	Major concerns	Major concerns
Fones:Scrub	indirect	Major concerns	Some concerns	Major concerns	Major concerns

Imprecision: Clinically important effect size set at 0.2

Comparison	Evidence	NMA estimate	95% Confidence interval		Imprecision judgment
Bass:Control	mixed	-0.101	(-0.495,0.294)	*Confidence interval extends into clinically important effects in **both** directions*	Major concerns
Bass:Fones	mixed	-0.096	(-0.491,0.300)	*Confidence interval extends into clinically important effects in **both** directions*	Major concerns
Bass:Scrub	mixed	0.216	(-0.472,0.903)	*Confidence interval extends into clinically important effects in **both** directions*	Major concerns
Control:Fones	mixed	0.005	(-0.355,0.365)	*Confidence interval extends into clinically important effects in **both** directions*	Major concerns
Control:Scrub	indirect	0.316	(-0.476,1.109)	*Confidence interval extends into clinically important effects in **both** directions*	Major concerns
Fones:Scrub	indirect	0.311	(-0.482,1.105)	*Confidence interval extends into clinically important effects in **both** directions*	Major concerns

Heterogeneity

The estimated value of between-study variance for the network meta-analysis is **0.052**

Comparison Bass:Control
Evidence: mixed
NMA estimate: -0.101
95% intervals for NMA estimate
Confidence interval: (-0.495,0.294)
Prediction interval: (-0.948,0.746)
*Confidence and prediction intervals **agree** in relation to clinically important effect*
Heterogeneity judgment: No concerns

Comparison Bass:Fones
Evidence: mixed
NMA estimate: -0.096
95% intervals for NMA estimate
Confidence interval: (-0.491,0.300)
Prediction interval: (-0.943,0.752)
*Confidence and prediction intervals **agree** in relation to clinically important effect*
Heterogeneity judgment: No concerns

Comparison Bass:Scrub
Evidence: mixed
NMA estimate: 0.216
95% intervals for NMA estimate
Confidence interval: (-0.472,0.903)
Prediction interval: (-0.948,1.379)
*Confidence and prediction intervals **agree** in relation to clinically important effect*
Heterogeneity judgment: No concerns

Comparison Control:Fones
Evidence: mixed
NMA estimate: 0.005
95% intervals for NMA estimate
Confidence interval: (-0.355,0.365)
Prediction interval: (-0.810,0.820)
*Confidence and prediction intervals **agree** in relation to clinically important effect*
Heterogeneity judgment: No concerns

Comparison Control:Scrub
Evidence: indirect
NMA estimate: 0.316
95% intervals for NMA estimate
Confidence interval: (-0.476,1.109)
Prediction interval: (-0.974,1.607)
*Confidence and prediction intervals **agree** in relation to clinically important effect*
Heterogeneity judgment: No concerns

Comparison Fones:Scrub
Evidence: indirect
NMA estimate: 0.311
95% intervals for NMA estimate
Confidence interval: (-0.482,1.105)
Prediction interval: (-0.980,1.603)
*Confidence and prediction intervals **agree** in relation to clinically important effect*
Heterogeneity judgment: No concerns

Incoherence

Global test based on a random-effects design-by-treatment interaction model

χ^2 statistic: 5.608 (1 degrees of freedom), P value: 0.018

Comparison Bass:Control
Evidence: mixed
NMA standardised mean difference: -0.101(-0.495,0.294)
Direct standardised mean difference: -0.012(-0.418,0.395)
Indirect standardised mean difference: -1.567(-3.218,0.084)
Inconsistency measures
Difference of standardised mean differences: 1.555(-0.145,3.255)
P value: 0.073
Incoherence judgment: Some concerns

Comparison Bass:Fones
Evidence: mixed
NMA standardised mean difference: -0.096(-0.491,0.300)
Direct standardised mean difference: -0.187(-0.595,0.220)
Indirect standardised mean difference: 1.392(-0.250,3.034)
Inconsistency measures
Difference of standardised mean differences: -1.579(-3.271,0.113)
P value: 0.067
Incoherence judgment: No concerns

Comparison Bass:Scrub
Evidence: direct
Direct standardised mean difference: 0.216(-0.472,0.903)
Inconsistency measures: Not applicable
Incoherence judgment: Major concerns

Comparison Control:Fones
Evidence: direct
Direct standardised mean difference: 0.005(-0.355,0.365)
Inconsistency measures: Not applicable
Incoherence judgment: Major concerns

Comparison Control:Scrub
Evidence: indirect
Indirect standardised mean difference: 0.316(-0.476,1.109)
Inconsistency measures: Not applicable
Incoherence judgment: Major concerns

Comparison Fones:Scrub
Evidence: indirect
Indirect standardised mean difference: 0.311(-0.482,1.105)
Inconsistency measures: Not applicable
Incoherence judgment: Major concerns

Overall rating of the confidence into the evidence

Comparison	Number of Studies	Within-study bias	Reporting bias	Indirectness	Imprecision	Heterogeneity	Incoherence	Confidence rating	Reason(s) for downgrading
				Mixed evidence					
Bass vs Control	3	Some concerns	High risk	No concerns	Major concerns	No concerns	Some concerns	Very low	| Within-study bias | Reporting bias | Imprecision | Incoherence |
Bass vs Fones	3	Some concerns	Low risk	No concerns	Major concerns	No concerns	No concerns	Very low	| Within-study bias | Imprecision |
Bass vs Scrub	1	Major concerns	Low risk	Major concerns	Major concerns	No concerns	Major concerns	Very low	| Within-study bias | Indirectness | Imprecision | Incoherence |
Control vs Fones	4	Some concerns	Low risk	Some concerns	Major concerns	No concerns	Major concerns	Very low	| Within-study bias | Indirectness | Imprecision | Incoherence |
				Indirect evidence					
Control vs Scrub	--	Some concerns	Low risk	Major concerns	Major concerns	No concerns	Major concerns	Very low	| Within-study bias | Indirectness | Imprecision | Incoherence |
Fones vs Scrub	--	Some concerns	Low risk	Major concerns	Major concerns	No concerns	Major concerns	Very low	| Within-study bias | Indirectness | Imprecision | Incoherence |

7. CINeMA-Output: Secondary Gingivitis (studies with adults only)

	study	year	feature	outcome	index	t1	n1	y1	sd1	rob	indirectness	t2	n2	y2	sd2
1	Harnacke et al. (2012)	2012	gingivitis	secondary	PBI	Control	19	17.58	14.74	2	1	Fones	18	22.23	25.56
2	Harnacke et al. (2012)	2012	gingivitis	secondary	PBI	Control	19	17.58	14.74	2	1	Bass	19	18.57	12.67
3	Harnacke et al. (2012)	2012	gingivitis	secondary	PBI	Fones	18	22.23	25.56	2	1	Bass	19	18.57	12.67
4	Harnacke et al. (2016)	2016	gingivitis	secondary	BOP	Control	22	11.79	6.65	1	1	Fones	23	10.5	6.91
5	Harnacke et al. (2016)	2016	gingivitis	secondary	BOP	Control	22	11.79	6.65	1	1	Bass	24	11.15	8.4
6	Harnacke et al. (2016)	2016	gingivitis	secondary	BOP	Fones	23	10.5	6.91	1	1	Bass	24	11.15	8.4
7	Schmalz et al. (2018)	2018	gingivitis	secondary	GI	Control	22	1.03	0.11	2	2	Fones	22	0.96	0.11
8	Janakiram et al. (2020)	2020	gingivitis	secondary	GI	Control	40	0.9	0.61	2	2	Fones	40	1.1	0.5
9	Janakiram et al. (2020)	2020	gingivitis	secondary	GI	Control	40	0.9	0.61	2	2	Bass	40	0.9	0.4
10	Janakiram et al. (2020)	2020	gingivitis	secondary	GI	Fones	40	1.1	0.5	2	2	Bass	40	0.9	0.4

Node size by: Sample size **Node color by:** Risk of Bias **Edge width by:** Sample Size **Edge color by:** Average RoB

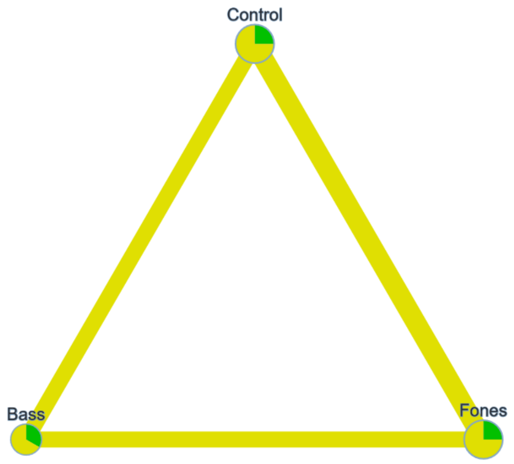

Risk of bias contributions: The bar chart shows the contributions of each piece of study to the network estimate

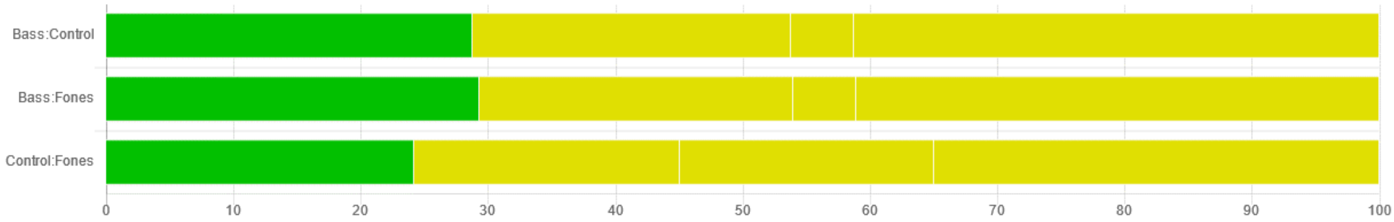

Selected Rule: *Average RoB*

Comparison **Bass:Control**
Evidence: mixed
Majority RoB: Some concerns
Average RoB: **Some concerns**
Highest RoB: Some concerns
NMA judgment Some concerns

Comparison **Bass:Fones**
Evidence: mixed
Majority RoB: Some concerns
Average RoB: **Some concerns**
Highest RoB: Some concerns
NMA judgment Some concerns

Comparison **Control:Fones**
Evidence: mixed
Majority RoB: Some concerns
Average RoB: **Some concerns**
Highest RoB: Some concerns
NMA judgment Some concerns

Reporting Bias: The Comparison Bass:Control was manually set to high risk (rationale: statistics from Dosumo et al. 2019 and Ausenda et al. 2019 were not available for NMA)

Comparison Bass:Control	Comparison Bass:Fones	Comparison Control:Fones
Evidence: mixed	Evidence: mixed	Evidence: direct
Reporting bias judgment: High risk	Reporting bias judgment: Low risk	Reporting bias judgment: Low risk

Indirectness contributions

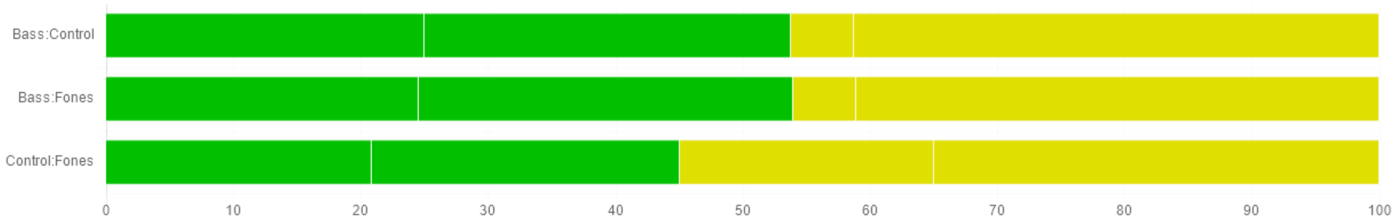

Selected rule: *Average*

Comparison Bass:Control	Comparison Bass:Fones	Comparison Control:Fones
Evidence: mixed	Evidence: mixed	Evidence: mixed
Majority: No concerns	Majority: No concerns	Majority: Some concerns
Average: No concerns	Average: No concerns	Average: Some concerns
Highest: Some concerns	Highest: Some concerns	Highest: Some concerns
NMA judgment: No concerns	NMA judgment: No concerns	NMA judgment: Some concerns

Imprecision: Clinically important effect size set at 0.2

Comparison Bass:Control	Comparison Bass:Fones	Comparison Control:Fones
Evidence: mixed	Evidence: mixed	Evidence: mixed
NMA estimate: -0.101	NMA estimate: -0.096	NMA estimate: 0.005
95% Confidence interval: (-0.495,0.294)	95% Confidence interval: (-0.491,0.300)	95% Confidence interval: (-0.355,0.365)
*Confidence interval extends into clinically important effects in **both** directions*	*Confidence interval extends into clinically important effects in **both** directions*	*Confidence interval extends into clinically important effects in **both** directions*
Imprecision judgment: Major concerns	Imprecision judgment: Major concerns	Imprecision judgment: Major concerns

Heterogeneity

The estimated value of between-study variance for the network meta-analysis is **0.052**

Comparison	Bass:Control
**Evidence: mixed**	
NMA estimate:	-0.101
**95% intervals for NMA estimate**	
Confidence interval:	(-0.495,0.294)
Prediction interval:	(-0.948,0.746)
*Confidence and prediction intervals **agree** in relation to clinically important effect*	
Heterogeneity judgment	No concerns

Comparison	Bass:Fones
**Evidence: mixed**	
NMA estimate:	-0.096
**95% intervals for NMA estimate**	
Confidence interval:	(-0.491,0.300)
Prediction interval:	(-0.943,0.752)
*Confidence and prediction intervals **agree** in relation to clinically important effect*	
Heterogeneity judgment	No concerns

Comparison	Control:Fones
**Evidence: mixed**	
NMA estimate:	0.005
**95% intervals for NMA estimate**	
Confidence interval:	(-0.355,0.365)
Prediction interval:	(-0.810,0.820)
*Confidence and prediction intervals **agree** in relation to clinically important effect*	
Heterogeneity judgment	No concerns

Incoherence

Global test based on a random-effects design-by-treatment interaction model

χ^2 **statistic: 5.608 (1 degrees of freedom), P value: 0.018**

Comparison	Bass:Control
**Evidence: mixed**	
NMA standardised mean difference:	-0.101(-0.495,0.294)
Direct standardised mean difference:	-0.012(-0.418,0.395)
Indirect standardised mean difference:	-1.567(-3.218,0.084)
**Inconsistency measures**	
Difference of standardised mean differences:	1.555(-0.145,3.255)
P value:	0.073
Incoherence judgment	Some concerns

Comparison	Bass:Fones
**Evidence: mixed**	
NMA standardised mean difference:	-0.096(-0.491,0.300)
Direct standardised mean difference:	-0.187(-0.595,0.220)
Indirect standardised mean difference:	1.392(-0.250,3.034)
**Inconsistency measures**	
Difference of standardised mean differences:	-1.579(-3.271,0.113)
P value:	0.067
Incoherence judgment	No concerns

Comparison	Control:Fones
**Evidence: direct**	
Direct standardised mean difference:	0.005(-0.355,0.365)
**Inconsistency measures:** Not applicable	
Incoherence judgment	Major concerns

Overall rating of the confidence into the evidence

Comparison	Number of Studies	Within-study bias	Reporting bias	Indirectness	Imprecision	Heterogeneity	Incoherence	Confidence rating	Reason(s) for downgrading
				Mixed evidence					
**Bass vs Control**	3	Some concerns	High risk	No concerns	Major concerns	No concerns	Some concerns	Very low	| Within-study bias | Reporting bias | Imprecision | Incoherence |
**Bass vs Fones**	3	Some concerns	Low risk	No concerns	Major concerns	No concerns	No concerns	Very low	| Within-study bias | Imprecision |
**Control vs Fones**	4	Some concerns	Low risk	Some concerns	Major concerns	No concerns	Major concerns	Very low	| Within-study bias | Indirectness | Imprecision | Incoherence |
