# Supplementary material for: Manual toothbrushing techniques for plaque removal and the prevention of gingivitis—A systematic review with network meta-analysis
Source: PLoS One. 2024 Jul 5;19(7):e0306302. doi: 10.1371/journal.pone.0306302 (PMC11226064; doi:10.1371/journal.pone.0306302)
Supplement: S5 Appendix — When a study assessed more than one plaque or gingivitis index, only one was included in the NMA and depicted in Tables 4 and 6. This appendix shows the effect sizes of the treatment comparisons regarding the alternative plaque or gingivitis index not shown in Tables 4 and 6. (PDF) [file pone.0306302.s005.pdf]

Table S1: Comparison of additionally assessed plaque indices that were not part of the NMA according to the study protocol specifications

			1st study arm compared				2nd study arm compared				standardized mean difference				
Outcome date/Study	index	weeks after intervention	Intervention	n*	mean$^+$	SD$^+$	Intervention	n*	mean$^+$	SD$^+$	SMD	SE	CI (95%) lb	CI (95%) ub	p
**Primary outcome date (Last date of assessment)**															
Deinzer et al. natural teeth [40]	TQHI	12	NST	30	1.79	0.56	Fones	32	1.87	0.52	-0.15	0.25	-0.65	0.35	0.565
			NST	30	1.79	0.56	Bass	30	1.63	0.43	0.32	0.26	-0.19	0.83	0.223
			Fones	32	1.87	0.52	Bass	30	1.63	0.43	0.50	0.26	-0.01	1.00	0.055
Deinzer et al. crowned teeth [40]	TQHI	12	NST	30	0.91	0.49	Fones	32	0.9	0.48	0.02	0.25	-0.48	0.52	0.936
			NST	30	0.91	0.49	Bass	30	0.93	0.7	-0.03	0.26	-0.54	0.47	0.899
			Fones	32	0.9	0.48	Bass	30	0.93	0.7	-0.05	0.25	-0.55	0.45	0.845
Harnacke et al. 2012 [41]	TQHI	28	NST	19	1.86	0.52	Fones	19	1.68	0.49	0.35	0.33	-0.29	0.99	0.286
			NST	19	1.86	0.52	Bass	18	1.97	0.54	-0.20	0.33	-0.85	0.44	0.538
			Fones	19	1.68	0.49	Bass	18	1.97	0.54	-0.55	0.34	-1.21	0.11	0.100
**Secondary outcome date (First date of assessment)**															
Deinzer et al. natural teeth [40]	TQHI	6	NST	30	1.94	0.59	Fones	32	1.88	0.6	0.10	0.25	-0.40	0.60	0.695
			NST	30	1.94	0.59	Bass	30	1.73	0.49	0.38	0.26	-0.13	0.89	0.142
			Fones	32	1.88	0.6	Bass	30	1.73	0.49	0.27	0.26	-0.23	0.77	0.291
Deinzer et al. crowned teeth [40]	TQHI	6	NST	30	1.03	0.61	Fones	32	0.96	0.51	0.12	0.25	-0.38	0.62	0.628
			NST	30	1.03	0.61	Bass	30	1.1	0.72	-0.10	0.26	-0.61	0.40	0.689
			Fones	32	0.96	0.51	Bass	30	1.1	0.72	-0.22	0.25	-0.72	0.28	0.382
Harnacke et al. [41]	TQHI	6	NST	19	2.03	0.55	Fones	18	1.71	0.44	0.63	0.34	-0.03	1.29	0.063
			NST	19	2.03	0.55	Bass	19	2.02	0.46	0.02	0.32	-0.62	0.66	0.953
			Fones	18	1.71	0.44	Bass	19	2.02	0.46	-0.67	0.34	-1.34	-0.01	0.046

*number of participants analyzed (for total n and drop outs see Appendix 2);$^+$Values retreived from the authors; SD: Standard deviation; SMD: standardized mean difference (Hedges 1981); SE: standard errors of the observed standardized mean difference; CI (95%) lb: lower border of the 95% confidence interval of SMD (Hedges 1981); CI (95%) ub: upper border of the 95% confidence interval of SMD (Hedges, 1981); p: probability value of corresponding SMD; NST: no specific technique

Table S2: Comparison of additionally assessed gingivitis indices that were not part of the NMA according to the study protocol specifications

			1st study arm compared				2nd study arm compared				standardized mean difference				
Outcome date/St	index	weeks after intervention	Intervention	n*	mean	SD	Intervention	n*	mean	SD	SMD	SE	CI (95%) lb	CI (95%) ub	p
**Primary outcome date (Last date of assessment)**															
Schmalz et al. [50]	PBI		NST	22	0.58	0.44	Fones	22	0.53	0.35	0.12	0.30	-0.47	0.72	0.6824
**Secondary outcome date (First date of assessment)**															
Schmalz et al. [50]	PBI		NST	22	0.58	0.32	Fones	22	0.48	0.44	0.26	0.30	-0.34	0.85	0.3991

*number of participants analyzed (for total n and drop outs see Appendix S2); SD: Standard deviation; SMD: standardized mean difference[32]; SE: standard errors of the observed standardized mean difference; CI (95%) lb: lower border of the 95% confidence interval of SMD[32]; CI (95%) ub: upper border of the 95% confidence interval of SMD [32; p: probability value of corresponding SMD; NST: no specific technique; NST: No specific technique; TQHI: Turesky's modification of the Quigley and Hein Index; PBI: Papillary bleeding index.
